# Identification of fruit size associated quantitative trait loci featuring SLAF based high-density linkage map of goji berry (*Lycium* spp.)

**DOI:** 10.1186/s12870-020-02567-1

**Published:** 2020-10-15

**Authors:** Fazal Rehman, Haiguang Gong, Zhong Li, Shaohua Zeng, Tianshun Yang, Peiyan Ai, Lizhu Pan, Hongwen Huang, Ying Wang

**Affiliations:** 1 Key Laboratory of South China Agricultural Plant Molecular Analysis and Genetic Improvement, Guangdong Provincial Key Laboratory of Applied Botany, South China Botanical Garden, Chinese Academy of Sciences, Guangzhou, 510650 China; 2 University of Chinese Academy of Sciences, Beijing, 100049 China; 3 Center of Economic Botany, Core Botanical Gardens, Chinese Academy of Sciences, Guangzhou, 510650 China; 4 Bairuiyuan Company, Yinchuan, 750000 Ningxia China; 5 GNNU-SCBG Joint Laboratory of Modern Agricultural Technology, College of Life Sciences, Gannan Normal University, Ganzhou, 341000 Jiangxi China

**Keywords:** Goji, SLAF-seq, Fruit size, QTL mapping, Interspecific, High-density genetic map

## Abstract

**Background:**

Goji (*Lycium* spp., 2n = 24) is a fruit bearing woody plant popular as a superfood for extensive medicinal and nutritional advantages. Fruit size associated attributes are important for evaluating small-fruited goji berry and plant architecture. The domestication traits are regulated quantitatively in crop plants but few studies have attempted on genomic regions corresponding to fruit traits.

**Results:**

In this study, we established high-resolution map using specific locus amplified fragment (SLAF) sequencing for de novo SNPs detection based on 305 F_1_ individuals derived from *L. chinense* and *L. barbarum* and performed quantitative trait loci (QTL) analysis of fruit size related traits in goji berry. The genetic map contained 3495 SLAF markers on 12 LGs, spanning 1649.03 cM with 0.47 cM average interval. Female and male parents and F_1_ individuals` sequencing depth was 111.85-fold and 168.72-fold and 35.80-fold, respectively. The phenotype data were collected for 2 successive years (2018–2019); however, two-year mean data were combined in an extra year (1819). Total 117 QTLs were detected corresponding to multiple traits, of which 78 QTLs in 2 individual years and 36 QTLs in extra year. Six Promising QTLs (*qFW10–6.1*, *qFL10–2.1*, *qLL10–2.1*, *qLD10–2.1*, *qLD12–4.1*, *qLA10–2.1*) were discovered influencing fruit weight, fruit length and leaf related attributes covering an interval ranged from 27.32–71.59 cM on LG10 with peak LOD of 10.48 and 14.6% PVE. Three QTLs targeting fruit sweetness (*qFS3–1*, *qFS5–2*) and fruit firmness (*qFF10–1*) were also identified. Strikingly, various traits QTLs were overlapped on LG10, in particular, *qFL10–2.1* was co-located with *qLL10–2.1*, *qLD10–2.1* and *qLA10–2.1* among stable QTLs, harbored tightly linked markers, while *qLL10–1* was one major QTL with 14.21 highest LOD and 19.3% variance. As LG10 harbored important traits QTLs, we might speculate that it could be hotspot region regulating fruit size and plant architectures.

**Conclusions:**

This report highlights the extremely saturated linkage map using SLAF-seq and novel loci contributing fruit size-related attributes in goji berry. Our results will shed light on domestication traits and further strengthen molecular and genetic underpinnings of goji berry; moreover, these findings would better facilitate to assemble the reference genome, determining potential candidate genes and marker-assisted breeding.

## Background

*Lycium barbarum* L. and *L. chinense* Mill., two closely related Solanaceae species commonly known as goji, wolfberry or boxthorn. These are deciduous woody perennial shrubs, growing up to 4 m in height, widely distributed under warm and subtropical climate in East Asia, Southeast Asia and Europe [1–3]. It is used both in fresh and dried forms, while dry root bark and fruit also have functional importance in the medicinal industry particularly Traditional Chinese Medicine (TCM) [2]. Goji has been appreciated for its numerous health benefits as it increases the eye vision, inhibits the growth of cancer cells [4, 5], anti-fatigue, anti-aging, enhance metabolism and ultimately improves the immune system [1, 6]. Physiologically, the fruit of these plants contains many compounds of functional characteristics such as vitamin C, carotenoids, flavonoids and polysaccharides [3]. Ningxia as “*Daodi*” region of goji production, widely renowned in China. Somehow, due to recent market demand goji cultivation areas have stretched to new regions over different climatic zones covering from 82°E and 115°E to 30^o^N and 45^o^N [7]. Particularly, cultivation of *L. barbarum* L. is largely extended to semi-arid climate temperate continental; Ningxia, Gansu, Inner Mongolia, Plateau continental; Qinghai and Continental arid climate; Xinjiang [8], whereas *L. chinense* Mill. is notably confined to temperate monsoon climate like Hubei [7, 8].

It is demonstrated that fruit weight controlling loci; *fw2.2*, *fas* and *lc* (*WUSCHEL*), contribute fruit size regulation by increasing carpel number, which influences yield acreage dramatically in tomato and other Solanaceae crops during domestication [9–12]. Also, *fw2.2* ortholog in maize as cell number regulator gene (CNR) potentially increases heterosis vigor and yield [13]. It is witnessed that fruit size traits substantially contribute to yield components [10, 13, 14]. Previous genetic mapping and QTL studies in interspecific tomato population revealed fruit weight loci *fw1.1*, *fw2.2*, *fw3.1*, *fw3.2* and *fw11.3* each contributing more than 20% phenotypic variation. Some of these effective QTLs, *fw2.2* [10], *fw3.2* [15], and *fw11.*3 [16], responsible for fruit weight variations have already been cloned in individuals’ studies. As shown, tomato fruit shape involved key QTLs, for example, *Fasciated* (*fas*) and *lc* (*WUSCHEL*) two important quantitative trait loci contributed synergistically to the locule number enhancement and collectively triggered fruit size in tomato [17, 18]. Comparatively, *L. barbarum* fruit is highly valued due to its bright red color, sweet taste and high economic returns [7]. The fruit is 2 or 3 chambered, fleshy, juicy with few or up to 15 seeds per fruit. The fruit sizes differ significantly between *L. barbarum* and *L. chinense*; ZKLC1 fruit is approximately 2.5 cm in size than *L. chinense* of 1.2 cm. Current germplasm and varieties of the goji berry are diverse in their agronomic characteristics, especially oblong or narrow leaves and fruit size round to oblong [20]. We have learned from previous similar studies within the Solanaceae family that tomato [21], pepper [22], potato [23], and eggplant [12], have contributed extensively for conventional and molecular genetic research in terms of the development of molecular markers based high-density linkage maps, and mapping of qualitative and quantitative attributes [12]. Goji, a closely-related *Lycium* species in the Solanaceae, have been utilized for conventional genetic approaches, including hybrid development, studies on qualitative and quantitative traits [24]. Thus far, goji berry has been less explored in terms of fruit genetic aspects and breeding of quality parameters except for one recent report [25]. The accessibility of the high-density genetic map, established using genomic DNA, and single nucleotide polymorphism (SNP) markers, explore numerous opportunities to the QTL mapping, gene tagging, and isolation of candidate loci in goji berry as well as comparative mapping studies with other Solanaceae species [12, 26]. Moreover, the presence of orthologous loci or genes have already been established in several reports [12, 22, 27]. Thus, we suggest that goji berry could explain the similar trend of genetic identities or represent a novel set of candidate genes triggering fruit size for future map-based cloning. However, larger fruit sized cultivars in goji berry industry is a major breeding goal and will be a breakthrough to further marketing expansion and production increase. Molecular breeding and marker-assisted selection (MAS) approaches of goji berry have not yet been attempted; moreover, it is a perennial tree plant with long duration from germination to flowering. Traditional breeding strategies limited due to intensive labor, cost and time inefficiency, which impeded cultivar improvement of goji berry [25].

Under rapid advancement of next-generation sequencing (NGS) technology, high-resolution genetic mapping and single nucleotide polymorphism (SNP) markers provide affordable tools for QTL mapping and MAS [28]. In the perennial fruit crops, the genetic map based on F_1_ population provides a robust source to locate linkage between commercial traits and DNA markers [29]. Highly heterozygous mapping population can be successfully developed by interspecific crosses, and F_1_ individuals determined under pseudo-testcross approach are widely applicable in perennial trees and forest herbs [30–32]. Recently, Gong et al. (2019) [33], constructed the first high-density genetic map of *L. barbarum* based on intraspecific F_1_ population using ddRAD-seq, which contained 23,967 SNPs with the total genetic length is 964.03 cM and average interval is 0.040 cM. QTL analysis identified 8 significant loci targeting photosynthetic traits in 2 years dataset. Importantly, QTLs linked to net photosynthetic rate (P_N_) and stomatal conductance proposed critical for the plant growth and development of goji berry [33]. SLAF-seq, a high throughput reduced representation sequencing strategy for large scale SNP discovery has been practiced due to numerous advantages [34, 35], in different crop plants including pepper [36], maize [37], walnut [38], cauliflower [39], eggplant [40], and *Salvia miltiorrhiza* [41]. Moreover, SLAF-seq is widely applied to different woody perennial tree plants and efficiently generated genetic maps based on F_1_ population [29, 33, 42]. Zhao et al. (2019) [25], reported wolfberry SNP based genetic map by SLAF-seq, which contained 6733 SNPs with the total length is 1702.45 cM and average inter-marker distance is 0.31 cM. Moreover, QTL analysis showed 55 QTLs, of which 18 QTLs detected for fruit index on LG11 and 2 significant QTLs for leaf index on two different linkage groups based on 2- and 3-years data, respectively [25].

Therefore, the current study executed SLAF-seq technology by establishing high-resolution linkage map utilizing 305 F_1_ individuals of interspecific population derived from *L. chinense* Mill. cv. Daye and *L. barbarum* L. cv. ZKLC1. The map contained 3495 high-quality SLAF markers including 15,815 SNPs with a final distance of 1649.03 cM and 0.47 cM average marker interval. The average sequencing depth of the genetic map was 111.85-fold in the female parent, 168.72-fold in the male parent and 35.80-fold in F_1_ individuals, accounting for greater depth than previously reported [25], which ensures the accuracy of markers along the genetic map. Notably, the QTL mapping analysis identified 42 significant genomic regions or potential molecular markers closely linked with corresponding fruit size-related traits. The high-density linkage map and promising QTLs pinpointed in this study are prerequisite for marker-assisted breeding and provide possible underpinnings for isolation of potential candidate genes underlying fruit and leaves associated commercial traits in goji berry.

## Results

### Morphological traits variability analysis

A total of 11 morphological traits, including fruit weight (FW), fruit length (FL), fruit diameter (FD), fruit shape index (FSI), number of fruits per end cluster (Nof/ec), number of fruits per node (Nof/n), number of seeds per fruit (Nos/f), 100 seed weight (100SW), leaf length (LL), leaf diameter (LD) and leaf area (LA), were evaluated based on 2 individual years (2018–2019) and extra year (1819). The analysis of variance (ANOVA) of morphological traits of 2 individual years showed significant differences (*P* < 0.05 or *P* < 0.01), which indicated variation among 305 F_1_ progenies under different years (Table S1; Additional file 4). The descriptive statistical analysis showed coefficient of variation (CV%) ranged between 9 to 45 for FSI and Nof/ec in 2018, and 8 to 54 for FSI, FD and Nof/ec in 2019. However, 1819 data showed that CV% varied from 7 to 39 for FD and Nof/ec (Table S2; Additional file 5). The frequency distribution histogram and box chart among all morphological traits for individual years (2018–2019) and extra year (1819) showed normal distribution (Fig. S1a-z; Additional file 1). Normality test was performed using Kolmogorov-Smirnov (K-S) goodness of fit based on the absolute distance between cumulative distribution and values ranged from 0.02 to 0.11, suggested positive normal distribution of all traits evaluated. The correlation analysis (*P* < 0.05 or *P* < 0.01) revealed extreme significant positive association detected in a few comparisons among FL, FD, 100SW, Nos/f and FW; FD, FSI, 100SW, Nos/f, LL, LD, LA and FL; 100SW, Nos/f, LD, LA and FD; LL and FSI; LD, LA and 100SW; Nof/n, LL, LD, LA and Nos/f; Nof/ec, LL and Nof/n; LD, LA and LL; LA and LD or significant positive correlation between LD and FW; LL and FD; LD, LA and FSI; Nof/ec and Nos/f; LD and Nof/n. While highly significant negative correlation was detected between FSI and FD; Nof/n and 100SW; Nof/ec and 100SW (Table 1). The extremely positive correlated traits might indicate tight association among linked markers or even candidate genes due to pleiotropic effects, such as FW and FL, FD, Nos/f, and FL and FD, FSI, LL, LD, LA. This information could help determine candidate gene prediction.

**Table 1:** Combined correlation analysis among 11 agronomic attributes

	FW	FL	FD	FSI	100SW	Nos/f	Nof/n	Nof/ec	LL	LD	LA
FW	1										
FL	.657**	1									
FD	.804**	.605**	1								
FSI	0.051	.650**	−.204**	1							
100SW	.342**	.320**	.312**	0.079	1						
Nos/f	.317**	.238**	.263**	0.045	0.051	1					
Nof/n	−0.055	−0.095	−0.043	−0.069	−.237**	.163**	1				
Nof/ec	0.109	0.045	0.074	−0.003	−.152**	.125*	.520**	1			
LL	0.099	.336**	.145*	.256**	0.047	.209**	.150**	0.112	1		
LD	.144*	.297**	.228**	.134*	.172**	.276**	.122*	−0.006	.825**	1	
LA	.156**	.261**	.174**	.133*	.200**	.262**	0.077	0.004	.821**	.887**	1

** Correlation is significant at the 0.01 level (2-tailed)

* Correlation is significant at the 0.05 level (2-tailed)

*FW* fruit weight, *FL* fruit length, *FD* fruit diameter, *FSI* fruit shape index, *Nof/ec* number of fruits/end cluster, *Nof/n* number of fruits/node, *Nos/f* number of seeds/fruit, *100SW* 100 seed weight, *LL* leaf length, *LD* leaf diameter, *LA* leaf area

### Analysis of SLAF-seq data generated from F_1_ population

The sequencing data were examined to ensure the validity of the SLAF library construction. According to the goji genome size and GC content information [43], the pepper genome was selected as the reference genome utilizing self-developed predicted software and established protocol [44]. For optimal enzyme digestion the following guidelines were practiced such as; 1) lowest presence of selected digested fragments in the repetitive sequences, 2) regular distribution of digested fragments in the reference genome, 3) length and specificity of the digested fragment and specific pilot experiment system must comply uniformity, 4) the final number of digested fragments or SLAF tags must meet the expected number of tags [44, 45]. *Rsa*I and *HinC*II were used for the SLAF library construction and 221,608 SLAF tags were obtained based on the pre-SLAF experiment.

**Fig. 1:**
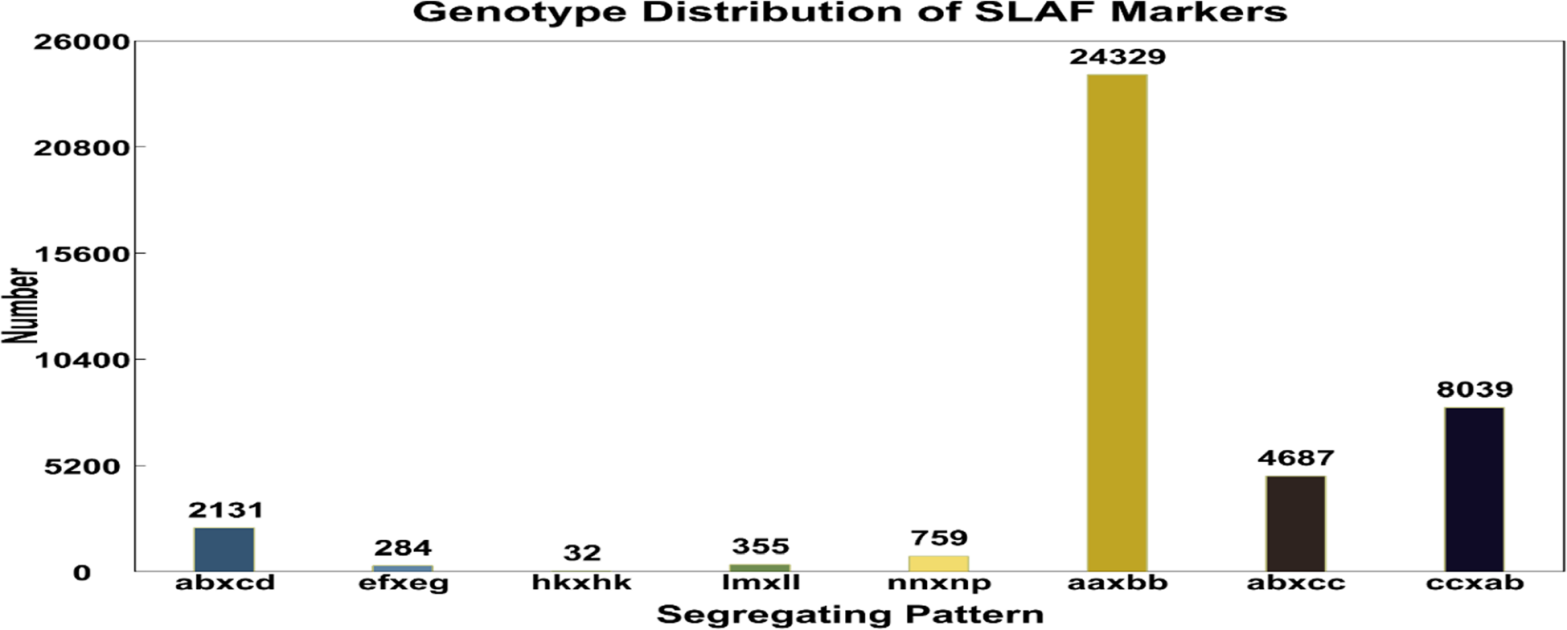
Segregation pattern of polymorphic SLAF markers for F_1_ interspecific population. *The *x*-axis depicts segregation pattern and *y*-axis shows the number of markers

**Fig. 2:**
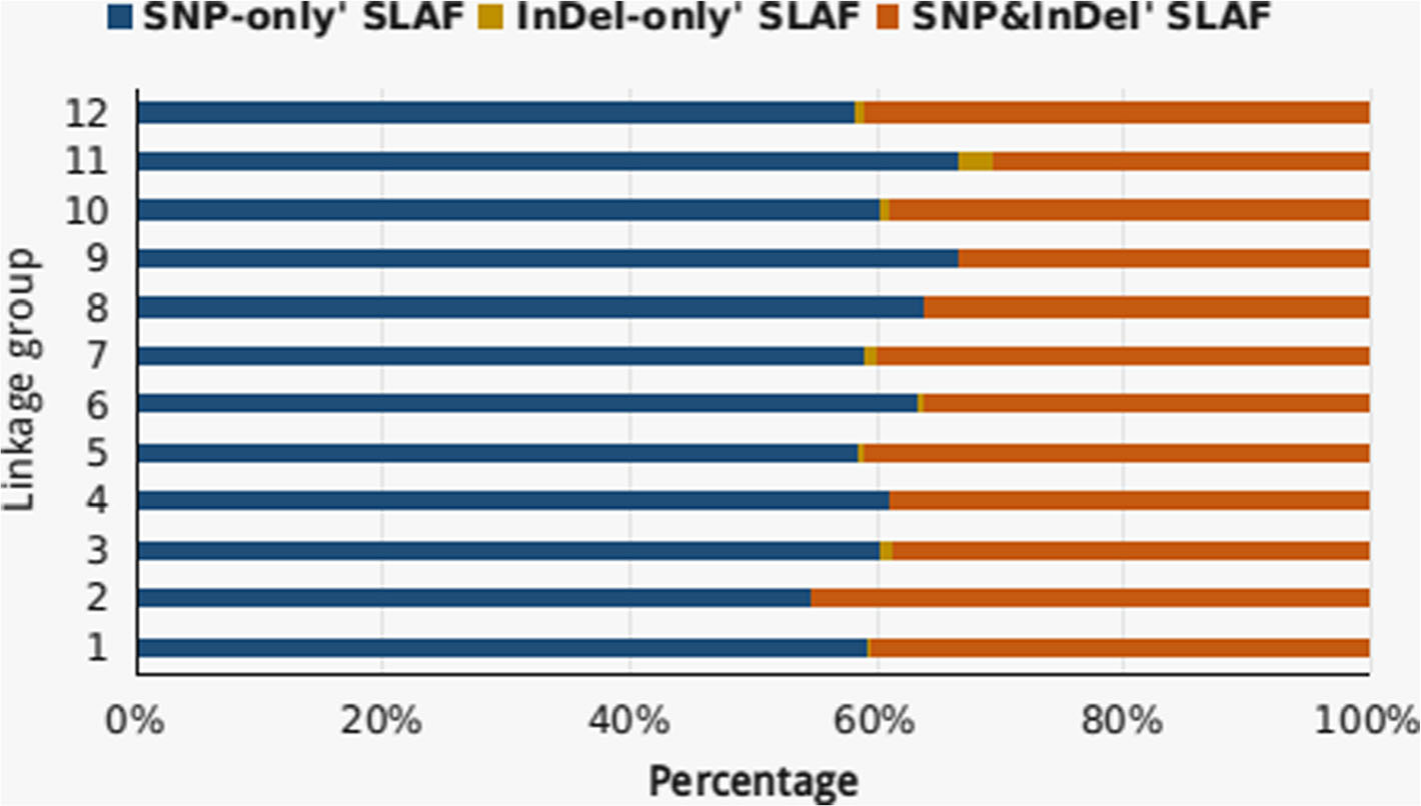
Percentages of various types of markers detected among each linkage group. *The *x*-axis indicates the percentage of three types of markers: “SNP_only, InDel_only, and SNP_InDel on each linkage group, while the *y*-axis indicates the 12 linkage groups of the integrated map

The cross-check revealed normality of the SLAF library with paired-end mapping reads of 92.16% and enzymatic digestion efficiency of 93.11%. A total of 3021.32 M clean reads of data with a length of 200 bp were generated for goji berry, comprising 37 M, 30 M and 9 M total paired-end reads in the female, male parents and offspring, respectively. The source data quality was ensured by average Q30 ratio of 95.04 and 39.62% GC. Correspondingly, in the female and male parent total number of SLAFs developed were 155,002 and 217,485 with an average depth of 72.43-fold and 60.43-fold of each SLAF marker, while for 305 F_1_ individuals 199,560 SLAFs were generated, with an average depth of 15.23-fold (Table 2). Of 494,472 high-quality SLAFs, 214,961 were polymorphic with 43.47% of polymorphism rate; furthermore, 279,356 non-polymorphic and 155 repetitive SLAFs were identified. After filtering out polymorphic SLAFs, a total of 40,616 SLAFs was classified into eight segregation patterns (Fig. 1). For the F_1_ cross-pollinated population, only 7 segregation patterns (ab×cd, ef × eg, hk × hk, lm × ll, nn × np, ab×cc, cc × ab) were retained for genetic mapping. We followed the genotyping quality criteria to filter out low quality and redundant markers using salient steps of filtering process (See “Methods” section). Collectively, we obtained 5669 total SLAFs out of which only 3495 high-quality SLAF markers were eventually utilized to construct an integrated (combined female and male parent map) high-density genetic map of goji berry with an average parental sequencing depth of more than 100-fold and offspring > 10-fold (Table 3). Among 3495 SLAF markers, ‘SNP_only’ were pre-dominant with 61.08% on the integrated map followed by ‘InDel_only’ (0.59%) and SNP & InDel (38.55%) (Fig. 2).

**Table 2:** Data description of SLAF-library Sequencing

Samples	Total Reads	Q30 (%)	GC (%)	SLAFs	Total Depth	Average Depth
Female parent	37,527,272	94.86	40.69	155,002	11,227,106	72.43
Male parent	30,768,211	94.86	39.49	217,485	13,143,686	60.43
Offspring	9,682,038	95.04	39.62	199,560	3,038,879	15.23
Control	2,233,676	95.16	40.51	–	–	–
Total	3021,316,939	95.04	39.62	–	–	–

**Table 3:** Detail Summary of high-quality SLAFs and SLAF markers

High-Quality SLAFs	
Number of *SLAFs	494,472
Average depth in female parent	72.43
Average depth in male parent	60.43
Average depth in individuals	15.23
Polymorphic *SLAFs	
Number of polymorphic *SLAFs	214,961 (43.47%)
Number of non-polymorphic *SLAFs	279,356 (56.50%)
Number of repetitive *SLAFs	155 (0.03%)
High-Quality *SLAF markers	
Number of *SLAF markers	3495
Average depth in Female parent	111.85
Average depth in male parent	168.72
Average depth in individuals	35.80

*SLAF- Specific locus amplified fragment

### Basic characteristics of the genetic map

**Fig. 3:**
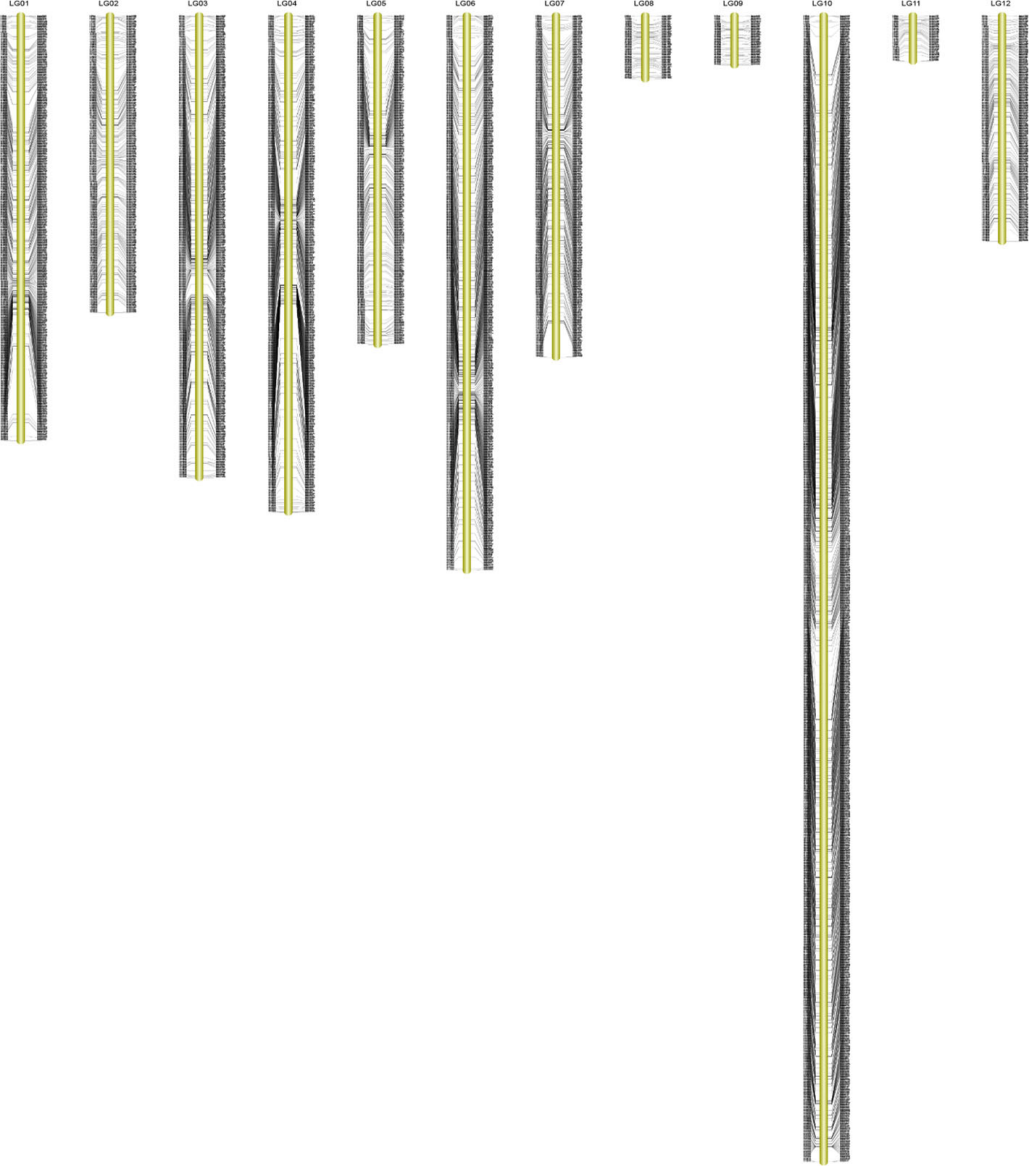
The high-density genetic map presents the distribution of SNP markers on 12 linkage groups (LGs) of goji berry (*Lycium* Spp.). *The black bars on each linkage group show markers distribution

After performing fine filtering process out of 5669 SLAFs only 3495 high-quality SLAF markers that met the quality standard were mapped onto the integrated genetic map comprised of 522 *L. barbarum* (male) and 3143 *L. chinense* (female) markers with a ratio of 61.65%. (Fig. 3, Table 4). The average coverage of SLAF markers on the integrated genetic map was 111.82 cM for female and 168.72 cM male parent and 35.80 cM each F_1_ individual, respectively. The final map was 1649.03 cM in length, confined to 12 LGs equal to the gametic chromosome number of *L. barbarum* with an average interval of 0.47 cM.

**Table 4:** Basic Characteristics of High-density genetic map of goji berry

	Number of markers			Distance (cM)			Average distance (cM)			Integrated map		SNPs		
*LGsID	*Fm	*Mm	*Im	*Fm	*Mm	*Im	*Fm	*Mm	*Im	Gaps<=5%	*Max. Gap	*SNPs	*Trv	*Tri
LG1	269	79	335	170.32	127.48	159.2	0.64	1.63	0.48	99.1	16.99	1541	575	966
LG2	220	29	234	210.97	144.49	184.2	0.96	5.16	0.79	99.14	8.85	1044	411	633
LG3	335	49	364	181.81	139.5	162.4	0.54	2.91	0.45	100	4.64	1664	640	1024
LG4	373	30	391	170.51	111.96	158.91	0.46	3.86	0.41	99.23	11.2	1874	748	1126
LG5	228	45	259	125.24	103.94	115.74	0.55	2.36	0.45	100	4.94	1190	442	748
LG6	411	43	437	204	111.81	161.19	0.5	2.66	0.37	100	3.8	2028	773	1255
LG7	237	42	269	144.18	134.76	149.38	0.61	3.29	0.56	99.63	15.07	1195	476	719
LG8	46	6	50	87.06	20.47	83.31	1.93	4.09	1.7	91.84	6.2	219	89	130
LG9	31	11	39	61.3	37.23	55.09	2.04	3.72	1.45	94.74	7.01	170	64	106
LG10	811	142	903	292.32	147.16	227.25	0.36	1.04	0.25	99.89	7.62	4013	1581	2432
LG11	36	6	36	34.65	2.15	19.16	0.99	0.43	0.55	100	2.55	163	56	107
LG12	146	40	178	226.59	97.82	173.2	1.56	2.51	0.98	98.31	9.39	709	280	429
Total	3143	522	3495	1908.95	1178.77	1649.03	0.61	2.31	0.47	98.49	16.99	15,810	6135	9675

*Fm Female map, *Mm* Male map, *Im* Integrated map, *Max* maximum, *SNPs* Single nucleotide polymorphism, *Trv* Transversions, *Tri* Transitions, *LG* linkage group

The largest and the most saturated linkage group was LG10, harbored 903 SLAF markers covering a length of 227.25 cM with the least average interval of 0.25 cM, while the smallest LG11 contained 36 markers with 19.16 cM genetic distance and 0.55 cM average interval. Surprisingly, the parental map largest linkage group was the same as that of the integrated map (LG10), harbored 142 markers spanning 147.16 cM for the male and 811 markers covering 292.32 cM for the female maps. However, LG8 and LG11 were short with an equal number of markers covering 20.47 cM and 2.15 cM genetic distances for the male map, respectively. In the same manner, LG9 belong to the female map was the smallest with 61.30 cM genetic distance (Table 4). The map uniformity revealed based on maximum gap and average gap <=5, which was experienced in LG1 with 16.99 cM and 99.10%, respectively. Moreover, we detected 15,810 SNPs ranges between 163 (LG11) to 4013 SNPs (LG10) on the integrated map along with 6135 transversions and 9675 transitions (Table 4). Gong et al. (2019) [25], constructed the integrated genetic map based on intraspecific population with 23,967 SNP markers, spanned 964.03 cM of final genetic distance and 0.040 cM average marker interval, which reflected highly saturated map [33]. Similarly, another study reported wolfberry high-density SNP based genetic map with a final distance of 1702.45 cM and 0.31 cM average interval [25]. In comparison with previous reports [25, 33], the current study revealed interspecific genetic map of goji berry with final genetic coverage of 1649.03 cM and 0.47 cM mean marker interval. In particular, LG6 & LG10 was observed with least inter marker distances indicated maximum saturation and might be considered recombination hotspots in this population.

### Assessment of high-density genetic map of goji berry

To evaluate the quality of the genetic map, we performed several mapping approaches; Firstly, the integrity of all mapped markers was carried out between 305 F_1_ individuals to ensure the quality of the genotyping, accounting for 99.03% on average (Fig. S2; Additional file 1). Secondly, haplotype mapping was performed among each individual and both parents using 3495 SLAFs to locate genotyping errors (Fig. S3a-l; Additional file 2). In the current study, the average percentage of the missing fragments were 0.0125%, represent the true quality of the map. It was verified that the interspecific F_1_ population was highly purified and suitable for high-density genetic map construction. DNA markers located on each linkage group were dispensed regularly with an average interval of 0.47 cM between adjacent markers despite high-rate of recombination events in F_1_ individuals. Thirdly, we displayed heatmaps to show the recombination frequencies among markers located on each linkage group to further evaluate the quality of the genetic map using pairwise recombination rate. The heatmaps depicted markers on the map were ordered accurately and pair-wise recombination rates were considerably low among adjacent markers and represented with yellow color diagonally. The purple color, particularly more visible in LG6 and LG10, reflected the highest recombination rates among adjacent markers (Fig. S4a-l; Additional file 2). Finally, 18 markers with segregation distortion (*P* < 0.001) were integrated in the construction of the genetic map, accounting for 0.34% of all mapped markers.

### QTL mapping analysis

**Fig. 4:**
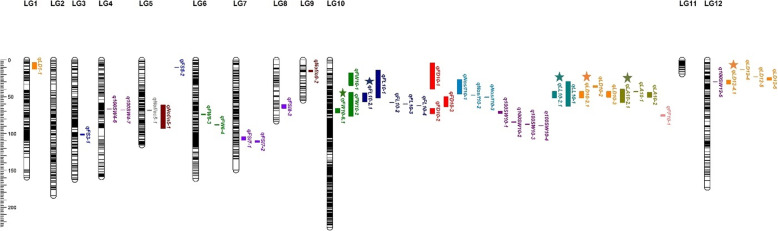
The high-density genetic map of goji berry and stable QTLs mapping on linkage groups detected under 2 individual year and extra year. *Different vertical color tiles represent various traits QTLs. Green, qFW_fruit weight; dark blue, qFL_fruit length; red, qFD_fruit diameter; blue violet, qFSI_fruit shape index; grey, qNof/ec_number of fruits per end cluster; brown, qNof/n_number of fruits per node; steel blue, qNos/f_number of seeds per fruit; dark magenta, q100SW_100 seed weight; teal, qLL_leaf length; orange, qLD_leaf diameter; olive, qLA_leaf area, blue, qFS_fruit sweetness, salmon, qFF_fruit firmness. Stars indicate stable QTLs identified in 2 individual years consistently

By using the high-density genetic map of goji berry, we plotted a large number of QTLs covering 13 traits. QTL analysis was performed using the composite interval mapping model with MapQTL v. 6.0 estimating 11 agronomical important traits for 2 individual years (2018–2019) and extra year (1819). Moreover, fruit quality traits; fruit sweetness (soluble solid content ^o^brix) FS (SSC) and fruit firmness (FF) traits were examined during summer season, 2019. In order to set the LOD threshold, we performed 1000 permutation test and the threshold value was used to evaluate the statistical significance of each QTL. In this way, significant QTLs corresponding to particular traits was identified in 2 individual years and extra year consistently. As a result, most of the QTLs including promising QTLs relating to Nos/f, FD, FW, FL and leaf related traits were positioned in linkage group 10 (Fig. 4, Table 5). Other traits QTLs such as FSI, Nof/ec, Nof/n, 100SW, FS (SSC) & FF were located in different linkage groups (Fig. S5, S6, S7). In addition, *qFS-1*, *qFS-2* corresponding to fruit sweetness with maximum phenotypic variance of 9.2% and *qFF-1* to fruit firmness with 8.1% of PVE were mapped on LG3, 5 and 10, respectively (Fig. 4 and S6, Table 5). Collectively, 117 QTLs targeting 13 important traits were found (Table 5 and S3; Additional file 6). Of which 78 QTLs in 2 individual years including 6 stable QTLs and 36 QTLs in extra year (1819) and only 3 QTLs in 2019 was detected (Table 5 and S3; Additional file 6). The number of markers located in corresponding QTLs ranged between 1 to 105, explaining highest PVE up to 19.3% with 14.21 Logarithm of Odds (LOD) covering all QTLs (Table 5 and S3; Additional file 6). QTLs detected corresponding to each trait in 2 individual years were described as follows:

- Fruit weight (*fw*): Total 9 fruit weight QTLs were detected on LG10 and 6, of which major QTL was *qFW10–1* with explained phenotypic variance of 11.1%. One stable QTL (*qFW10–6.1*) was located on LG10 with genetic distance from 65.89 to 71.86 cM and peak LOD value up to 6.01 along with corresponding PVE of 8.7% (Fig. 4). Moreover, 4 QTLs controlling FW was mapped on LG10 under extra year (1819) and *qFW10–2* was confirmed as effective loci with maximum of 11.6% PVE (Table 5 and S3, additional file 6).
- Fruit length (*fl*): 5 QTLs responsible for fruit length were found on LG10. The maximum phenotypic variance of 11.5%, corresponding to *qFL10–1* considered as significant QTL. *qFL10–2.1* targeting fruit length was observed as stable QTL on LG10, spanned an interval ranged from 44.64–56.88 cM with corresponding highest LOD of 6.54 and 9.5% phenotypic variance (Fig. 4). Another 4 FL QTLs were detected on LG10 in 1819 with highest PVE of 12.4% corresponding to *qFL10–1* (Table 5 and S3, additional file 6).
- Fruit diameter (*fd*): Only 5 QTLs corresponding to fruit diameter were located on LG10, spanned an interval ranged from 4.14–71.36 cM with a LOD value up to 7.8 and 11.1% PVE. *qFD10–1* was detected as a major QTL for controlling fruit diameter with maximum phenotypic variance of 11.1% (Table S3, additional file 6). While 3 QTLs targeting FD were also found on LG10 with highest 12.8% PVE for *qFD10–1* in 1819 (Table 5).
- Fruit shape index (*fsi*): 8 QTLs controlling fruit shape index was observed on LG1, 2, 3, 7, 8 and 11, covered genetic interval ranged from 0.85–124.76 cM with LOD between 4.55 to 7.08 and explained variance of 6.6–10.2%. The significant QTL controlling fruit shape index was *qFSI8–4* with maximum PVE of 10.2% (Table S3, additional file 6). In addition, 3 QTLs corresponding to FSI were detected on LG7 and 8 in extra year. The major QTL was *qFSI8–3* with highest phenotypic variance of 8.5% (Table 5).
- Number of fruits per end cluster (*nof/ec*): QTL analysis identified 8 QTLs targeting number of fruits per end cluster were positioned on LG5, 6, 8 and 9, spanned an interval ranged from 9.61–144.52 cM with LOD between 3 to 4.91 and 4.4 to 7.2% PVE. *qNof/ec8–3* was detected as significant QTL with larger PVE of 7.2% (Table S3, additional file 6). In 1819 only 1 QTL was detected on LG5 with 8.1% PVE for Nof/ec (Table 5).
- Number of fruits per node (*nof/n*): Total 7 QTLs corresponding to number of fruits per node were located on LG5 and 9, spanned an interval ranged from 14.05–92.48 with LOD value between 6.48 to 7.11 and 9.3 to 10.2% PVE. We detected *qNof/n5–6* as major QTL with maximum 10.2% variance (Table S3, additional file 6). Only 2 QTLs targeting Nof/n were mapped on LG5 and 9 with extreme variation of 12.5% for *qNof/n5–1* in extra year (1819) (Table 5).
- Number of seeds per fruit (*nos/f*): We detected 4 QTLs on LG10, covered an interval ranged from 32.09–51.33 cM with LOD between 4.45 to 7.72 and PVE of 6.5 to 11%. Two major QTLs (*qNos/f10–2*, *qNos/f10–3*) were observed with the highest variance of 11% (Table S3, additional file 6). Another 3 Nos/f QTLs were identified on LG10 with larger PVE of 11.6% for *qNos/f10–1* in 1819 (Table 5).
- 100 seed weight (*100sw*): 3 QTLs targeting 100 seed weight were detected on LG7, 9 and 12. These QTLs spanned an interval ranged from 14.05–100.07 with LOD value between 3.85 to 4.5 and 5.6 to 6.6% PVE. *q100SW7–2* was observed as a major QTL with maximum variance of 6.6% (Table S3, additional file 6). 7 QTLs targeting 100SW were also found on LG4, 10 and 12 with maximum PVE of 6.1% for *q100SW10–1* in extra year (Table 5).
- Leaf length (*ll*): 6 QTLs related to leaf length were mapped on LG7, LG10, while promising QTL (*qLL10–2.1*) positioned on LG10 starting from 42.77 to 51.91 cM interval bearing highest LOD of 10.48 with explained variance of 14.6% (Fig. 4). The major QTL (*qLL10–2*) was detected with maximum 14.6% PVE. Only 1 QTL (*qLL10–1*) was detected on LG10 with larger PVE of 19.3% in 1819 (Table 5 and S3, additional file 6).
- Leaf diameter (*ld*): We observed 14 fruit diameter controlling QTLs were spanned on LG1, 10, 12, among them *qLD10–2.1* and *qLD12–4.1* represented stability throughout 2 individual years and positioned on LG10 and LG12 with covered distance ranged from 42.77–51.91 cM and 27.32–32.65 cM and corresponding highest LOD of 6.64 and 5.91 and explaining PVE% of 9.5 and 8.5, respectively (Fig. 4). A *qLD10–1* having maximum variance of 11%, considered as major QTL. Another 6 LD QTLs were located on LG1, 10 and 12 with highest PVE of 11.5% for *qLD10–3* in extra year (Table 5 and S3, additional file 6).
- Leaf area (*la*): 9 QTLs were found related to leaf area on LG1, 9, 10 and 12. Among them, *qLA10–2.1* stable QTL targeting leaf area was detected on LG10 and covered genetic interval ranged from 39.84–58.12 cM with peak LOD of 7.74 and 11% explained variance (Fig. 4). Moreover, *qLA10–1* was regarded as a major QTL based on maximum phenotypic variance of 12.5%. Only 2 QTLs corresponding to LA were found on LG10 with highest PVE of 5.4% for *qLA10–2* in extra year (1819) (Table 5 and S3, additional file 6).

**Table 5:** Promising QTLs detail information detected under 2 individual years and extra year (1819) data

Traits	Year	QTLs	LGs	Interval (cM)			LOD	PVE (%)
				Start (cM)	End (cM)	distance (cM)		
*FW	2018/2019	qFW10–6.1	10	65.892	71.858	5.97	4.72–6.01	6.9–8.7
	1819	qFW10–1	10	17.483	34.771	17.29	6.51	9.4
	1819	qFW10–2	10	44.095	76.613	32.52	8.17	11.6
	1819	qFW6–3	6	73.682	74.556	0.87	5.4	7.8
	1819	qFW6–4	6	87.691	88.482	0.79	5.59	8.1
*FL	2018/2019	qFL10–2.1	10	44.638	56.88	12.24	6.21–6.54	9–9.5
	1819	qFL10–1	10	13.739	51.909	38.17	8.88	12.4
	1819	qFL10–2	10	57.755	58.118	0.36	5.63	8.5
	1819	qFL10–3	10	59.799	59.799	0	5.87	8.5
	1819	qFL10–4	10	62.134	62.134	0	5.86	8.5
*FD	1819	qFD10–1	10	4.137	39.835	35.7	9.07	12.8
	1819	qFD10–2	10	65.892	73.183	7.29	7.43	10.6
	1819	qFD10–3	10	49.704	63.503	13.8	7.02	10.1
*FSI	1819	qFSI7–1	7	104.247	108.987	4.74	5.51	8
	1819	qFSI7–2	7	109.885	111.761	1.88	5.45	7.9
	1819	qFSI8–3	8	60.56	65.763	5.2	5.89	8.5
*Nof/ec	1819	qNof/ec5–1	5	67.85	69.165	1.32	5.56	8.1
*Nof/n	1819	qNof/n5–1	5	61.076	92.992	31.92	8.86	12.5
	1819	qNof/n9–2	9	14.055	16.446	2.390	6.71	9.6
*Nos/f	1819	qNos/f10–1	10	26.713	46.338	19.62	8.15	11.6
	1819	qNos/f10–2	10	48.095	48.095	0	6.22	9
	1819	qNos/f10–3	10	50.464	50.744	0.28	5.71	8.3
*100SW	1819	q100SW10–1	10	69.787	72.522	2.73	4.16	6.1
	1819	q100SW10–2	10	84.051	84.847	0.80	3.26	4.8
	1819	q100SW10–3	10	87.238	88.035	0.80	3.32	4.9
	1819	q100SW10–4	10	89.231	89.231	0	3.21	4.7
	1819	q100SW12–5	12	30.03	30.03	0	3.03	4.5
	1819	q100SW4–6	4	66.739	66.739	0	3.01	4.4
	1819	q100SW4–7	4	67.808	67.808	0	3	4.4
*LL	2018/2019	qLL10–2.1	10	42.768	51.909	9.14	8.71–10.48	12.3–14.6
	1819	qLL10–1	10	29.322	62.784	33.46	14.21	19.3
*LD	2018/2019	qLD10–2.1	10	42.768	51.909	9.14	4.3–6.64	6.3–9.5
	2018/2019	qLD12–4.1	12	27.316	32.654	5.34	3.44–5.91	5.1–8.5
	1819	qLD1–1	1	3.65	12.708	9.06	6.98	10
	1819	qLD10–2	10	34.771	37.327	2.56	6.8	9.8
	1819	qLD10–3	10	42.768	50.744	7.98	8.1	11.5
	1819	qLD12–4	12	13.421	13.421	0	6.04	8.7
	1819	qLD12–5	12	23.146	23.146	0	6.11	8.8
	1819	qLD12–6	12	24.122	27.342	3.22	7.17	10.3
*LA	2018/2019	qLA10–2.1	10	39.835	58.118	18.28	6.46–7.74	9.3–11
	1819	qLA10–1	10	42.768	43.567	0.8	3.33	4.9
	1819	qLA10–2	10	44.095	50.744	6.65	3.71	5.4
*FS (SSC)	2019	qFS3–1	3	100.85	102.109	1.26	5.48	9.1
	2019	qFS5–2	5	10.18	10.18	0	5.4	9.1
*FF	2019	qFF10–1	10	74.412	76.613	1.2	4.81	8.1

*FW fruit weight, *FL* fruit length, *FD* fruit diameter, *FSI* fruit shape index, *Nof/ec* number of fruits/end cluster, *Nof/n* number of fruits/node, *Nos/f* number of seeds/fruit, *100SW* 100 seed weight, *LL* leaf length, *LD* leaf diameter, *LA* leaf area, *FS (SSC)* fruit sweetness (soluble solid contents), *FF* fruit firmness

In our results, *qLL10–1* was regarded as a major QTL with the largest explained 19.3% of variance and significant LOD of 14.21 (Table 5). Intriguingly, we encountered an interesting phenomenon of QTL co-localization among fruit and leaf related traits. Specifically, *qFL10–2.1* was overlapped with tightly linked markers corresponding to LL, LD & LA among stable QTLs and represented a highly significant positive correlation (Tables 1 and 5). The proportion of variance (PVE%) between promising QTLs was > 10%, which indicated major contribution of stable QTLs controlling corresponding important traits. Moreover, we had noticed a number of co-located QTLs such as fruit weight QTLs shared closely linked map positions with FL, FD, Nos/f, 100SW, LL, LD, LA and FF QTLs on LG10 consistently in 2 individuals year (2018–2019) and extra year (1819), which also indicated highly positive significant correlation as well (Fig. 4, Tables 1 and 5).

## Discussion

### SLAF based high-density mapping

A high-density and high-quality genetic map of goji berry assembled in the present study based on F_1_ population derived from 2 different species of *Lycium* using SLAF-seq strategy, demonstrated to be a highly cost-effective technique for the discovery of a large number of SNPs and wide-scale genotyping [34]. SLAF-seq has been utilized in many different crop species, even those without reference genome [33, 42, 46]. Gong et al. (2019) [33], reported very first goji berry high-density genetic map using an intra-specific F_1_ population under ddRAD-seq. While the current perpetual report on genetic mapping using SLAF-seq will further enhance the technology and improve understanding of molecular genetics of goji berry. Though, SLAF-seq approach has rarely been utilized in goji berry to construct a genetic map for QTL analysis. Comparatively, previous GBS based approaches such as RAD and ddRAD-seq have limitation due to technical and procedural complexity, cost unfeasibility and lack of pre-designed scheme and deep sequencing for ensuring accuracy and efficiency [46]. Subsequently, SLAF sequencing takes advantage of high-throughput, cost-effective, deep sequencing technique based on double Barcoding system accompanying a larger population and pre-designed strategy for large scale SNPs, InDel detection and genotyping [34].

In the first place, we contrived a scheme along the foundation of well-assembled reference genome sequence. Due to unavailability of goji genome, we explored goji genetic and DNA content detail [43], and pepper was selected as a reference genome to carry out the sequencing and ensure the uniformity, density and efficiency of markers production. Under the pilot experiment, *Rsa*I and *HinC*II restriction enzymes were chosen and excised fragment (364-414 bp) for sequencing. The SLAF library was evaluated by choosing the rice genome (*Oryza sativa L. japonica*) as a control for comparing with reference genome to ensure reliability and validity. According to Sun et al. (2013) [34], the recommended quality scores should not be less than Q30 and 6-fold minimal sequencing depth for each individual in SLAF-seq defined pipeline. Our dataset contained 3021.32 Mb paired-end reads and achieved 95.04% Q30 score. The sequencing depth for parents was 66.43-fold and 15.23-fold for individuals, which fulfilled the quantity and quality criteria of markers needed to conduct high-density genetic mapping. At last, we abided by strict principles of filtering to get high-quality SLAF markers (See “Methods” section). Two hundred fourteen thousand nine hundred sixty-one polymorphic SLAFs were obtained with a polymorphism rate of 43.47%, higher than previously identified [25, 47, 48]. In the current study, 8 segregation types were received with 24,329 maximum number of markers accounting for (aa×bb) homozygous group, which was larger than previous reports [25, 33]. A total of 5669 high-quality SLAF markers were obtained following filtration process. All the demonstrated outstanding features represented markers accuracy, high-throughput, efficiency at a feasible cost, support further use of the SLAF sequencing technique for goji berry.

### High-density genetic map of goji berry

After building a successful SLAF library and the generation of high-quality markers. Only 3495 SLAFs were integrated properly out of 5669 total SLAF markers in the genetic map along with 15,810 SNPs using High Map software. And spanned final genetic distance of 1649.03 cM on 12 linkage groups (LGs) of goji berry, with an average marker interval of 0.47 cM. The linkage map was sufficiently saturated with the smallest mean marker interval of 0.47 cM, as compared to previous similar investigations ranged from 0.48–0.95 cM [29, 40, 48, 49]. Zhao et al. (2019) [25], reported wolfberry genetic map based on 302 F_1_ individuals using 6733 SNP markers with total genetic distance of 1702.45 cM and mean interval of 0.31 cM. The current linkage map contributed greater potential by accumulating 15,810 SNPs with outstanding attributes than previously reported map [25]. Goji has gained popularity due to economic importance and its historical use as TCM [50]. Thus, molecular breeding and genetics aspects of goji berry still need to be unfolded. In the current study, 305 F_1_ individuals were selected for high-resolution strategy, though the large number of individuals could bring higher recombination events [25, 51], and accelerate the accuracy of fine QTL positioning [52].

The reliability of the genetic map was determined by haplotype maps, heatmaps, maximum gaps, mean gaps<=5, integrity of individuals on the map and segregation distortion (*P* < 0.001). The haplotype maps and heat maps generated for each linkage group revealed SLAFs distributed in a regular manner on all 12 LGs and correctly ordered. Moreover, pair-wise recombination rates were found lower significantly among adjacent markers except LG10 and 6. The maximum gap and average gap<=5 were larger than previous study [25]. LG1, 4 and 7 were found with varied marker density and maximum gaps greater than > 10 cM. Likely, low marker density areas depict strongly homozygous regions of the goji genome, a similar trend observed by Ollitrault et al. (2012) [53]. In contrast, high-density marker areas depict the centromeric location on the chromosomes contributing larger physical distance leading to lesser genetic distance. Relatively, high-density areas may interact with the genome at some position due to interspecific heterozygosity as described by Lindner et al. (2000) [54], Ollitrault et al. (2012) [53]. The integrity of the individuals on the genetic map was 99.03%, which reflected true high-quality and consistency of the map. Meanwhile, only 18 (0.34%) segregation distortion (*P* < 0.001) markers were observed in LG11 and 12, significantly lower than previously reported [25, 42]. The segregation distortion among markers might be ascribable to the distant relationship of the parents and gametic or zygotic selection [55].

The current updated high-quality and high-density genetic map were found accurately designed and extremely saturated than earlier studies [25, 41]. Therefore, we claim that the linkage map is precise and exhibit high-throughput, which would provide insights on the molecular biology of economically important medicinal tree, plant and subsequently strong grounds for QTL mapping and candidate gene identification particularly of fruit size-related traits in goji berry. Further, it will ensure to be an essential source for comparative genomic studies [56], and aid in assembling the reference genome of goji berry.

### QTL mapping analysis for fruit size-related traits

QTL mapping using high-density genetic maps can provide a fruitful estimation in specific quantitative traits and data mining [36]. For conducting QTL analysis, different software is available limited to the crossing models such as IciMapping [57], MapQTL [58], and so on. The current study is based on F_1_ interspecific population and cross-pollinated (CP) model, which is different than other population used for mappings such as RILs, back cross and double haploids [52]. Thus, MapQTL with composite Interval mapping (IM) is definitely a suitable software for conducting QTL analysis in CP model, likewise it has been successfully utilized in different species, pepper [52], cucumber [49], and walnut [38]. Zhang et al. (2010) [59], successfully plotted QTL analysis for 8 traits in sweet cherry. In a recent report by Gong et al. (2019) [33], a total of 32 QTLs were found corresponding to six photosynthesis-related traits using MapQTL in *L. barbarum* L. Similarly, in another study the interspecific high-density genetic map of goji berry has been explored for six agronomically important traits utilizing MapQTL [25].

We employed MapQTL v. 6.0 and characterized a large set of QTLs controlling fruit and leaf-related traits and fruit quality parameters as well. Particularly, 6 promising QTLs observed on LG10 and 12, targeting FW, FL, LL, LD and LA under 2 individual years consistently. For the extra year (1819), 36 QTLs were detected corresponding to FW, FL, FD, FSI, Nof/ec, Nof/n, Nos/f, 100SW, LL, LD and LA (Fig. 4 and S6, Table 5). QTLs detected consistently in 2 years (2018–2019) dataset were regarded as stable QTL. Among several stable QTLs, fruit weight QTL (*qFW10–6.1*) was detected on LG10 with LOD up to 6.01 and explained phenotypic variance of 8.7%. In contrast, no fruit weight stable QTL was detected in previous study [25]. The fruit weight QTLs have been reported in different Solanaceae species, for example, in tomato 28 FW QTLs were identified. Specifically, *fw1.1*, *fw2.1*, *fw2.2*, *fw3.1*, *fw3.2* and *fw11.3* major fruit weight QTLs mapped in almost four interspecific individual studies and accounted for fruit weight variation during tomato evolution [21]. Similarly, 5 fruit weight QTLs were detected in pepper, and *fw3.2* was found with significant impact and larger phenotypic variance (R^2^ = 0.12–0.15) [22]. Pereira et al. (2018) [47], investigated QTLs regarding fruit morphological traits in *Cucumis melo* L. and *FWQU5.1* was detected as a major QTL on LG5 controlling fruit weight with maximum PVE of 28.3%. Another study found, 3 QTLs targeting fruit weight in eggplant on different LGs with maximum phenotypic variance of 44% corresponding to *qFW-9.1* [12]. For fruit length, we detected promising QTL (*qFL10–2.1*) on LG10 with peak LOD of 6.54 and 9.5% phenotypic variance. Zhao et al. (2019) [25] detected 4 stable FL QTLs on LG11 with LOD and PVE up to 17.24 and 30.9%, respectively. Several reports identified fruit length and fruit diameter QTLs spanned on different chromosomes [29, 40, 60].

Among the leaf related traits, *qLL10–2.1* stable QTL corresponding to leaf length was spanned on LG10, with peak LOD value and PVE up to 10.48 and 14.6%, respectively. Other stable QTLs (*qLD10–2.1* and *qLD12–4.1*) controlling leaf diameter located on LG10 and 12 with the highest LOD of 6.64 and 5.91, and 9.5 and 8.5% phenotypic variance. The stable QTL responsible for leaf area was *qLA10–2.1* spanned on LG10 with corresponding peak LOD of 7.74 and explained variance of 11%. Nonetheless, previously only two stable QTLs were reported regarding leaf index (*qLI10–2* and *qLI11–2*) on LG10 and 11, harbored 4 and 20 markers, respectively; while, no stable QTLs controlling leaf length and leaf width were reported [25]. Zhao et al. (2019) [25], detected total 55 QTLs targeting fruit weight, fruit length, fruit index, leaf width and leaf index except leaf length based on more than 2 years phenotypic data. Most of the QTLs were spanned on LG11 indicated cluster region for various traits. Moreover, 18 significant QTLs corresponding to fruit index were detected on LG11 covering an interval of 73.49–90.94 cM and one major QTL (*qFL11–3*) targeting fruit length was also observed with highest PVE of 30%. Among leaf related traits, 2 stable QTLs (*qLI10–2*, *qLI11–2*) targeting leaf index were detected on two different linkage groups for the 3-year dataset. Comparatively, in the present study, we detected 78 QTLs corresponding to all traits under investigation for 2 individual years of dataset along with 36 QTLs observed for an extra year (1819) spanned an interval ranged from 3.65–111.76 cM. In addition, 6 stable QTLs (*qFW10–3.1*, *qFL10–2.1*, *qLL10–2.1*, *qLD10–2.1*, *qLD12–4.1*, *qLA10–2.1*) were observed on LG10 and LG12 targeting fruit size-associated attributes (Fig. 4, Table 5). LG10 was observed loaded with significant QTLs represented as a hot spot region for important fruit and leaf related traits. Interestingly, we found a couple of overlapping QTLs with those detected by Zhao et al. (2019) [25] such as *qFSI1–1* and *qFI1–2* [25], located both on LG1 and spanned an interval ranged from 123.73–124.76 cM & 114.81–123.77 cM, respectively. Another QTL set (*qNof/ec5–3.1*, *qNof/n5–1.1* and *qLW5*) were overlapped on LG5 and covered genetic distance ranged from 68.04–76.61 cM [25] (Table S3, additional file 6). We also encountered colocalization phenomenon of *qFL10–2.1* with *qLL10–2.1*, *qLD10–2.1* and *qLA10–2.1* along with tightly linked markers among stable QTLs (Fig. 4, S8a-o; Additional file 3). Additionally, it was observed that QTLs corresponding to FW, FL, FD, Nos/f, LL, LD and LA shared similar genetic position on LG10 throughout two individual years as well as in extra year. The co-localization of traits linked QTLs usually reflected positive significant correlation among different traits [22]. For example, fruit diameter and pericarp thickness analysis revealed 3 common positions on the genetic map of pepper (*r* = 0.86, 0.87), reflected dual genomic regions with common interaction for both traits [22]. So, we suggest that the current findings could reveal extra potential than previous similar studies and would provide a novel foundation to understand the genetics linked to fruit size-related attributes of goji berry.

It is one assumption that QTLs detected in the same genomic region targeting various traits may occur due to pleiotropy or linkage phenomenon [22]. Among promising QTLs, only 17 markers were found closely linked with FL, LL, LD and LA. It is also worth mentioning another set of 5 markers, which were located in stable QTLs related to FL, LL and LA on LG10. In the previous similar study, 3 markers were found significantly related to several traits in pepper, for example, 1 marker (LG8) influenced six traits, another marker (LG2) associated with four traits and third marker (LG3) related with five different fruit development traits [22]. Thus, we may hypothesize that those particular five genomic regions or loci may involve either a specific locus associated with pleiotropy, which might influence different aspects of goji fruit size and plant architecture or several linked loci indicates the specific influence on plant developmental responses. Further studies will require to target these promising QTLs for determining the likely candidate genes affecting different fruit development traits in goji berry. Furthermore, fruit sweetness (SSC) QTLs (*qFS3–1*, *qFS5–2*) were detected on LG3 and LG5 with relative LOD of 5.48 and 9.2% PVE and *qFF10–1* responsible for fruit firmness were spanned on LG10 with peak LOD of 4.81 and PVE 8.1% (Fig. 4, Table 5). Ben Chaim et al. (2001) [22], reported similar events by identifying 3 QTLs in each year, but none as stable, corresponding to soluble solids concentration (SSC) in pepper with the largest phenotypic effect (*R*^*2*^ = 0.12). Two QTLs (*fi9.1*, *fi11.1*) were detected controlling fruit firmness in pepper with 14% total variance [22], which indicated close agreement with our findings. Some other study conducted QTL analysis and found 2 QTLs (*QBRX2–1*, *QBrix6*) targeting flesh sweetness in watermelon [61].

However, transcriptome sequencing analysis is too indispensable to measure the expression pattern between different fruit and leaf developmental stages, which can exacerbate the mining process of candidate gene identification [25, 49]. It has been brought out that promising orthologous QTLs or genes in similar crop species influence the same morphological or phenotypic impacts. Some physiologically associated trait comparison has been done practically and identified similar genetic positions [22, 62]. Before the 90s, there was no QTL comparison has been made relating to fruit-producing crops until the identification of fruit associated traits in tomato [21, 63]. Ben Chaim et al. (2001) [22], performed comparative QTL analysis between pepper and tomato and found quite interesting findings regarding fruit shape and fruit weight attributes. The orthologous QTLs in pepper and tomato targeting fruit weight on chromosomes 2 and 3 and one RFLP marker was found closely associated with *fw2.1* and *fw2.2* in pepper [22], and tomato [64], and another marker with *fw3.2* and *fw3.1* in pepper [22], and tomato [21], respectively. The above explanation could be helpful in the current study as Goji (*Lycium* spp.) being relative to the Solanaceae; thus, we might expect similar genetic identities or syntenic association, which existed in tomato and pepper [22, 27], tomato and eggplant [12], and tomato and potato [23], which would be uncovered in later studies. Here, we have utilized common morphological traits related to the fruit and leaf development and these featured traits found to deliver a major contribution for domestication of fruit crops [12, 56]. To further evaluate the potential of goji population and explore additional QTLs, we already have initiated developing recombinant inbred lines (RILs) by selecting full-sib mating with extremely distinguished offspring in 2019. RILs population have been successfully applied in many crop species such as pepper [52], peanut [65], and melon fruit [47]. Moreover, the enhanced QTL analysis could lead to exploring the genetic and molecular characterization of agronomically important fruit traits in goji berry.

## Conclusions

Our findings of goji berry high-resolution linkage map using specific locus amplified fragment sequencing (SLAF-seq), may inculcate a powerful and more precise technique for de novo SNPs detection and high- density genetic map construction for species without reference genome sequence. A total of 3495 high quality SLAF markers was utilized for the construction of genetic map using 305 individuals from the F_1_ interspecific population. The high-density genetic map provides 6 promising QTLs related to fruit weight, fruit length, leaf length, leaf diameter and leaf area. Remarkably, fruit weight, fruit length, fruit diameter, number of seeds per fruits and leaf related traits QTLs were shared similar location on the map, in particular, fruit length QTL (*qFL10–2.1*) were co-localized corresponding to leaf size-related stable QTLs with tightly linked markers on LG10. We might speculate that these promising QTLs have pleiotropic or linkage relationship controlling several traits. Our results will lay a solid ground for conducting positional cloning and marker-assisted breeding of goji berry. Moreover, our previous report results regarding the high-density genetic map of *L. barbarum* using intra-specific population and the current findings based on interspecific population may prove to be a better source for assembling the goji berry reference genome.

## Methods

### Plant materials

**Fig. 5:**
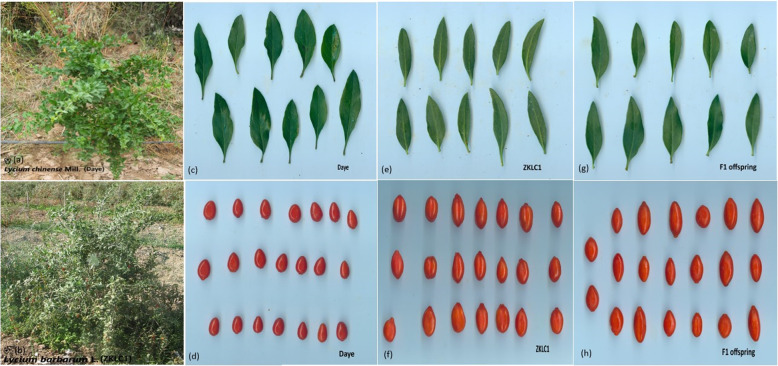
Female (*Lycium chinense* Mill. cv. Daye) and male (*Lycium barbarum* L. cv. ZKLC1) parent plants, and F_1_ offspring pictures. *a female plant, **b** male plant, **c** female plant leaves, **d** female mature fruit, **e** male plant leaves, **f** male mature fruits, **g** F_1_ offspring leaves, **h** F_1_ offspring mature fruits

The planting material was comprised of *Lycium chinense* Mill. cv. “Daye” as the female parent and *Lycium barbarum* L. cv. “Zhongkelüchuan1 (ZKLC1)” as the male parent. Two cultivated varieties ZKLC1 and Daye were obtained from Goji germplasm reservoirs of Wuhan Botanical Garden, Chinese Academy of Sciences, Wuhan and South China Botanical Garden, Chinese Academy of Sciences, Guangzhou, China, respectively. The F_1_ interspecific pseudo-testcross population was constructed by cross-fertilizing two parents in June 2016 and after sowing in the greenhouse during spring of 2017, the nursery was transplanted in the field at North-West China Bio-agricultural Center, (38°28′05″ N, 106°16′23″ E) Yinchuan City, Ningxia Province, China. The female parent (Daye), also known as Chinese Goji, botanically has ovate to lanceolate leaves, shallow to dark purple flowers, fruit color range from dark red to orange, fruit shape oblong, fruit weight (0.38 g), fruit length (1.08 cm), fruit diameter (0.75 cm) (values are taken from collecting data), and seeds typically broader, rounded than *L. barbarum* with yellowish to yellow-brown color. The male parent (ZKLC1) has lanceolate to oblong leaves, bright to royal purple flower color, shiny red fruit color, round in shape, fruit weight (1.05 g), fruit length (1.79 cm), fruit diameter (1.28 cm), and seeds semispherical to flattish with light yellow color [66], (The parents Daye and ZKLC1 and progeny plant and fruit pictures can be seen in Fig. 5). In September 2018, 305 individuals randomly selected out of total 450 offspring were used for genetic linkage map construction and QTL mapping analysis, particularly of agronomic importance, fruit size and leaf related morphological traits such as fruit weight (FW), fruit length (FL), fruit diameter (FD), number of fruits per end cluster (Nof/ec), number of fruits per node (Nof/n), number of seeds per fruit (Nos/f), 100 seed weight (100SW), and leaf length (LL), leaf diameter (LD), leaf area (LA). Despite morphological traits, fruit quality traits such as fruit sweetness-soluble solid contents ^o^brix FS (SSC) and fruit firmness (FF) were also included based on one-year dataset (2019).

### Phenotypic traits estimation

The morphological trait data were gathered during two crop seasons, the summer (July) of 2018 and 2019. For fruit and leaf related traits: FW, FL and FD, LL, LD and LA, 20 fully ripened fruits and 10 fully grown first true leaves were picked randomly from progeny and parents’ plant from the field, then photographed on scale paper (35 × 25 cm) by using Nikon D750 DSLR (Nikon Corporation, Japan) in the year 2018. The digital images were processed to measure the above-mentioned traits by ImageJ [48], software v. 1.52a [15, 16]. Conversely, for 2019 summer season, a quicker yet precise method was adopted to assess the fruits and leaves related traits. Firstly, a digital scanner (*MICROTEK* ScanMaker *i*600) was employed to photograph the fruits and leaves, then built-in Chinese software Ban Ben, WSEEN (v 2.1.2.4) used to measure FL, FD, LL, LD and LA according to the developer’s instructions. FSI was measured by dividing FL with FD. The other trait data, such as Nof/ec and Nof/n were collected visually in the field based on a maximum of 10 repeats per individual plant. Nos/f and 100SW were measured in the laboratory by taking 5 repeats and after overnight complete drying 100 seeds weighed on a digital balance (Sartorious, BSA224S). For assessing fruit quality traits: FS (SSC) ^o^brix, we collected fully ripened fruits from F_1_ population per plant 5 repeats and measured with portable refractometer Pal-1(www.atago.net/), and fruit hardness tester equipment GY-4 (Zhejiang Top Instrument Co., Ltd. Hangzhou, China) were used to estimate FF.

### DNA extraction and SLAF library construction and sequencing

Prior to DNA extraction, the young leaves from parents and 305 progenies were collected and dried with desiccant (silica gel). All the leaves DNA were extracted with Plant Genomic DNA Kit (TIANGEN BIOTECH (BEIJING), CHINA, CO., LTD.) according to the manufacturer’s instructions with slight modifications. The quantity and quality of the extracted DNA samples were evaluated with a NanoDrop ND-2000 spectrophotometer (Thermo Fisher Scientific) and electrophoresis with 1% agarose gel, respectively. Specific length amplified fragment sequencing (SLAF-seq) was performed to genotype the 305 individuals from F_1_ population and two parents according to Sun et al. (2013) [34], with minor modifications. The SLAF-seq strategy framework was adopted as the genomic DNA of each sample was subjected to restriction enzyme digestion using *Rsa*I and *Hin*CII (New England Biolabs, NEB, USA). The excised fragments were integrated with a single nucleotide A using Klenow Fragment (3′ → 5′ exo-, NEB), ligation of dual tag-labelled sequencing adapters to the A-tailed fragment was performed with T4 DNA ligase (PAGE purified, Life Technologies, USA) [52]. The PCR amplification was completed by using diluted DNA samples after ligation, Q5®, HF- DNA polymerase (NEB), dNTPs, and primers set (forward primer: 5′-AATGATACGGCGACCACCGA-3 and reverse primer: 5′-CAAGCAGAAGACGGCATACG-3). The target fragments (364-414 bp) were purified using a QIAquick gel extraction kit (Qiagen, Hilden, Germany). The purification of the PCR products was carried out using Agencourt AMPure XP beads (Beckman Coulter, High Wycombe, UK) and then pooled. The 2% agarose gel electrophoresis was used for pooled sample separation. After diluting gel-purified products, pair-end sequencing with a standard of 125 bp was performed using an Illumina Hi-Seq 2500 system (Illumina, Inc., San Diego, CA, USA) to sequence the SLAF in the quality-tested library at Biomarker Technologies Corporation, Beijing, China [44]. We used Japanese rice, *Oryza sativa L. japonica* genome as control with a genome size of 382 Mb (http://rapdb.dna.affrc.go.jp.), which claimed to verify the reliability and validity of the testing process and followed the same treatments in accordance with the goji mapping population.

### SLAF sequencing data analysis and segregation pattern grouping

The grouping and genotyping analysis of SLAF sequencing data was performed in detail as described [34]. The raw reads were identified by sequencing to locate reads of the samples through dual index barcode sequences and low-quality reads of quality score < 20e were filtered out. The clean reads were clustered together through BLAT software [67], based on the similarity of more than 90%. After trimming of barcodes and the terminal 5-bp positions from each high-quality read, which later defined as one SLAF locus [66]. From each SLAF locus SNP loci were detected among parents, and SLAFs greater than 3 SNPs filtered out. According to the parents and offspring, reads with sequencing depth > 140.28-fold and > 35.80-fold were used to define alleles corresponding to each SLAF locus, respectively. One SLAF locus can hold mostly 4 genotypes for diploid species, hence more than 4 alleles in SLAF loci considered as repetitive SLAFs and discarded later. SLAFs with 2 to 4 alleles were recognized as polymorphic and known as potential markers. All SLAFs polymorphism loci were clustered into 8 segregation patterns as follows: (ab×cd, ef × eg, hk × hk, lm × ll, nn × np, aa×bb, ab×cc, and cc × ab). As the mapping population was F_1_ interspecific from two highly heterozygous parents, thus all patterns were required to construct a high-density genetic map except (aa×bb).

A Bayesian approach was utilized for genotype scoring and quality as demonstrated [34]. To identify high-quality SLAF markers 3 steps were taken into account. Firstly, the markers with average sequencing depths in each offspring found > 35.80-fold and parents > 140.28-fold. Secondly, markers exceeding 10% missing data were discarded. Thirdly, markers with significant segregation distortion based on chi-square test (*P* < 0.001) were disqualified, but added later as additional markers during high-density genetic map construction.

### Construction of high-density genetic linkage map and evaluation

Primarily, the marker loci were segregated into 12 linkage groups (LGs) under modified logarithm of odds (MLOD) scores > 5. To construct high-quality and high-density map reliably, an advance high map strategy was required to assemble SLAF markers in a particular sequence and exact genotyping errors within linkage groups [19]. The maximum likelihood method by Van Ooijen [68], was employed to construct a high-density genetic map of goji berry and SMOOTH algorithm was utilized for genotyping error correction [69]. K-nearest neighbor algorithm was used to ascribe missing genotypes [70], and Kosambi mapping description was applied to estimate the genetic map distances between adjacent markers in centimorgan (cM) [71]. The genetic map was evaluated further following integrity of genetic maps, haplotype map, heat map and segregation distortion analysis (*P* < 0.05).

### QTL mapping analysis

The QTL mapping analysis was executed for a mean of 11 fruits and leaf size-related traits and 2 fruit quality-related attributes and analyzed by MapQTL v. 6.0 [58], which employed inclusive composite interval mapping (ICIM) to detect QTL loci among 12 linkage groups for an integrated map of goji berry. A 1000 permutation test (PT) was performed to determine the logarithm of odds (LOD) for assessing statistical significance of each QTL [72]. For reliability of determining QTLs with major and minor effect, different LOD scores were assumed. At first, a LOD threshold corresponding to 0.99 confidence was considered, and if there was no mapping interval, the threshold value corresponding to 0.95 confidence was used; if there was still no positioning interval, then the LOD threshold value of 0.90 confidence was considered. Lastly, if there was still no mapping interval detected, then the permutation test result was not carried out, and the threshold value was manually lowered to 3.0, 2.5, and 2.0. The phenotype variance percentage as elucidated by its corresponding QTL (phenotypic explained variance %) was estimated within the segregating population on the basis of population variance using MapQTL. The QTL naming criteria were followed as described by McCouch et al. (1997) [73]. The QTLs positioning and genetic map were assembled by BioMercator v. 4.2.1 [74]., and MapChart v. 2.2 [75].

### Statistical analysis

The general descriptive statistical analysis, including frequency distribution was carried out on Origin Pro v. 2016 (https://www.originlab.com/). The Analysis of variance for 2 individual years and Pearson correlation were accomplished on SPSS v. 25 software package (SPSS Inc., Chicago, IL, United States).

## Supplementary information

**Additional file 1: Figure S1(a-z).** Frequency distribution histogram of the 305 F_1_ individuals for all investigated traits based on two individual years (2018–2019), an extra year (1819) and only one year (2019) dataset. **Figure S2** The marker integrities of each individual in mapping population. *The *x*-axis indicates all 305 individuals along with the specific code name, while the *y*-axis shows markers integrity.

**Additional file 2: Figure S3(a-l).** Haplotype mapping of 305 F_1_ individuals based on 12 linkage groups of integrated maps. *In each map, horizontal line stands a marker and column shows a chromosome in a sample. The first column of the map shows a paternal chromosome and the second column as maternal chromosome, and individuals are separated by blank columns. Green color indicates first allele from the parent, blue as the second allele from the parent, white color shows not judged event, and grey indicates a missing event. The position where color changes in the same column display reorganization events. **Figure S4(a-l).** Heatmaps of 305 F_1_ individuals based on 12 linkage groups of integrated maps. *Each row and column represent marker arranged in the orderliness of the map. Every small square show recombination rate between two markers. The color change trend from yellow to red to purple display reorganization rate from small to large. Yellow color indicates closer marker recombination rate, whereas closer the color from yellow to purple farther becomes the recombination rate.

**Additional file 3: Figure S8 (a-o).** Stable QTLs map position on linkage group 10 (LG10) in 2 individual years (2018–2019) and extra year (1819). *left > 2018, center > 2019, right > 1819, FW_fruit weight, FL_fruit length, LL_leaf length, LD_leaf diameter, LA_leaf area. The red line shows phenotypic explained variation (Expl%), blue line LOD value, grey line LOD threshold value.

**Additional file 4: Table S1.** Combined analysis of variance (ANOVA) among 2 individual years (2018–2019) and 11 phenotypic traits data collected from 305 offspring of F1.

**Additional file 5: Table S2.** Descriptive statistics among 13 attributes based on 2 individual year (2018–2019) and extra year (1819) dataset.

**Additional file 6: Table S3.** All QTLs detected during 2 indivdual years 2018–2019.

**Additional file 7: Figure S5.** QTL mapping on integrated linkage map in 2018. *The different color pattern represents identified QTLs for agronomic traits of goji berry such as FW_fruit weight, FL_fruit length, FSI_fruit shape index, Nof/ec_ number of fruits per end cluster, Nof/n_ number of fruits per node, Nos/f_ number of seeds per fruit, 100SW_100 seed weight, LL_leaf length, LD_leaf diameter, LA_leaf area.

**Additional file 8: Figure S6.** QTL mapping on integrated linkage map in 2019. *The different color pattern represents identified QTLs for agronomic traits of goji berry such as FW_fruit weight, FL_fruit length, FSI_fruit shape index, Nof/ec_ number of fruits per end cluster, Nof/n_ number of fruits per node, Nos/f_ number of seeds per fruit, 100SW_100 seed weight, LL_leaf length, LD_leaf diameter, LA_leaf area, FS_ fruit sweetness (^o^brix) and FF_ fruit firmness

**Additional file 9: Figure S7.** QTL mapping on integrated linkage map in 1819. *The different color pattern represents identified QTLs for agronomic traits of goji berry such as FW_fruit weight, FL_fruit length, FSI_fruit shape index, Nof/ec_ number of fruits per end cluster, Nof/n_ number of fruits per node, Nos/f_ number of seeds per fruit, 100SW_100 seed weight, LL_leaf length, LD_leaf diameter, LA_leaf area.

## Data Availability

The raw SLAF-sequencing data reported in this paper have been deposited in the Genome Sequence Archive [76], in National Genomics Data Center [77], Beijing Institute of Genomics (China National Center for Bioinformation), Chinese Academy of Sciences, under accession number(s) CRA002920 that are publicly accessible at https://bigd.big.ac.cn/gsa. The remaining datasets used to support the conclusions of this article are included within the article and its additional files.

## Abbreviations

SLAF-seq: Specific length amplified fragments-sequencing
SNP: Single nucleotide polymorphism
cM: Centimorgan
ZKLC1: Zhongkelüchuan1
cv: Cultivar
LOD: Logarithm of odds
QTL: Quantitative Trait Loci
LG: Linkage group
MLOD: Modified Logarithm of odds
SSC: Soluble solid contents
PVE: Phenotypic variance explained
InDel: Insertion/Deletion
GBS: Genotyping by sequencing
ddRAD: Double digest restriction-site associated DNA
F_1_: First filial
MAS: Marker-assisted selection
ANOVA: Analysis of variance
ICIM: Inclusive composite interval mapping
NGS: Next generation sequencing
BLAT: BLAST like alignment tool
