# Supplementary material for: Identification of fruit size associated quantitative trait loci featuring SLAF based high-density linkage map of goji berry (Lycium spp.)
Source: BMC Plant Biol. 2020 Oct 15;20:474. doi: 10.1186/s12870-020-02567-1 (PMC7565837; doi:10.1186/s12870-020-02567-1)
Supplement: Supplementary file 1 — Additional file 1: Figure S1(a-z). Frequency distribution histogram of the 305 F1 individuals for all investigated traits based on two individual years (2018–2019), an extra year (1819) and only one year (2019) dataset. Figure S2 The marker integrities of each individual in mapping population. *The x-axis indicates all 305 individuals along with the specific code name, while the y-axis shows markers integrity. [file 12870_2020_2567_MOESM1_ESM.docx]

**
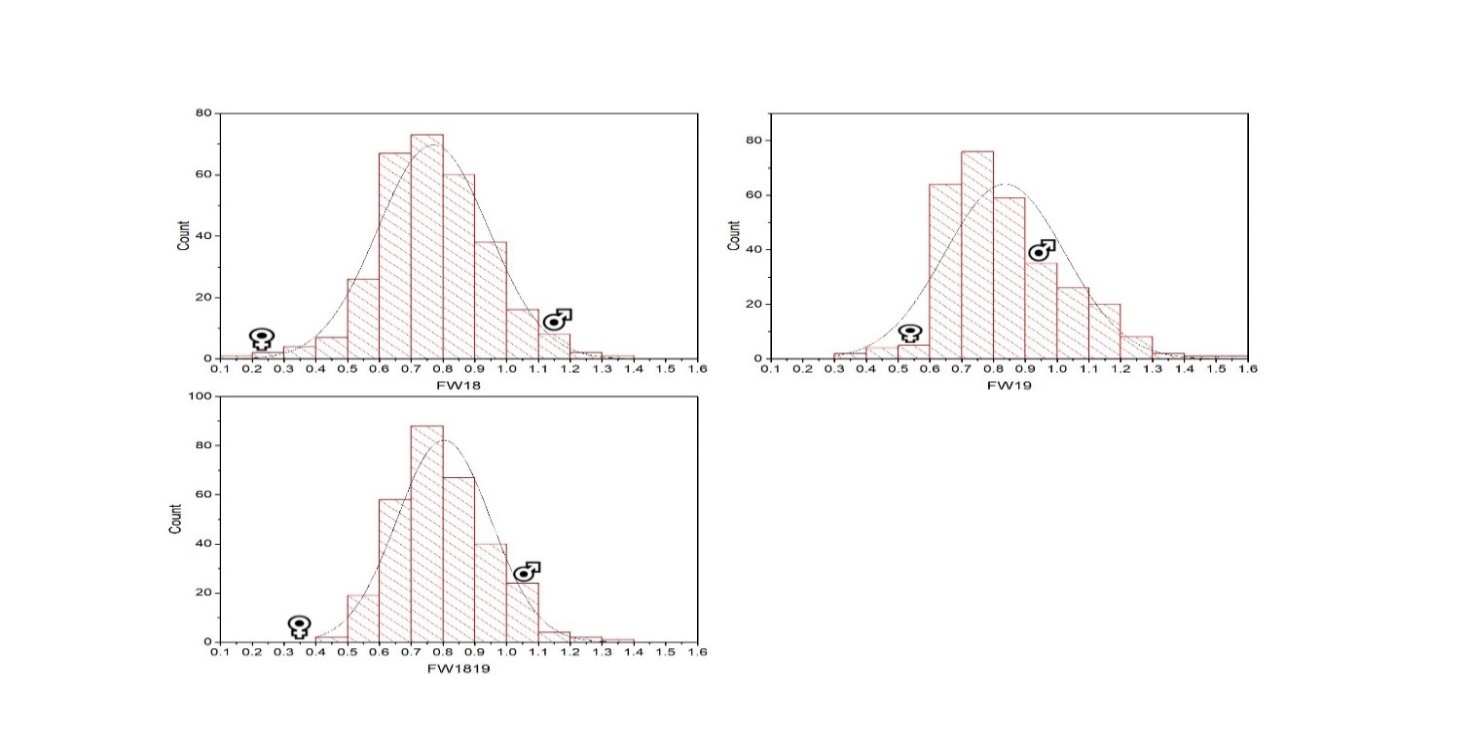
**

**S1a ***Each *x*-axis represents the value of the trait and *y*-axis shows the number of frequency corresponding with the value on *x*-axis. FW18, fruit weight (2018), FW19, fruit weight (2019), FW1819, fruit weight as extra year; ♀, indicate female parent position on the histogram, ♂, male parent position on the histogram.

**
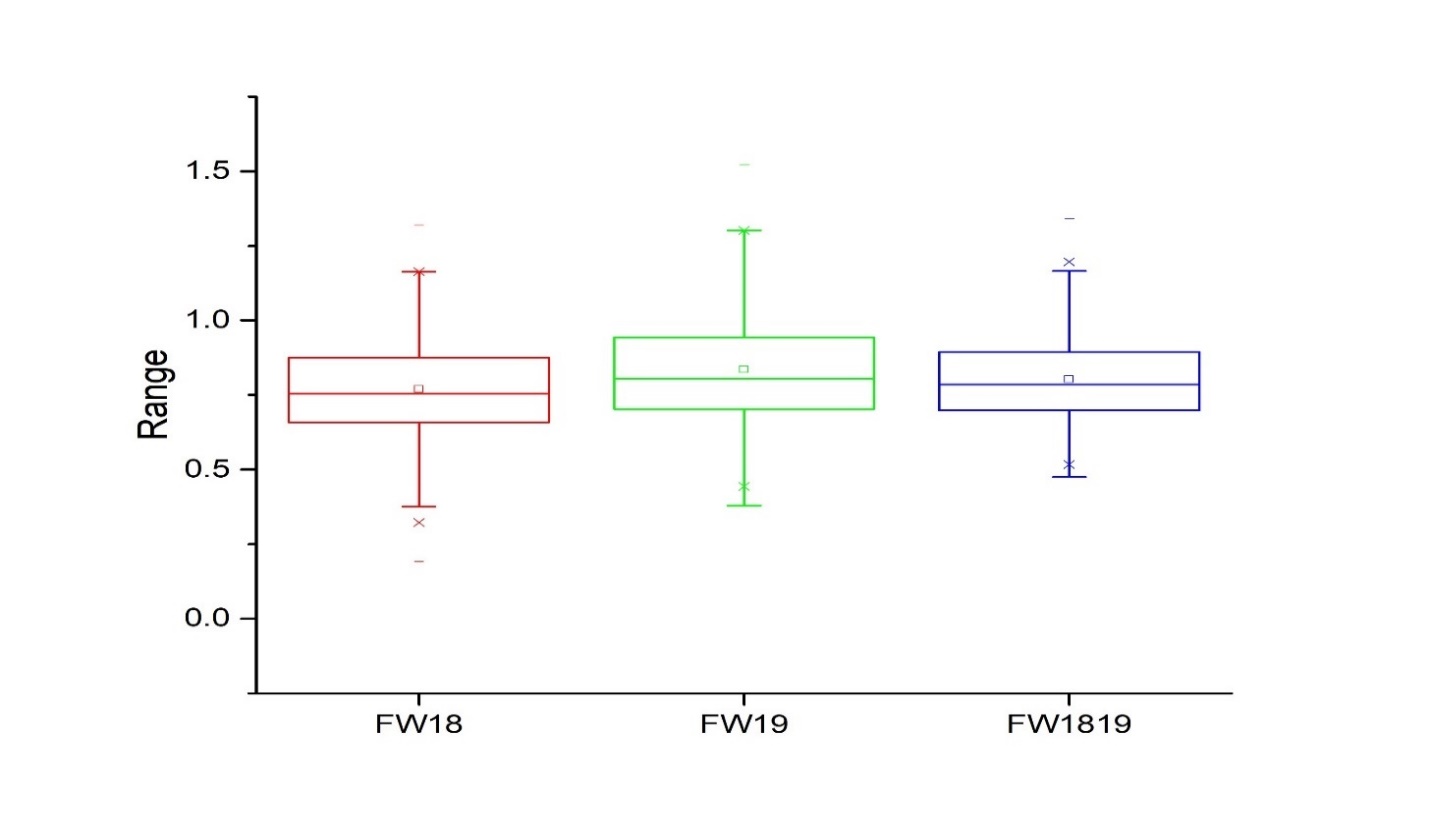
**

**S1b ***Each *x*-axis represents the trait under different years and *y*-axis shows the frequency of ranges corresponding with the value on *x*-axis. In each box chart, the lower and upper lines represent first and third quartiles, respectively, and the middle line shows the median. FW18, fruit weight (2018), FW19, fruit weight (2019), FW1819, fruit weight as extra year.

**
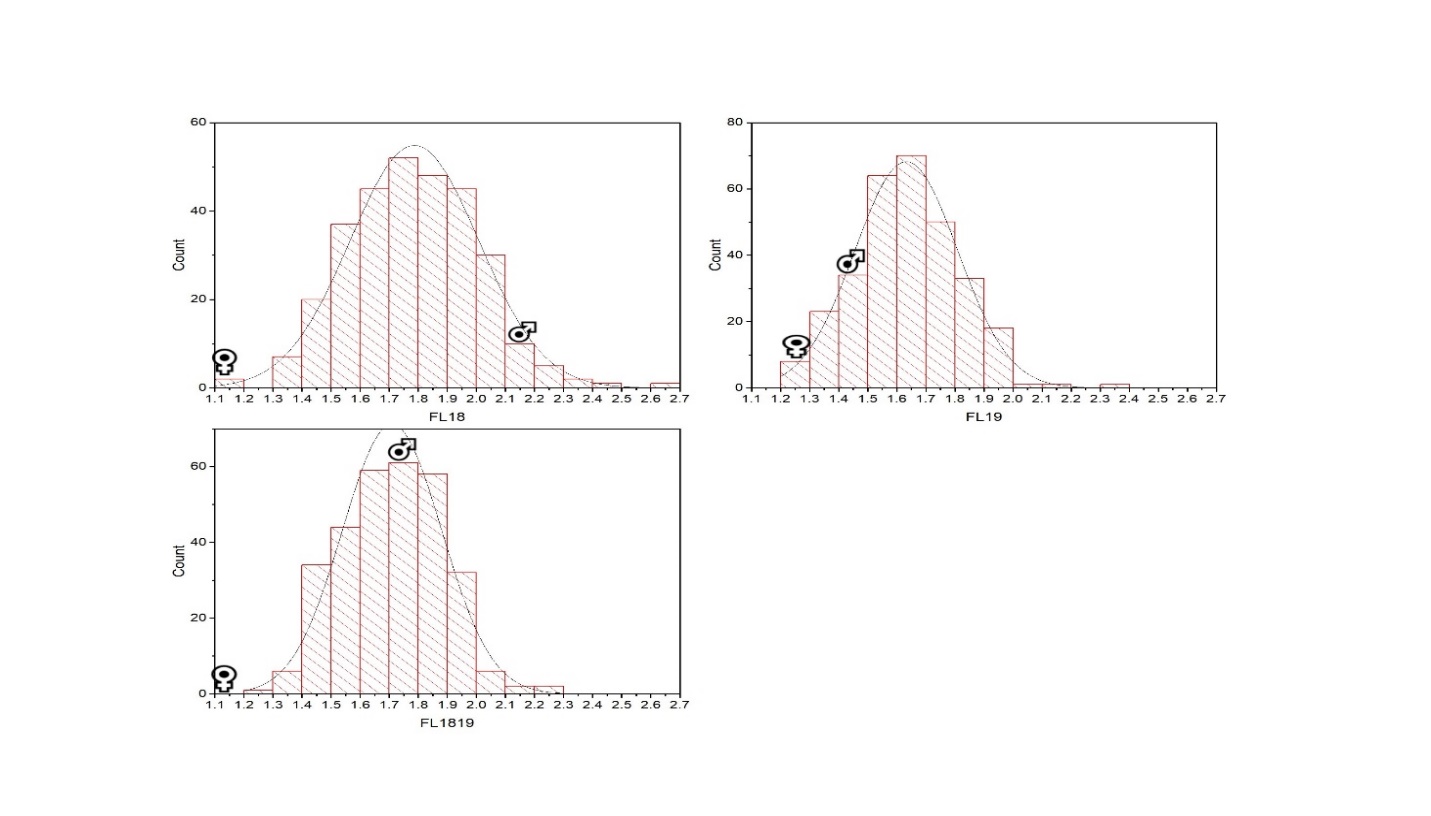
**

**S1c ***Each *x*-axis represents the value of the trait and *y*-axis shows the number of frequency corresponding with the value on *x*-axis. FL18, fruit length (2018), FL19, fruit length (2019), FL1819, fruit length as extra year; ♀, indicate female parent position on the histogram, ♂, male parent position on the histogram.

**
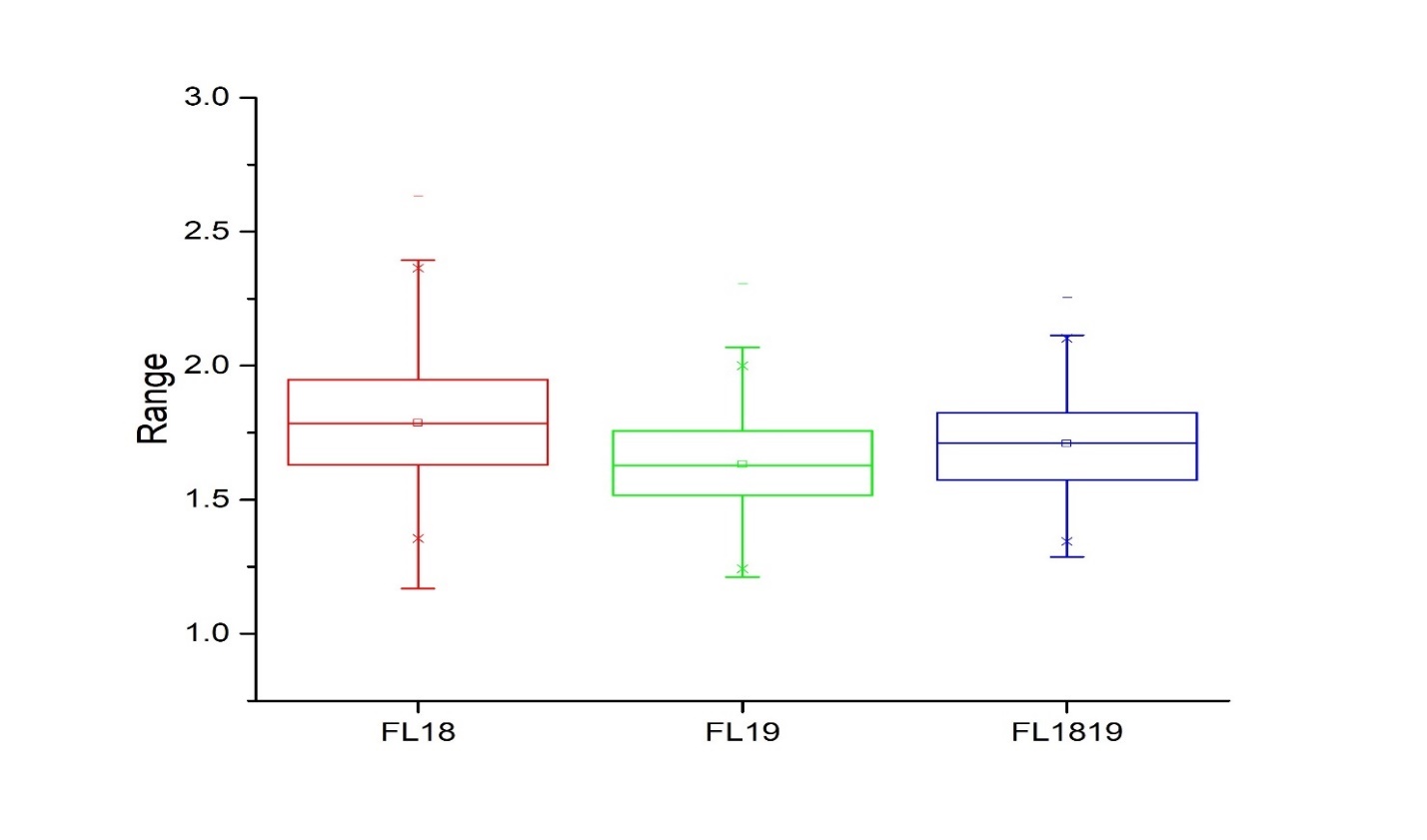
**

**S1d ***Each *x*-axis represents the trait under different years and *y*-axis shows the frequency of ranges corresponding with the value on *x*-axis. In each box chart, the lower and upper lines represent first and third quartiles, respectively, and the middle line shows the median. FL18, fruit length (2018), FL19, fruit length (2019), FL1819, fruit length as extra year.

**
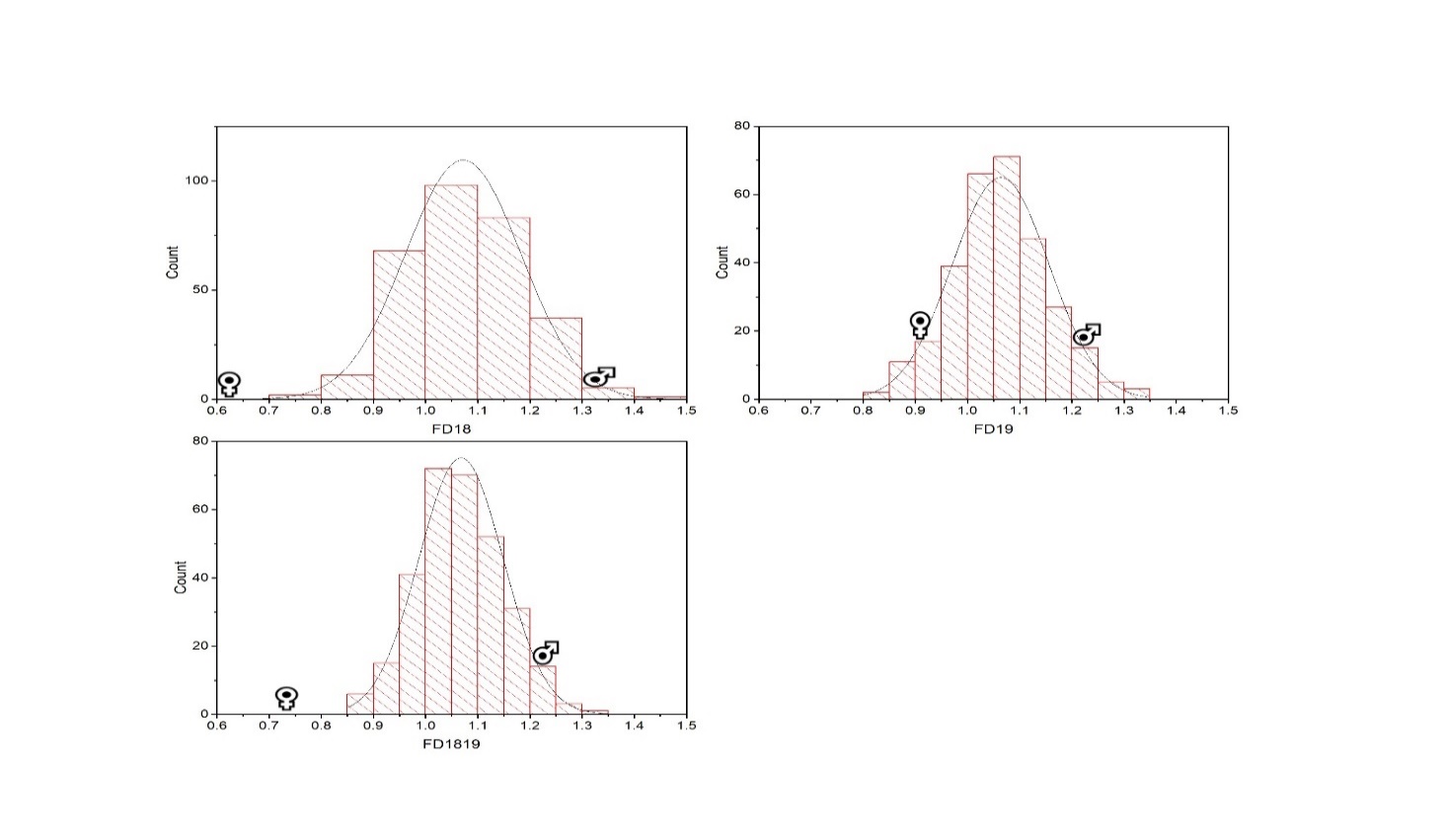
**

**S1e ***Each *x*-axis represents the value of the trait and *y*-axis shows the number of frequency corresponding with the value on *x*-axis. FD18, fruit diameter (2018), FD19, fruit diameter (2019), FD1819, fruit diameter as extra year; ♀, indicate female parent position on the histogram, ♂, male parent position on the histogram.

**
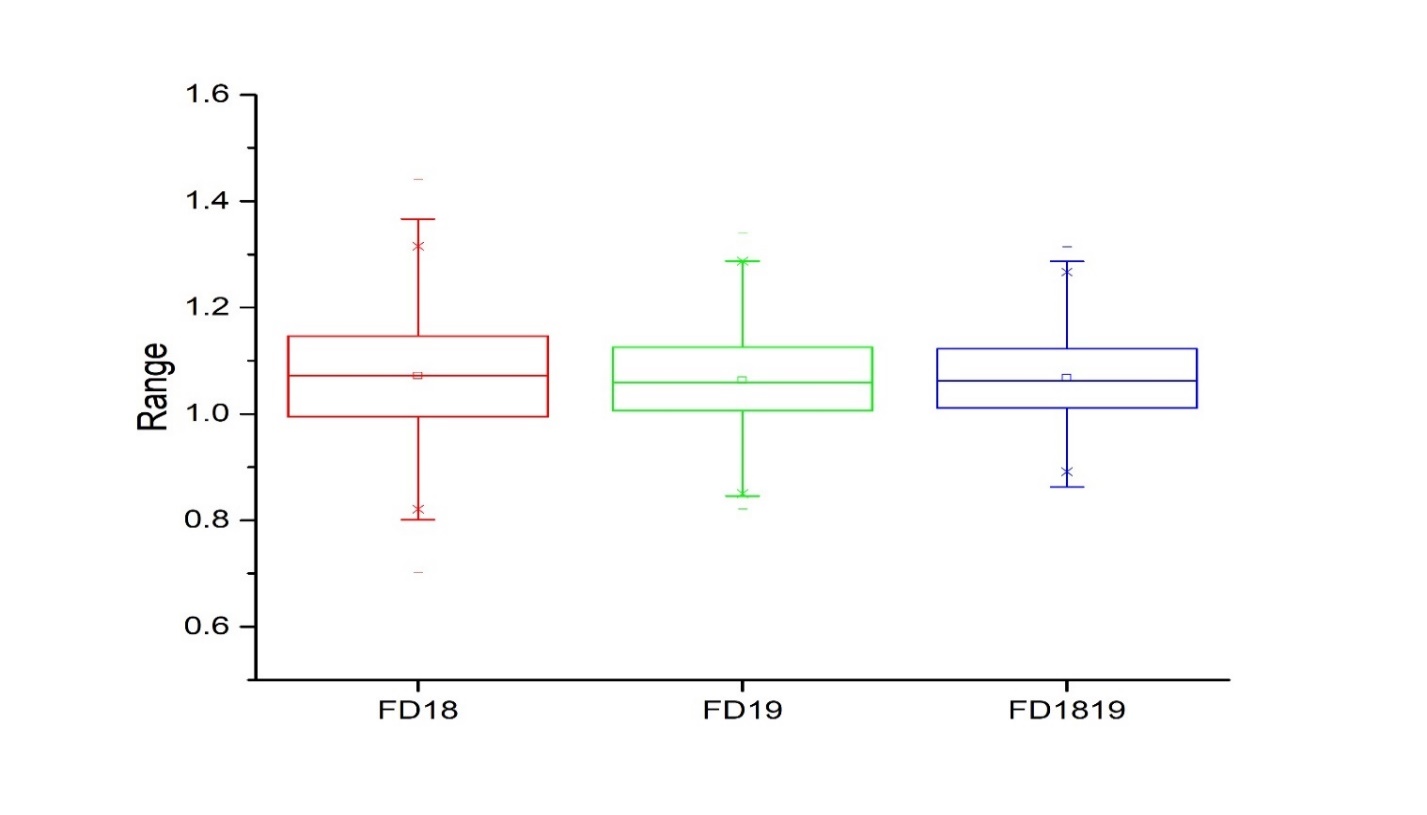
**

**S1f ***Each *x*-axis represents the trait under different years and *y*-axis shows the frequency of ranges corresponding with the value on *x*-axis. In each box chart, the lower and upper lines represent first and third quartiles, respectively, and the middle line shows the median. FD18, fruit diameter (2018), FD19, fruit diameter (2019), FD1819, fruit diameter as extra year.

**
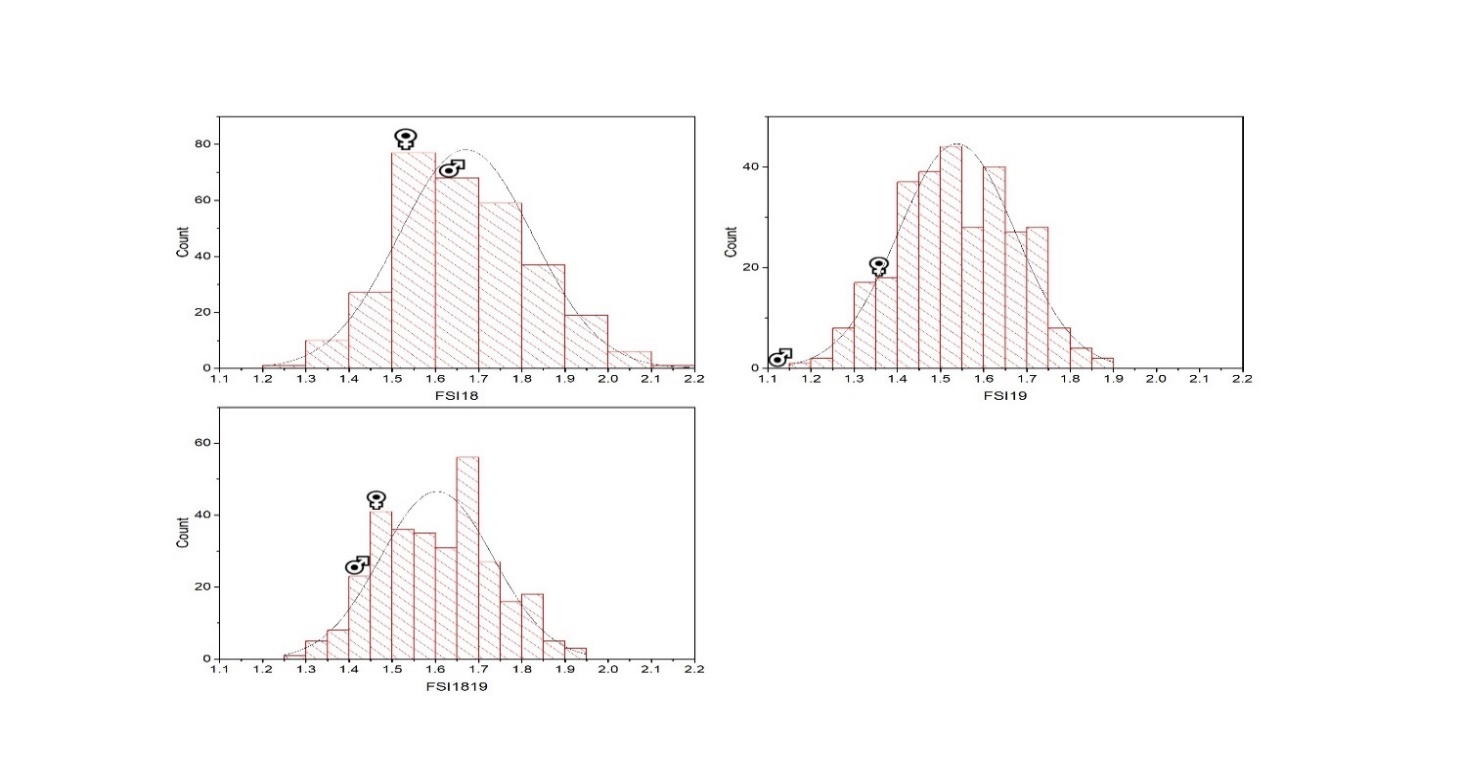
**

**S1g ***Each *x*-axis represents the value of the trait and *y*-axis shows the number of frequency corresponding with the value on *x*-axis. FSI18, fruit shape index (2018), FSI19, fruit shape index (2019), FSI1819, fruit shape index as extra year; ♀, indicate female parent position on the histogram, ♂, male parent position on the histogram.

**
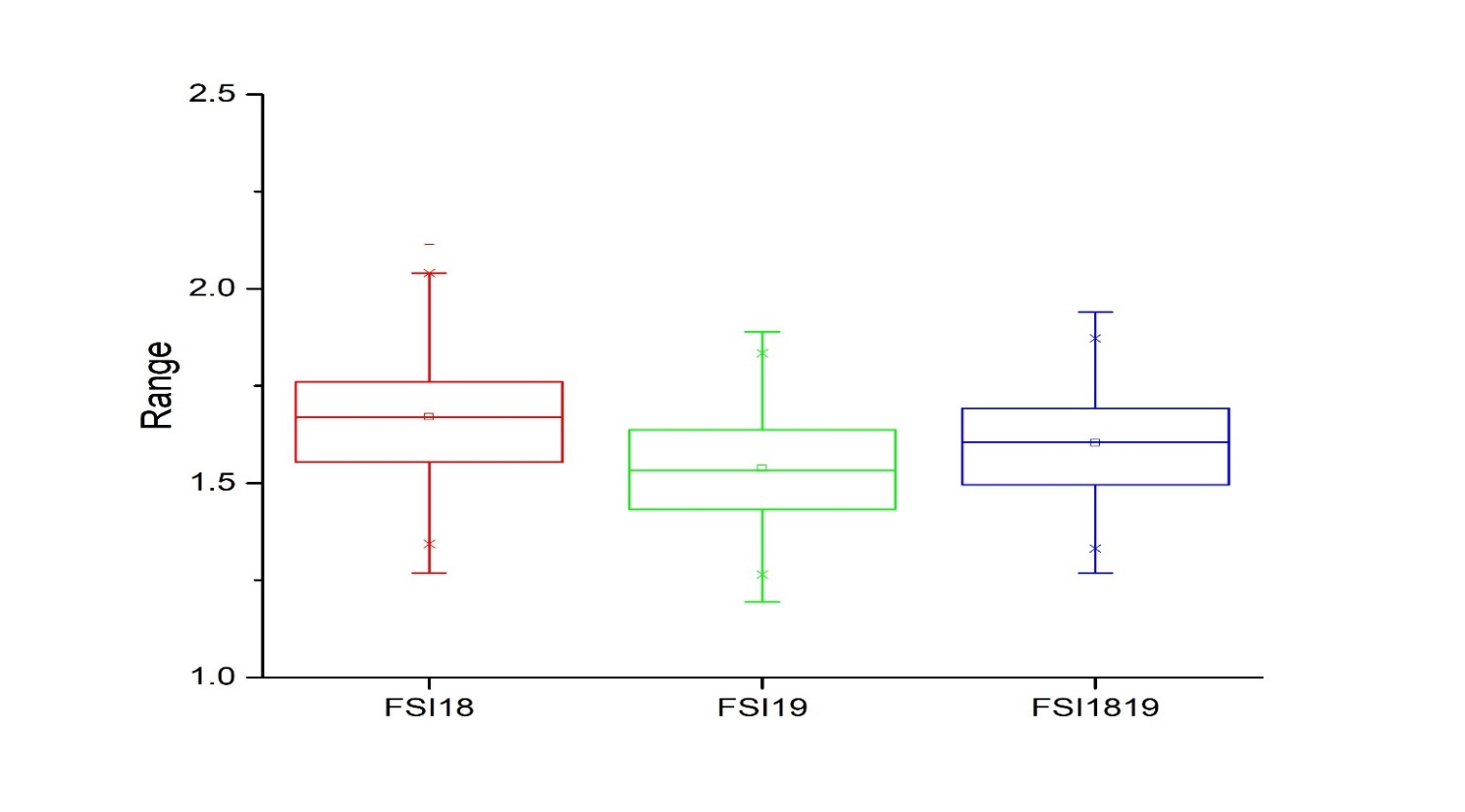
**

**S1h ***Each *x*-axis represents the trait under different years and *y*-axis shows the frequency of ranges corresponding with the value on *x*-axis. In each box chart, the lower and upper lines represent first and third quartiles, respectively, and the middle line shows the median. FSI18, fruit shape index (2018), FSI19, fruit shape index (2019), FSI1819, fruit shape index as extra year.

**
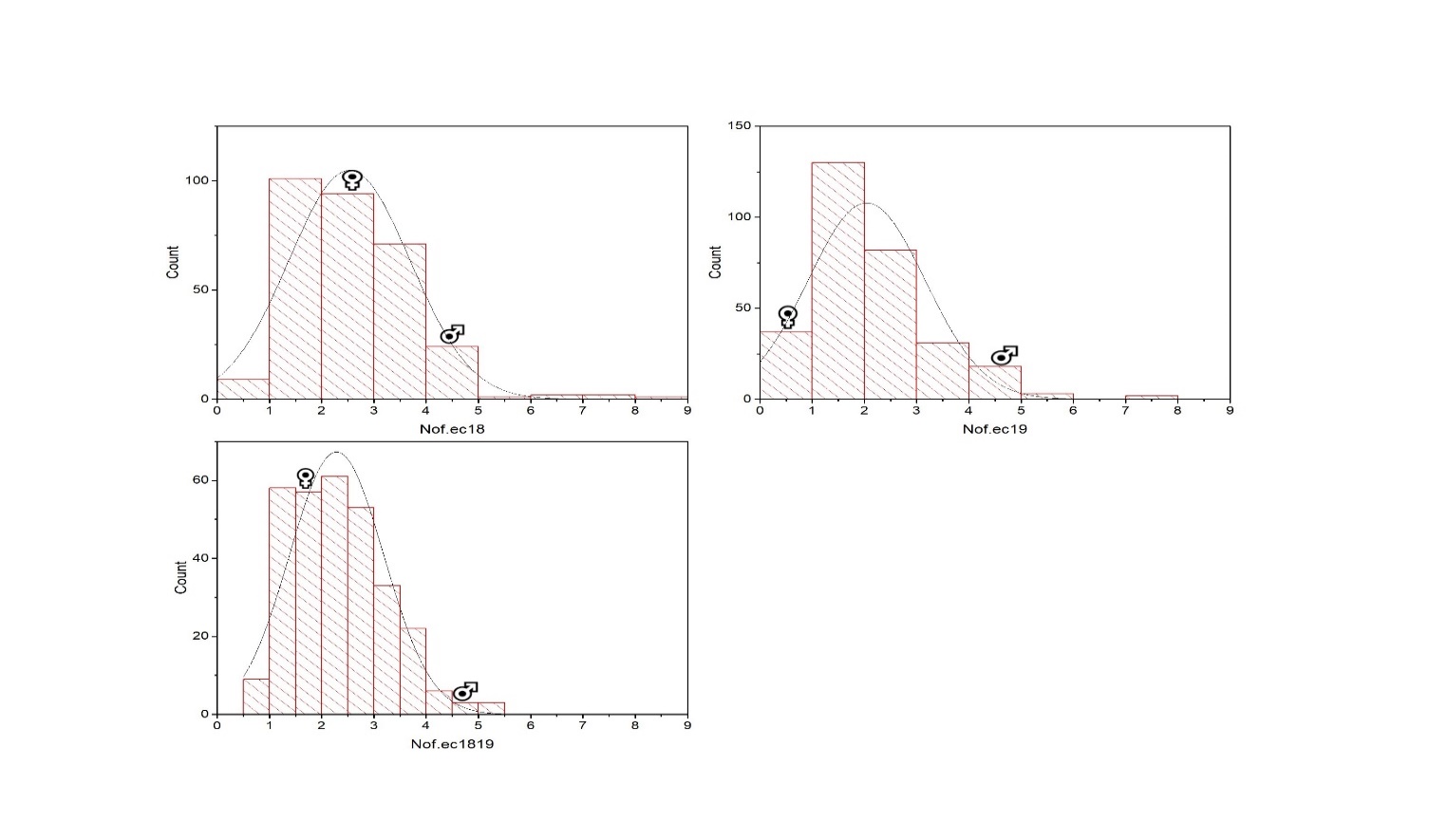
**

**S1i ***Each *x*-axis represents the value of the trait and *y*-axis shows the number of frequency corresponding with the value on *x*-axis. Nof/ec18, number of fruits per end cluster (2018), Nof/ec19, number of fruits per end cluster (2019), Nof/ec1819, number of fruits per end cluster as extra year; ♀, indicate female parent position on the histogram, ♂, male parent position on the histogram.

**
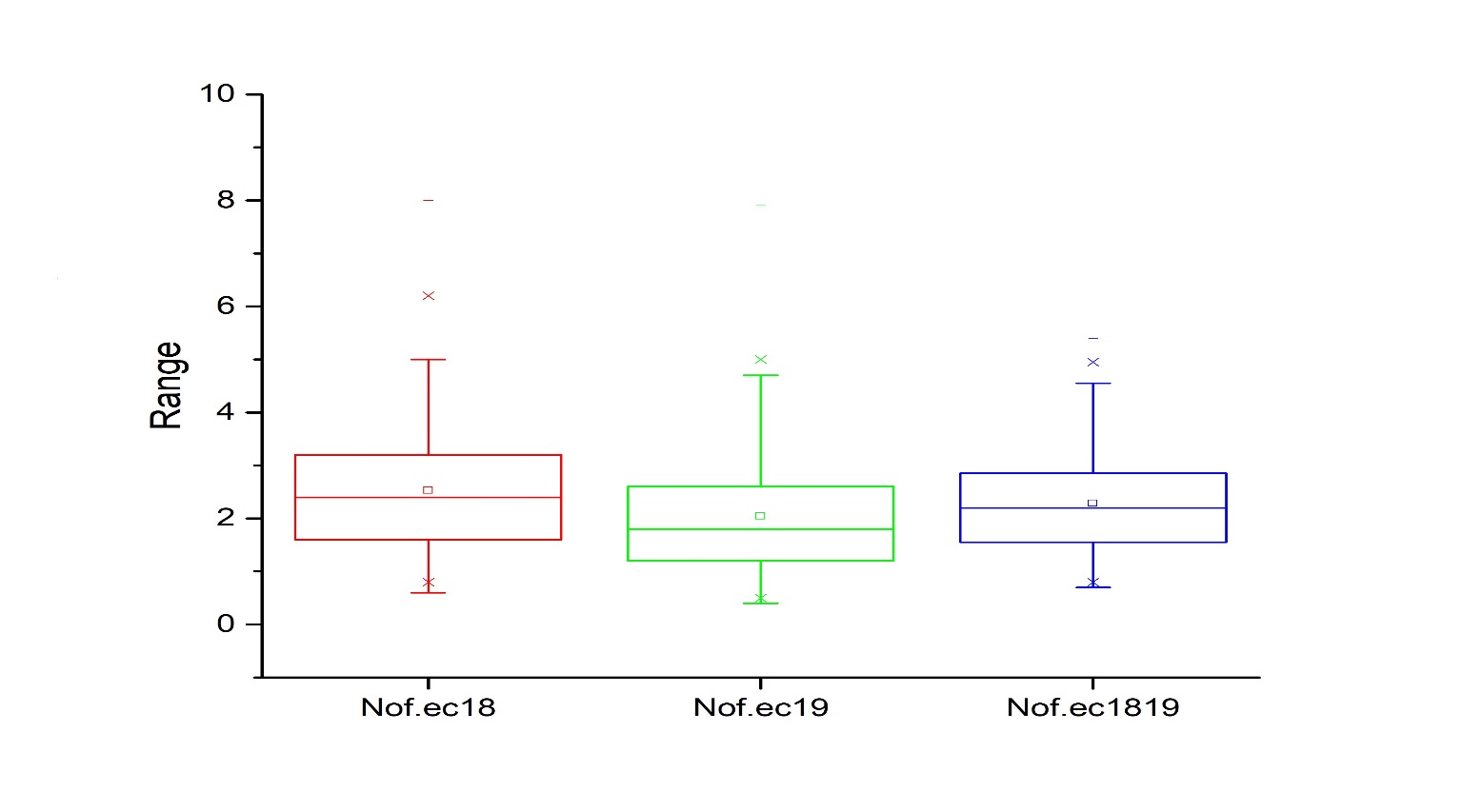
**

**S1j ***Each *x*-axis represents the trait under different years and *y*-axis shows the frequency of ranges corresponding with the value on *x*-axis. In each box chart, the lower and upper lines represent first and third quartiles, respectively, and the middle line shows the median. Nof/ec18, number of fruits per end cluster (2018), Nof/ec19, number of fruits per end cluster (2019), Nof/ec1819, number of fruits per end cluster as extra year.

**
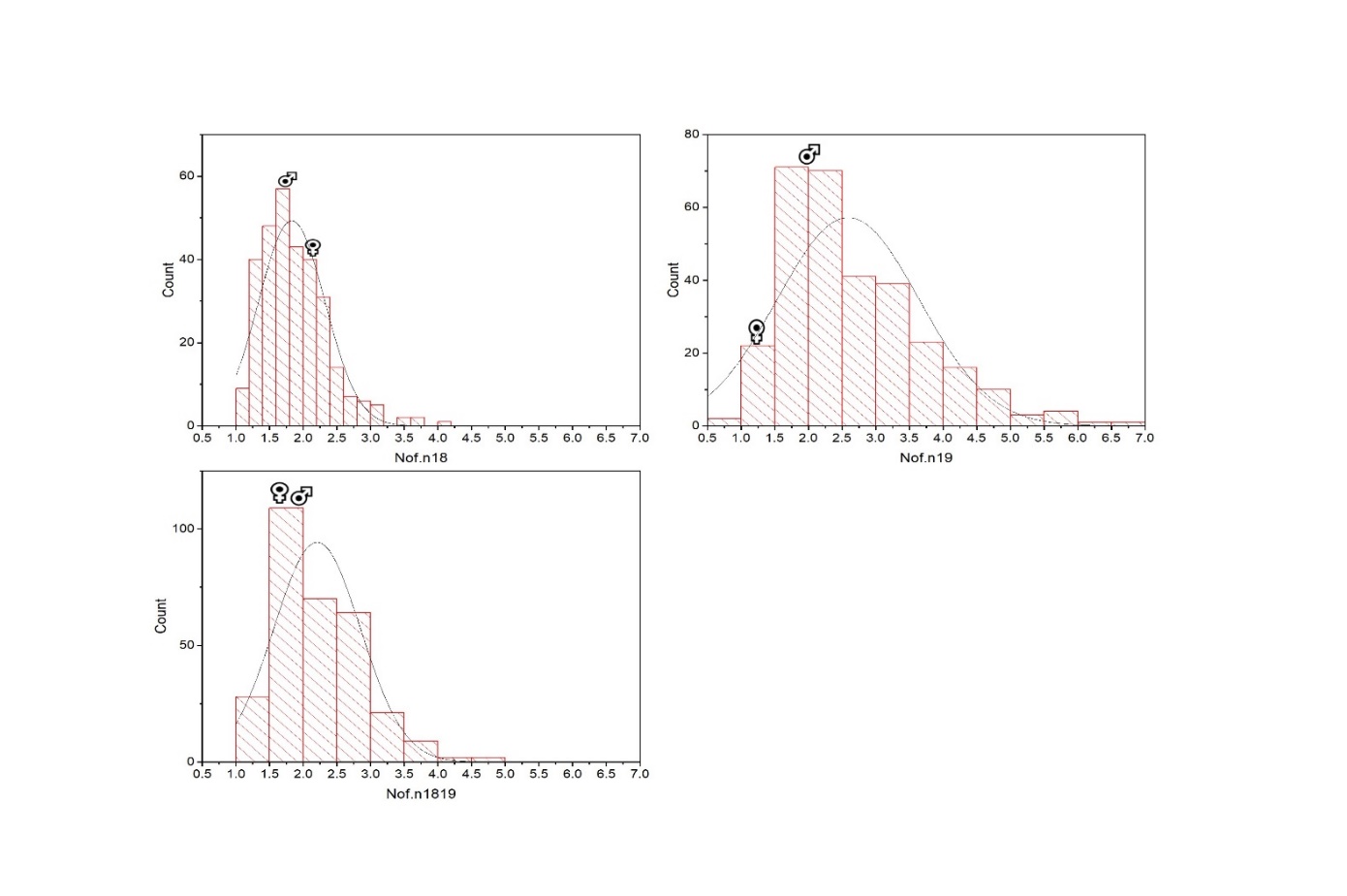
**

**S1k ***Each *x*-axis represents the value of the trait and *y*-axis shows the number of frequency corresponding with the value on *x*-axis. Nof/n18, number of fruits per node (2018), Nof/n19, number of fruits per node (2019), Nof/n1819, number of fruits per node as extra year; ♀, indicate female parent position on the histogram, ♂, male parent position on the histogram.

**
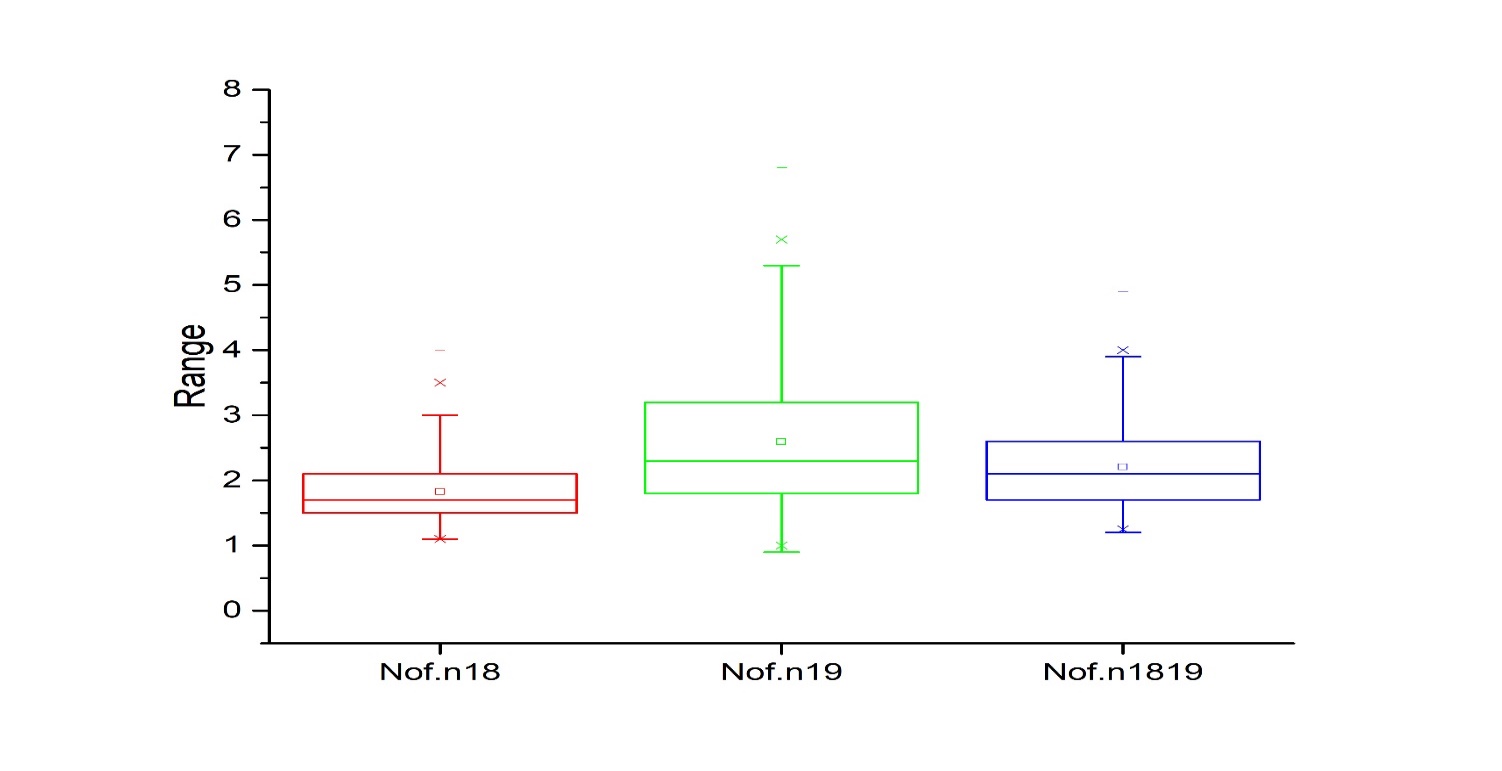
**

**S1l ***Each *x*-axis represents the trait under different years and *y*-axis shows the frequency of ranges corresponding with the value on *x*-axis. In each box chart, the lower and upper lines represent first and third quartiles, respectively, and the middle line shows the median. Nof/n18, number of fruits per node (2018), Nof/n19, number of fruits per node (2019), Nof/n1819, number of fruits per node as extra year.

**
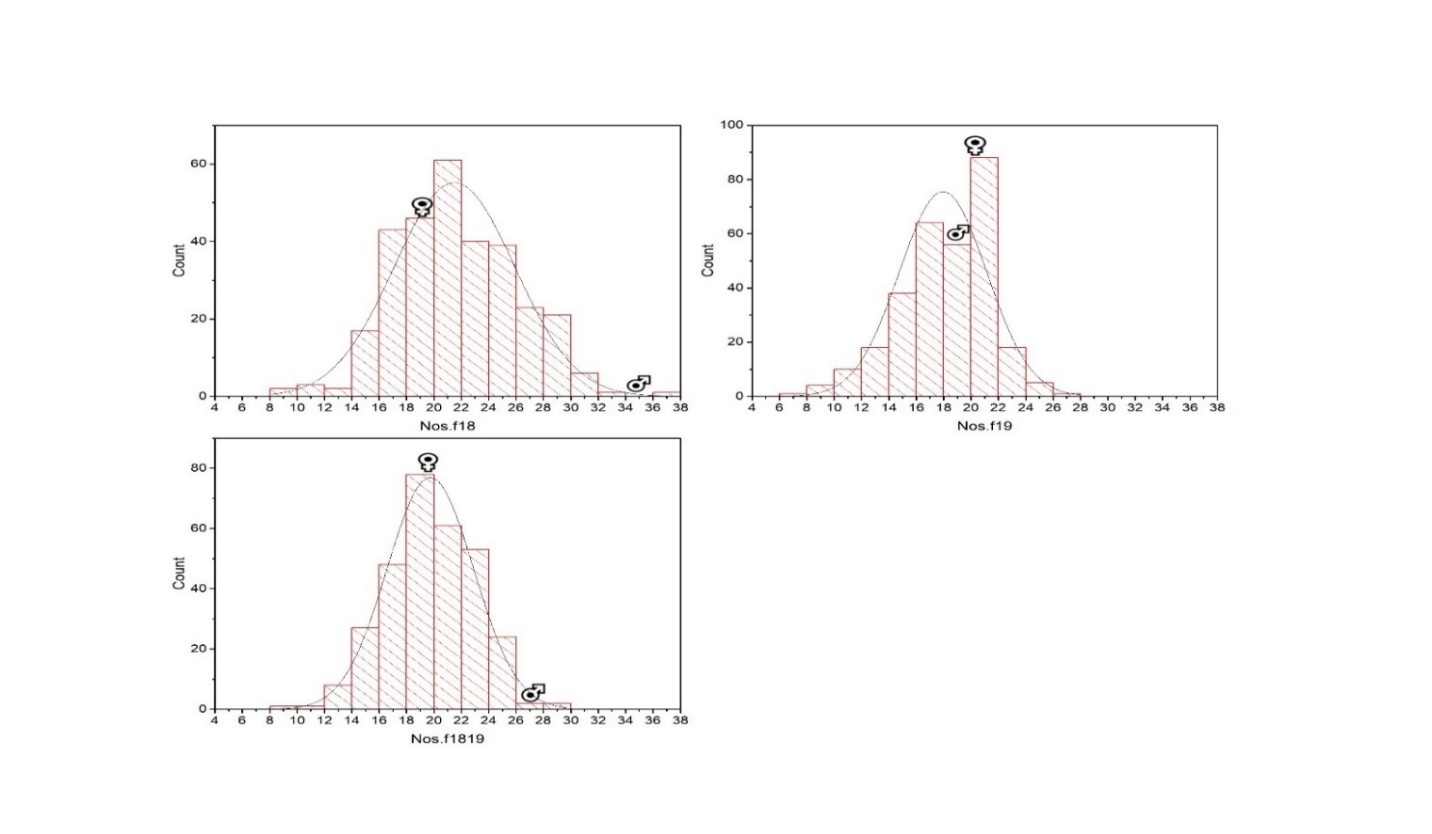
**

**S1m ***Each *x*-axis represents the value of the trait and *y*-axis shows the number of frequency corresponding with the value on *x*-axis. Nos/f18, number of seeds per fruit (2018), Nos/f19, number of seeds per fruit (2019), Nos/f1819, number of seeds per fruit as extra year; ♀, indicate female parent position on the histogram, ♂, male parent position on the histogram.

**
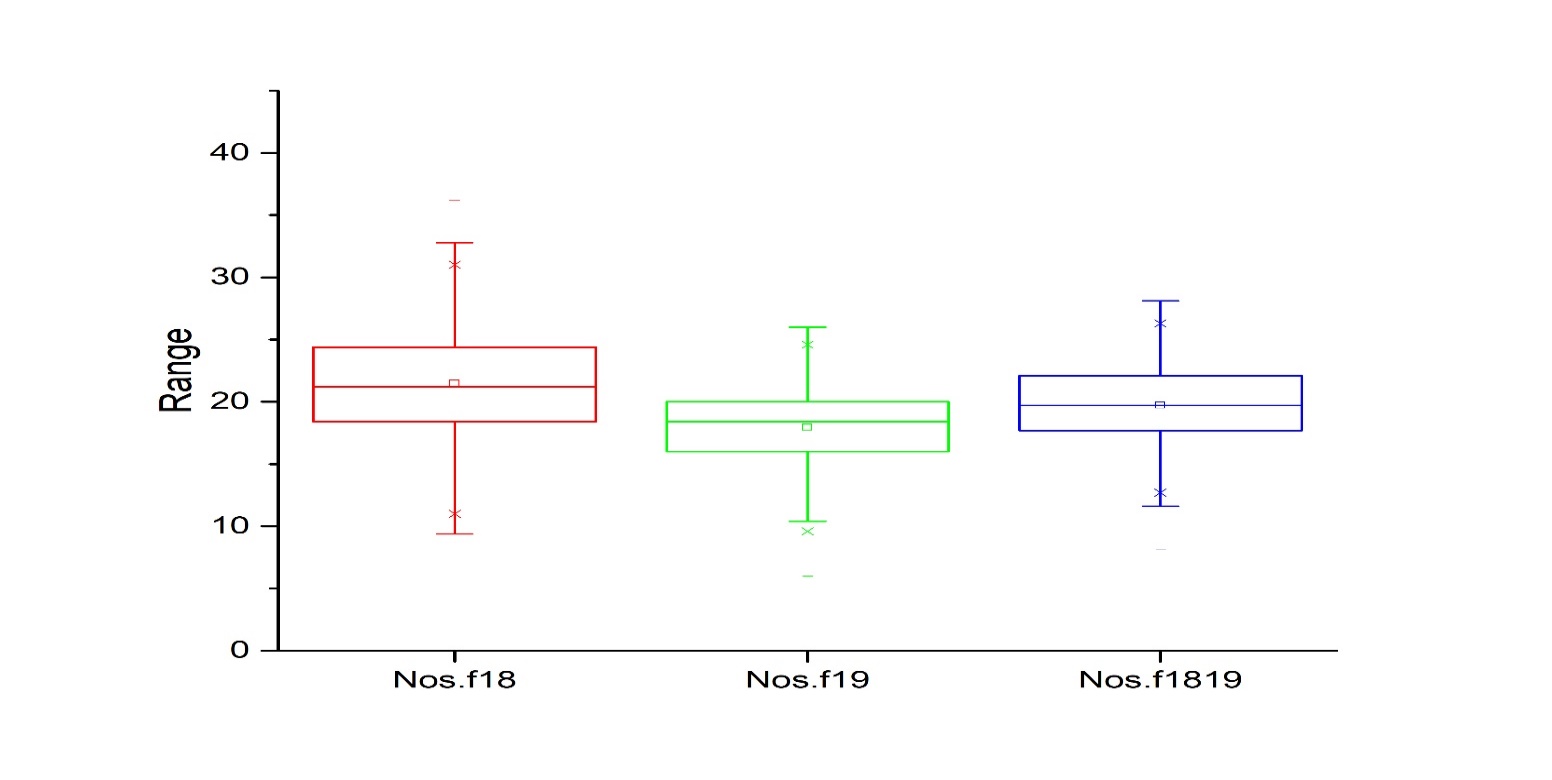
**

**S1n ***Each *x*-axis represents the trait under different years and *y*-axis shows the frequency of ranges corresponding with the value on *x*-axis. In each box chart, the lower and upper lines represent first and third quartiles, respectively, and the middle line shows the median. Nof/n18, number of seeds per fruit (2018), Nos/f19, number of seeds per fruit (2019), Nos/f1819, number of seeds per fruit as extra year.

**
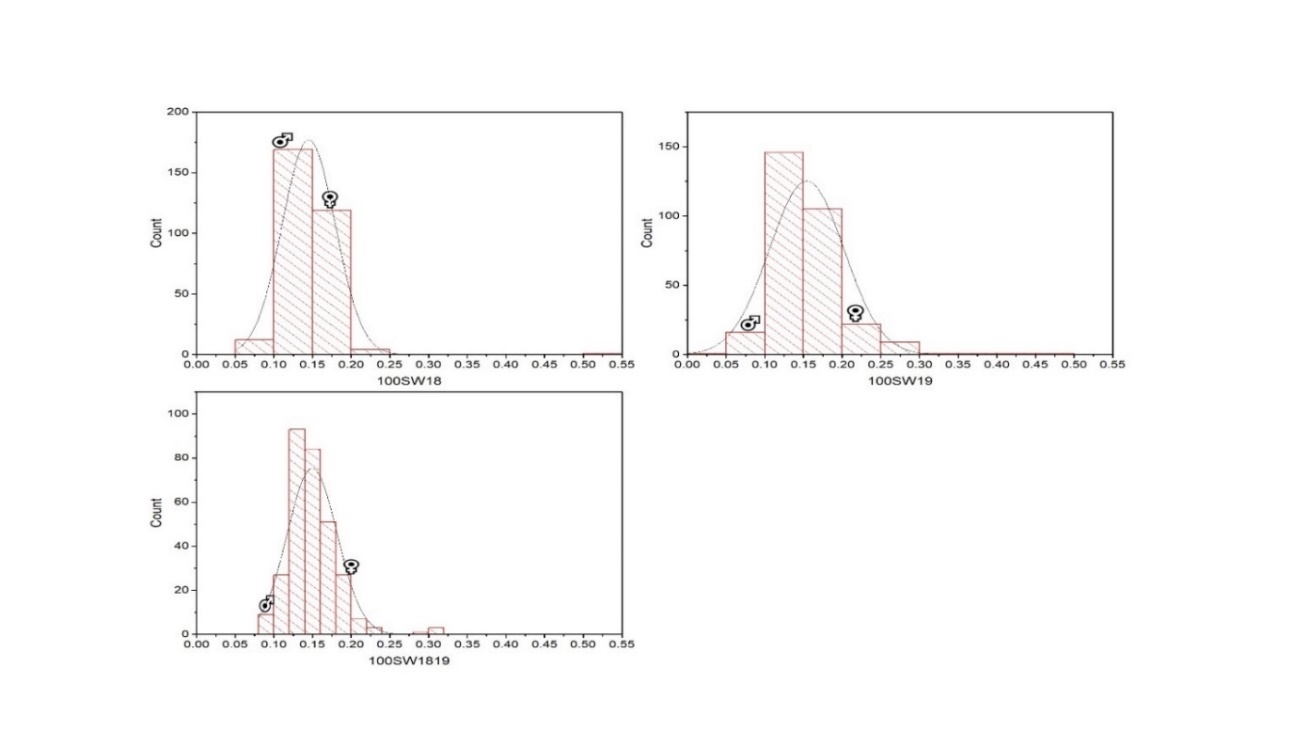
**

**S1o ***Each *x*-axis represents the value of the trait and *y*-axis shows the number of frequency corresponding with the value on *x*-axis. 100SW18, 100 seeds weight (2018), 100SW19, 100 seeds weight (2019), 100SW1819, 100 seeds weight as extra year; ♀, indicate female parent position on the histogram, ♂, male parent position on the histogram.

**
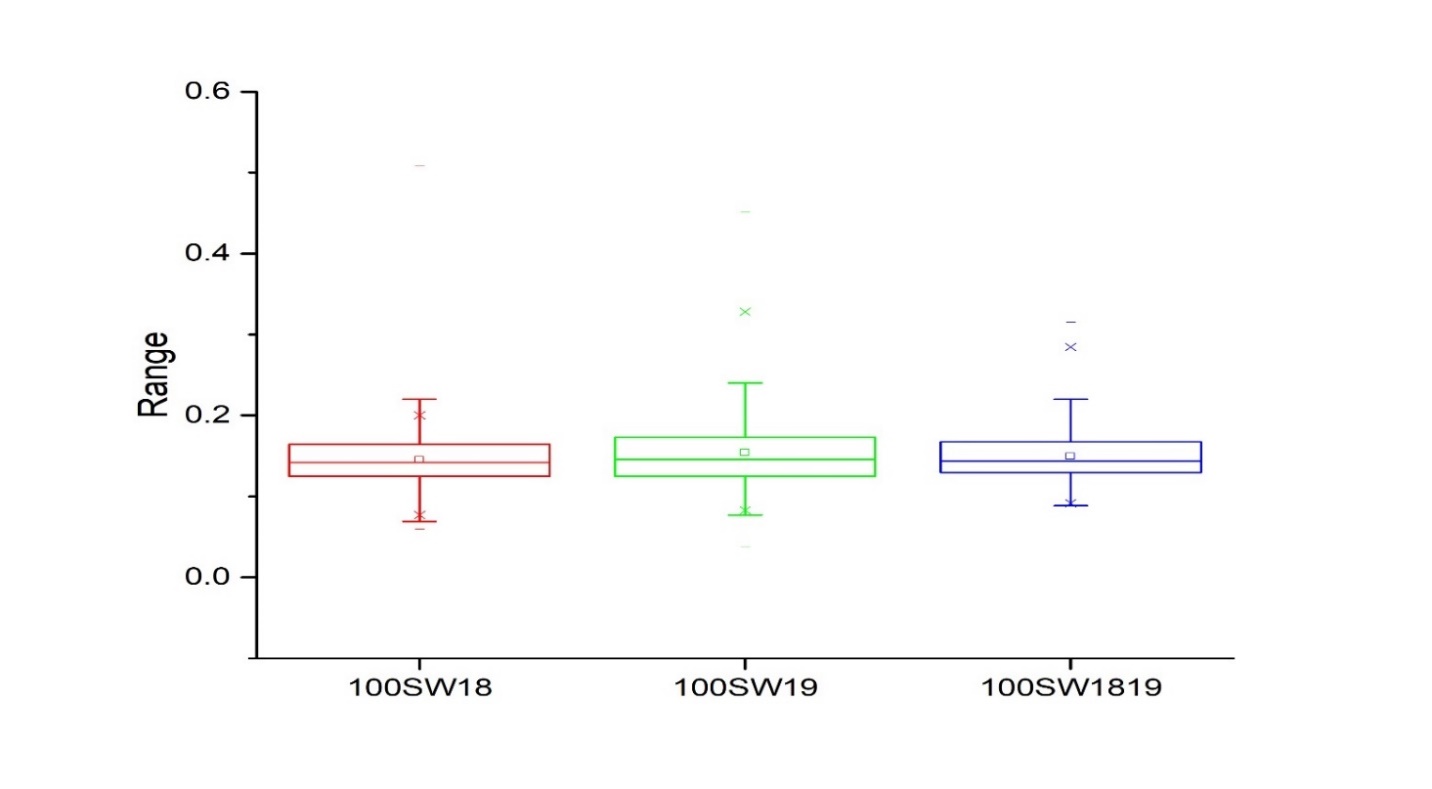
**

**S1p ***Each *x*-axis represents the trait under different years and *y*-axis shows the frequency of ranges corresponding with the value on *x*-axis. In each box chart, the lower and upper lines represent first and third quartiles, respectively, and the middle line shows the median. 100SW18, 100 seeds weight (2018), 100SW19, 100 seeds weight (2019), 100SW1819, 100 seeds weight as extra year.

**
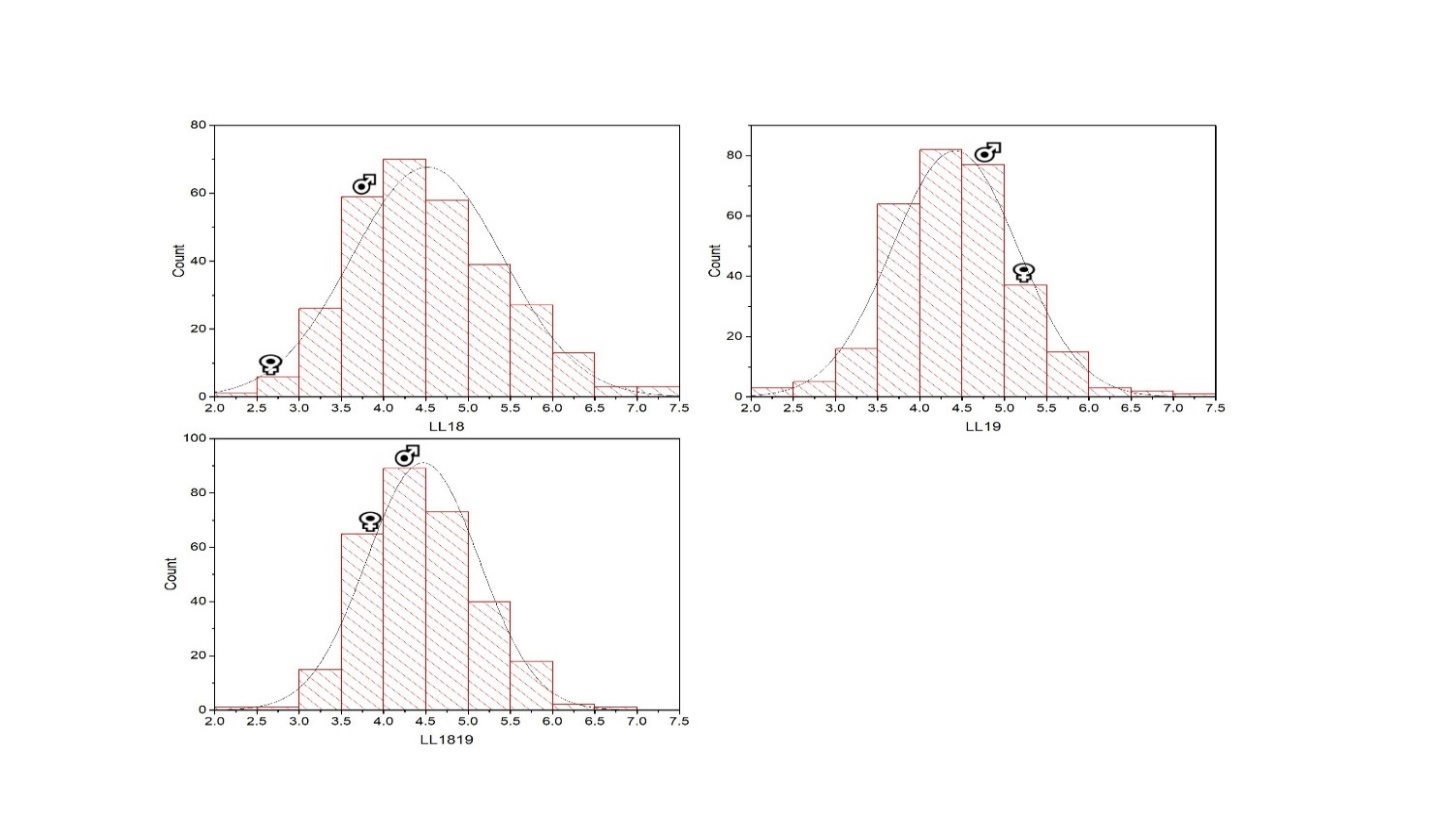
**

**S1q ***Each *x*-axis represents the value of the trait and *y*-axis shows the number of frequency corresponding with the value on *x*-axis. LL18, leaf length (2018), LL19, leaf length (2019), LL1819, leaf length as extra year; ♀, indicate female parent position on the histogram, ♂, male parent position on the histogram.

**
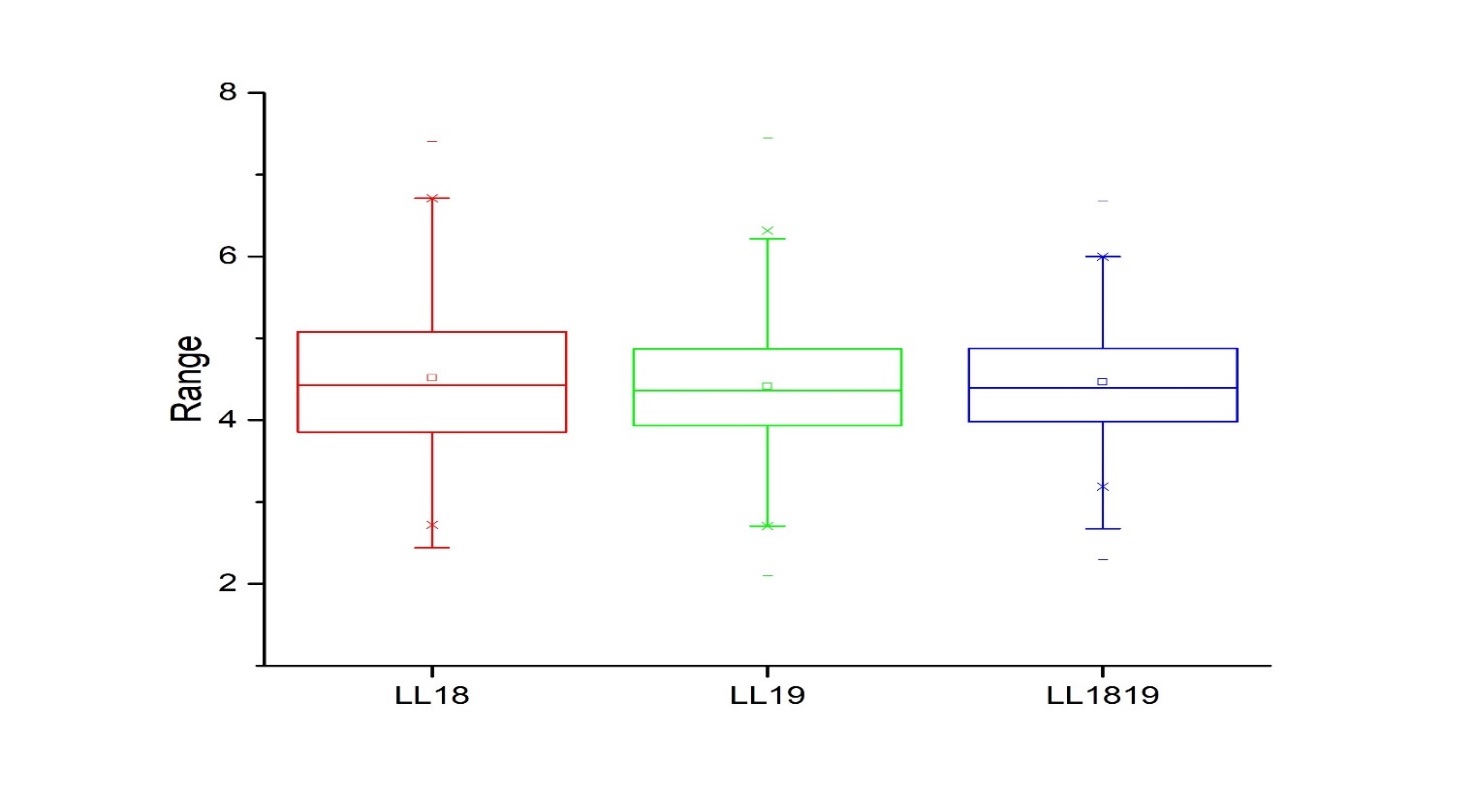
**

**S1p ***Each *x*-axis represents the trait under different years and *y*-axis shows the frequency of ranges corresponding with the value on *x*-axis. In each box chart, the lower and upper lines represent first and third quartiles, respectively, and the middle line shows the median. LL18, leaf length (2018), LL19, leaf length (2019), LL1819, leaf length as extra year.

**
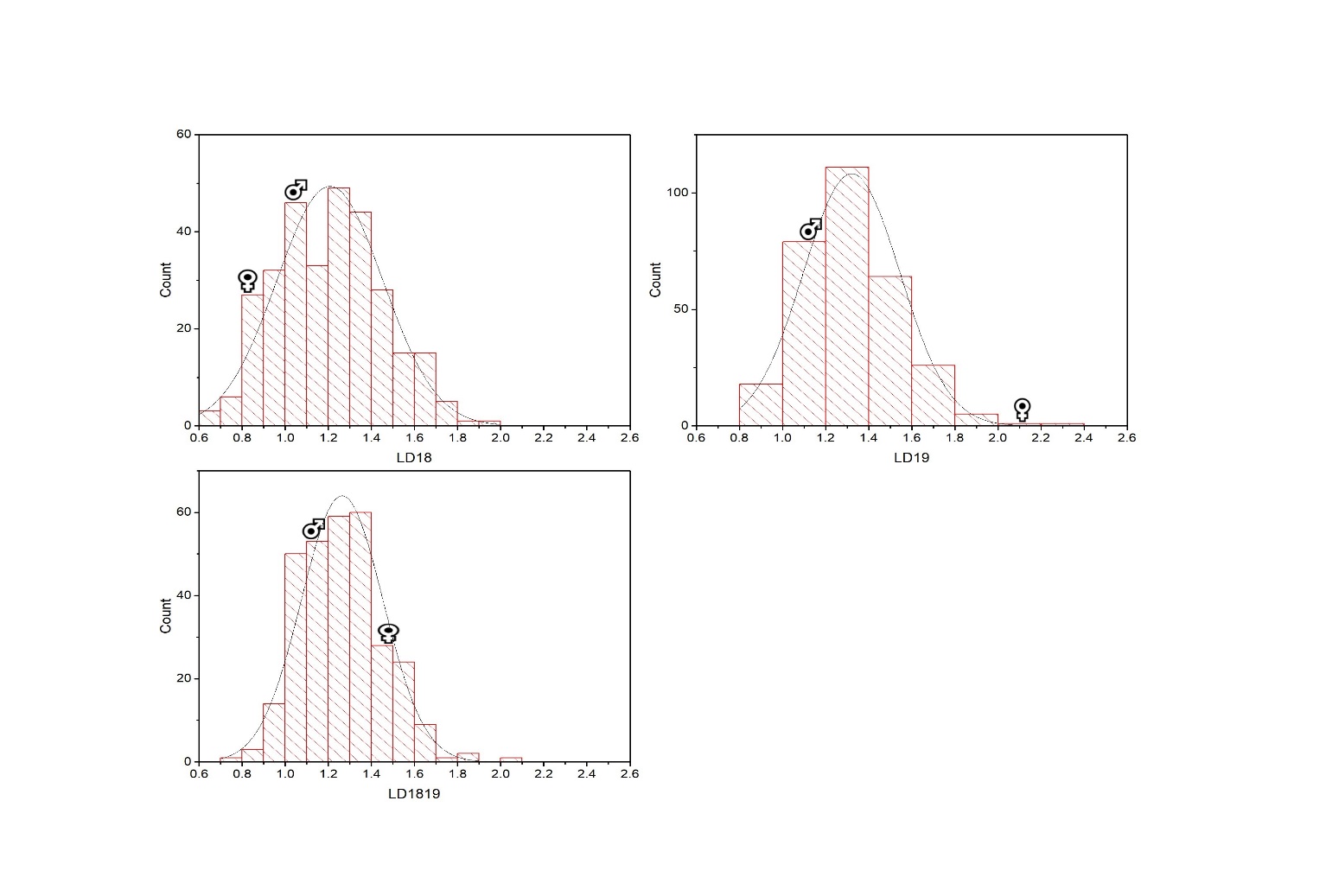
**

**S1r ***Each *x*-axis represents the value of the trait and *y*-axis shows the number of frequency corresponding with the value on *x*-axis. LD18, leaf diameter (2018), LD19, leaf diameter (2019), LD1819, leaf diameter as extra year; ♀, indicate female parent position on the histogram, ♂, male parent position on the histogram.

**
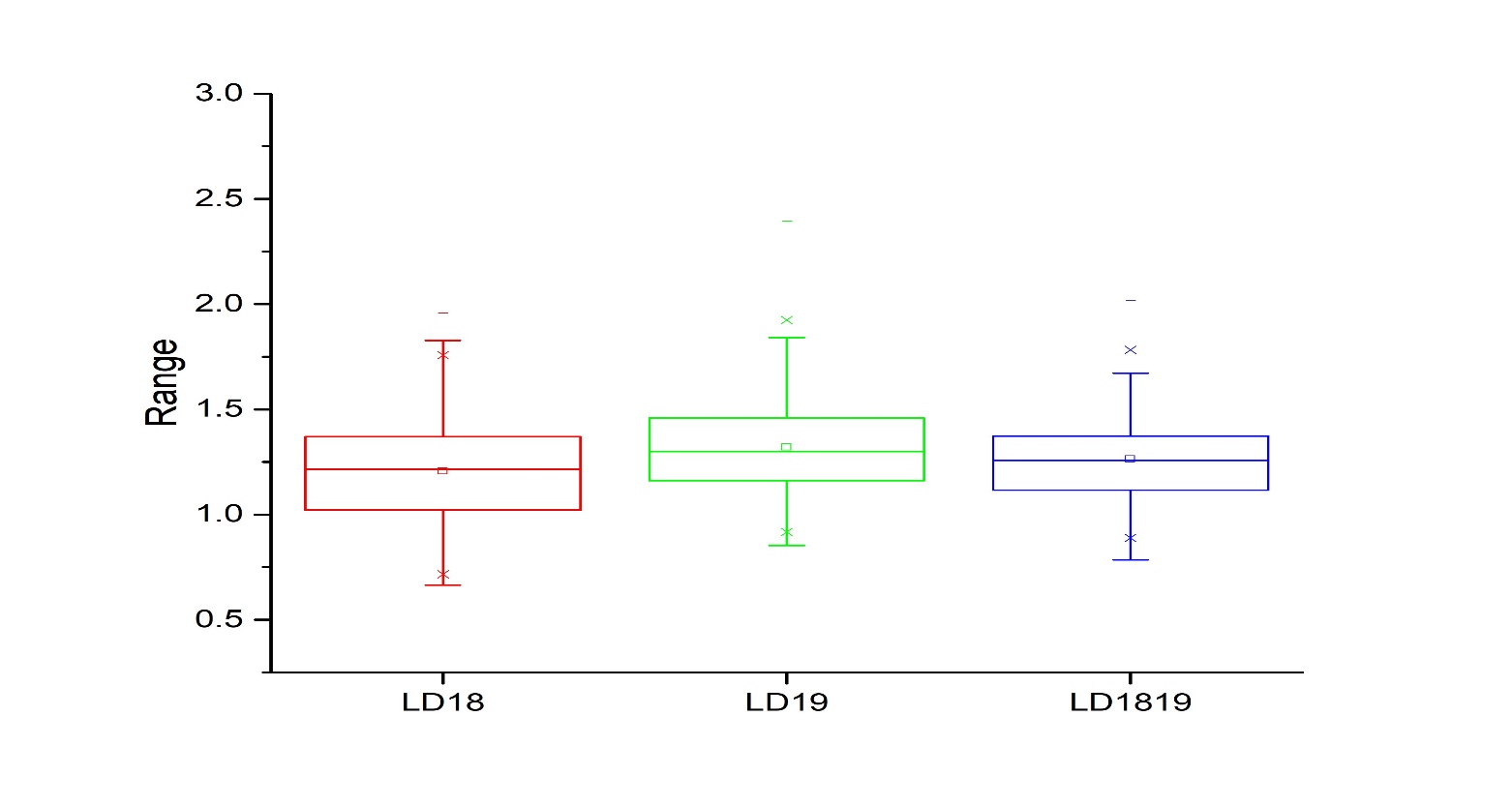
**

**S1t ***Each *x*-axis represents the trait under different years and *y*-axis shows the frequency of ranges corresponding with the value on *x*-axis. In each box chart, the lower and upper lines represent first and third quartiles, respectively, and the middle line shows the median. LL18, leaf diameter (2018), LD19, leaf diameter (2019), LD1819, leaf diameter as extra year.

**
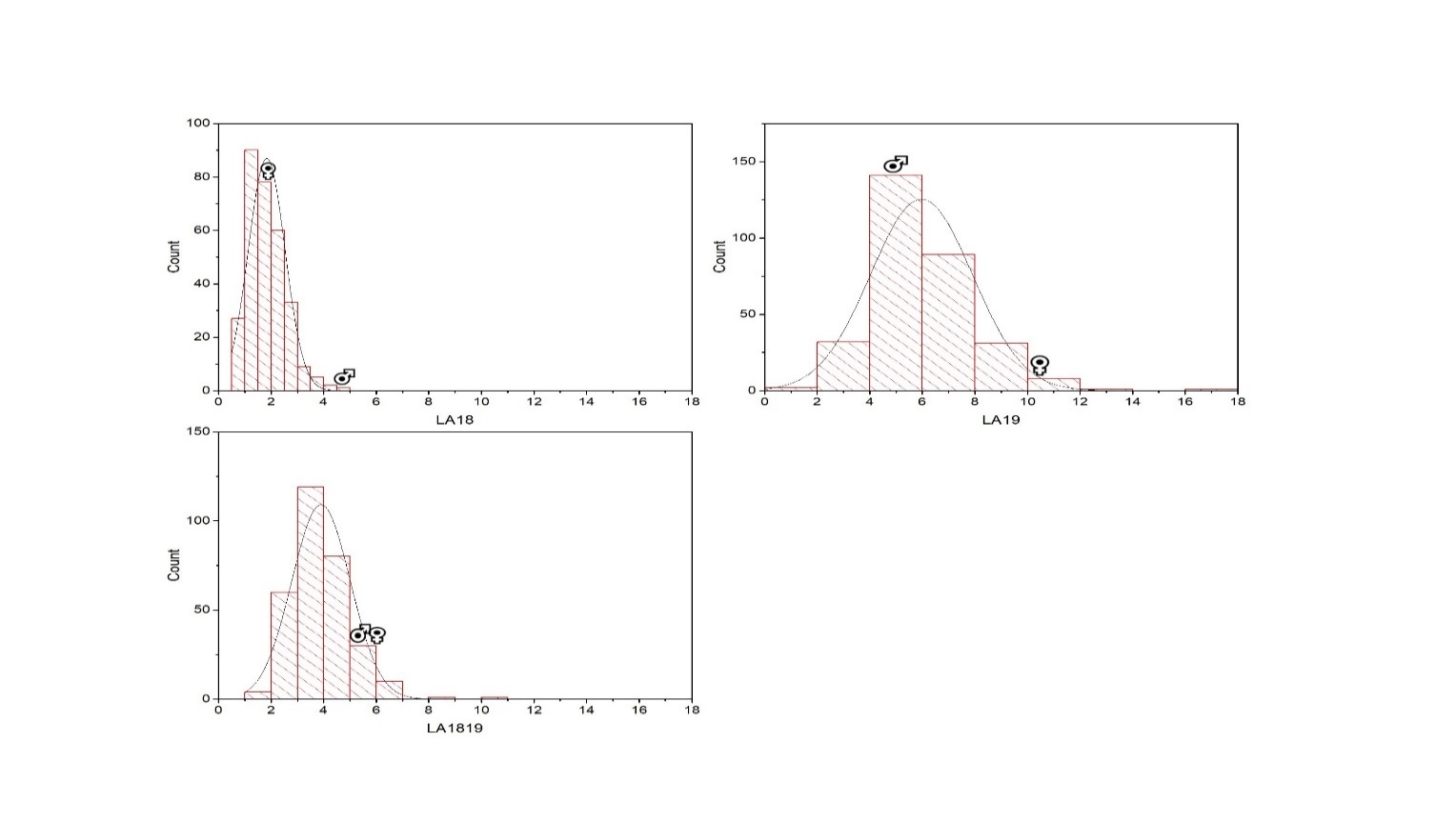
**

**S1w ***Each *x*-axis represents the value of the trait and *y*-axis shows the number of frequency corresponding with the value on *x*-axis. LA18, leaf area (2018), LA19, leaf area (2019), LA1819, leaf area as extra year; ♀, indicate female parent position on the histogram, ♂, male parent position on the histogram.

**
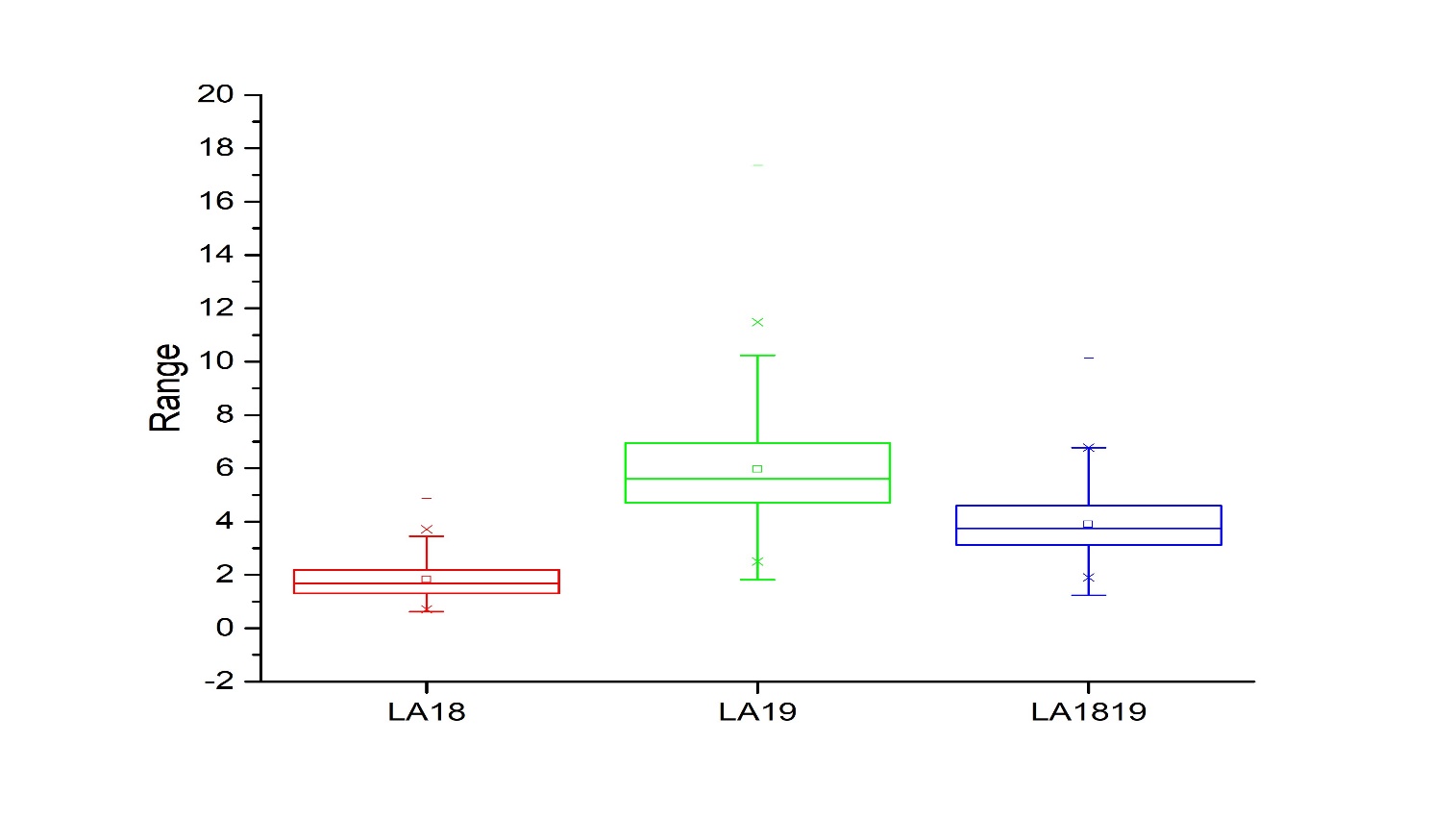
**

**S1x ***Each *x*-axis represents the trait under different years and *y*-axis shows the frequency of ranges corresponding with the value on *x*-axis. In each box chart, the lower and upper lines represent first and third quartiles, respectively, and the middle line shows the median. LA18, leaf area (2018), LA19, leaf area (2019), LA1819, leaf area as extra year.

**
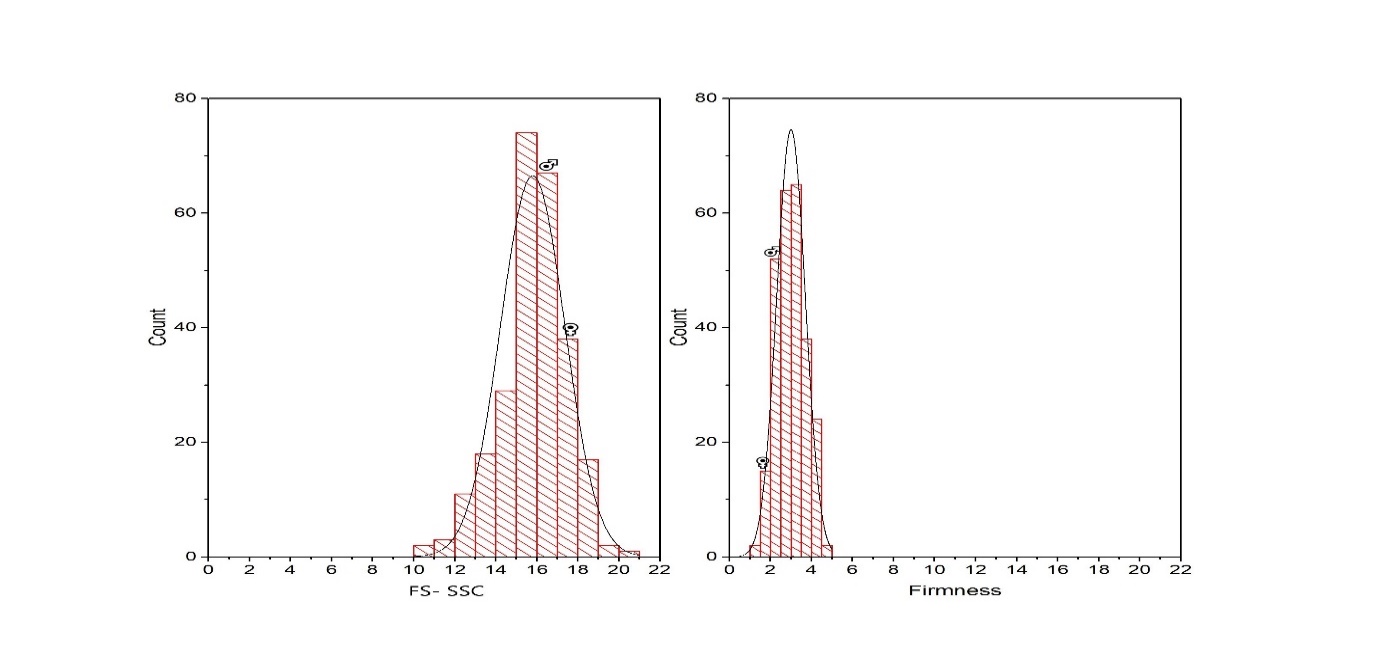
**

**S1y ***Each *x*-axis represents the value of the trait and *y*-axis shows the number of frequency corresponding with the value on *x*-axis. Left; FS-SSC, fruit sweetness- soluble solid contents (2019), Right; FF, fruit firmness (2019); ♀, indicate female parent position on the histogram, ♂, male parent position on the histogram.

**
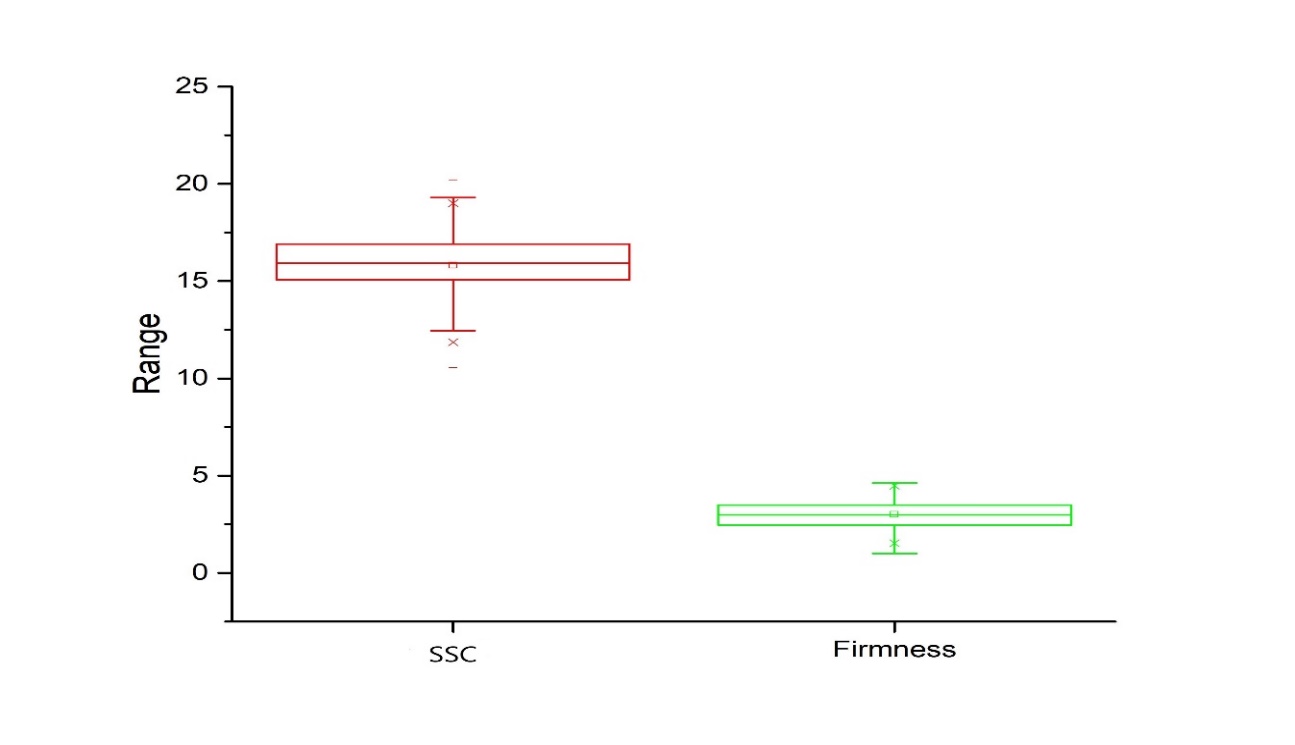
**

**S1z ***Each *x*-axis represents the trait under one individual year and *y*-axis shows the frequency of ranges corresponding with the value on *x*-axis. In each box chart, the lower and upper lines represent first and third quartiles, respectively, and the middle line shows the median. FS-SSC, fruit sweetness- soulbe solid contents (2019), FF, fruit firmness (2019).


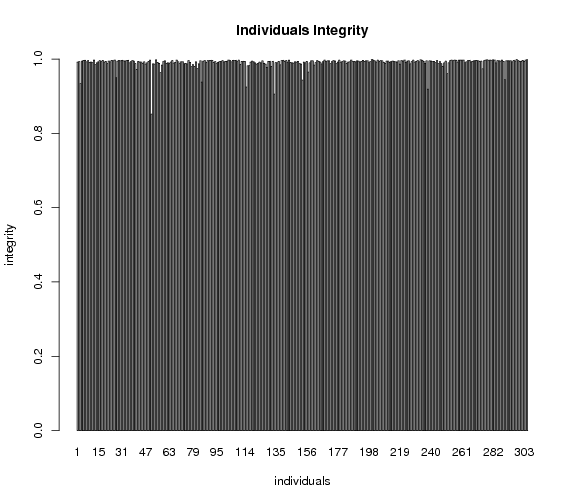


**Figure S2**
