# Supplementary material for: Identification of fruit size associated quantitative trait loci featuring SLAF based high-density linkage map of goji berry (Lycium spp.)
Source: BMC Plant Biol. 2020 Oct 15;20:474. doi: 10.1186/s12870-020-02567-1 (PMC7565837; doi:10.1186/s12870-020-02567-1)
Supplement: Supplementary file 2 — Additional file 2: Figure S3(a-l). Haplotype mapping of 305 F1 individuals based on 12 linkage groups of integrated maps. *In each map, horizontal line stands a marker and column shows a chromosome in a sample. The first column of the map shows a paternal chromosome and the second column as maternal chromosome, and individuals are separated by blank columns. Green color indicates first allele from the parent, blue as the second allele from the parent, white color shows not judged event, and grey indicates a missing event. The position where color changes in the same column display reorganization events. Figure S4(a-l). Heatmaps of 305 F1 individuals based on 12 linkage groups of integrated maps. *Each row and column represent marker arranged in the orderliness of the map. Every small square show recombination rate between two markers. The color change trend from yellow to red to purple display reorganization rate from small to large. Yellow color indicates closer marker recombination rate, whereas closer the color from yellow to purple farther becomes the recombination rate. [file 12870_2020_2567_MOESM2_ESM.docx]

**3a. LG1**

**
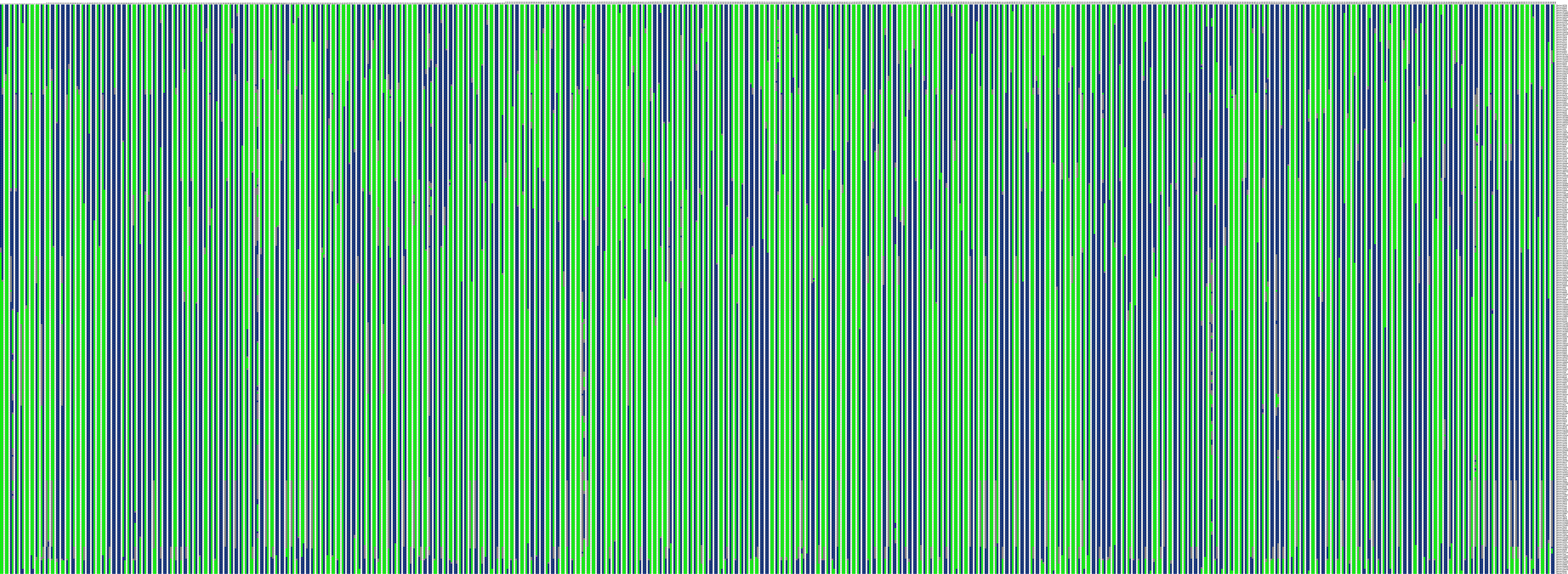
**

**3b. LG2**

**
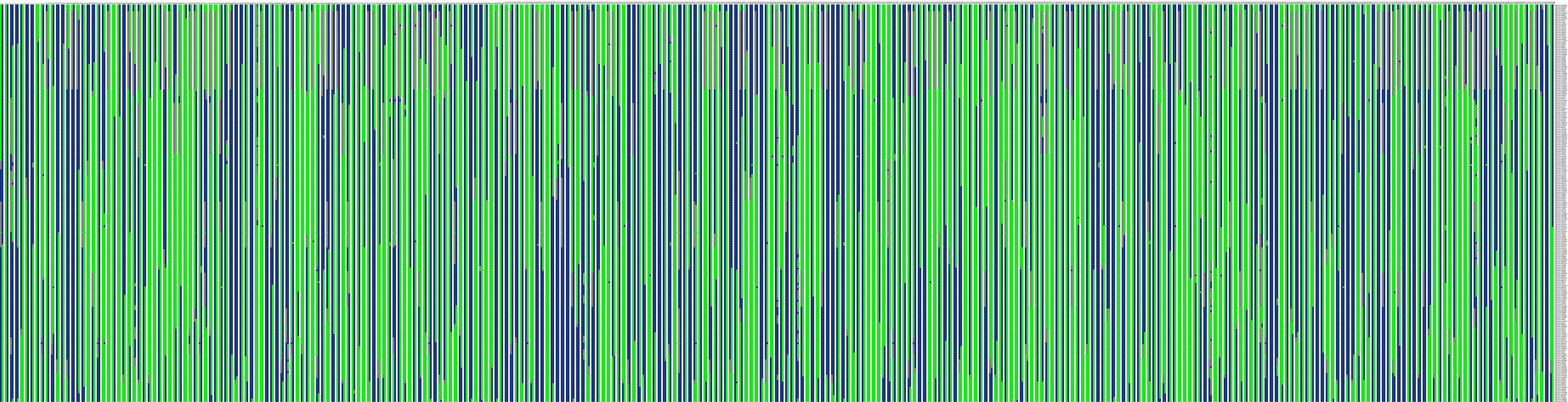
**

**
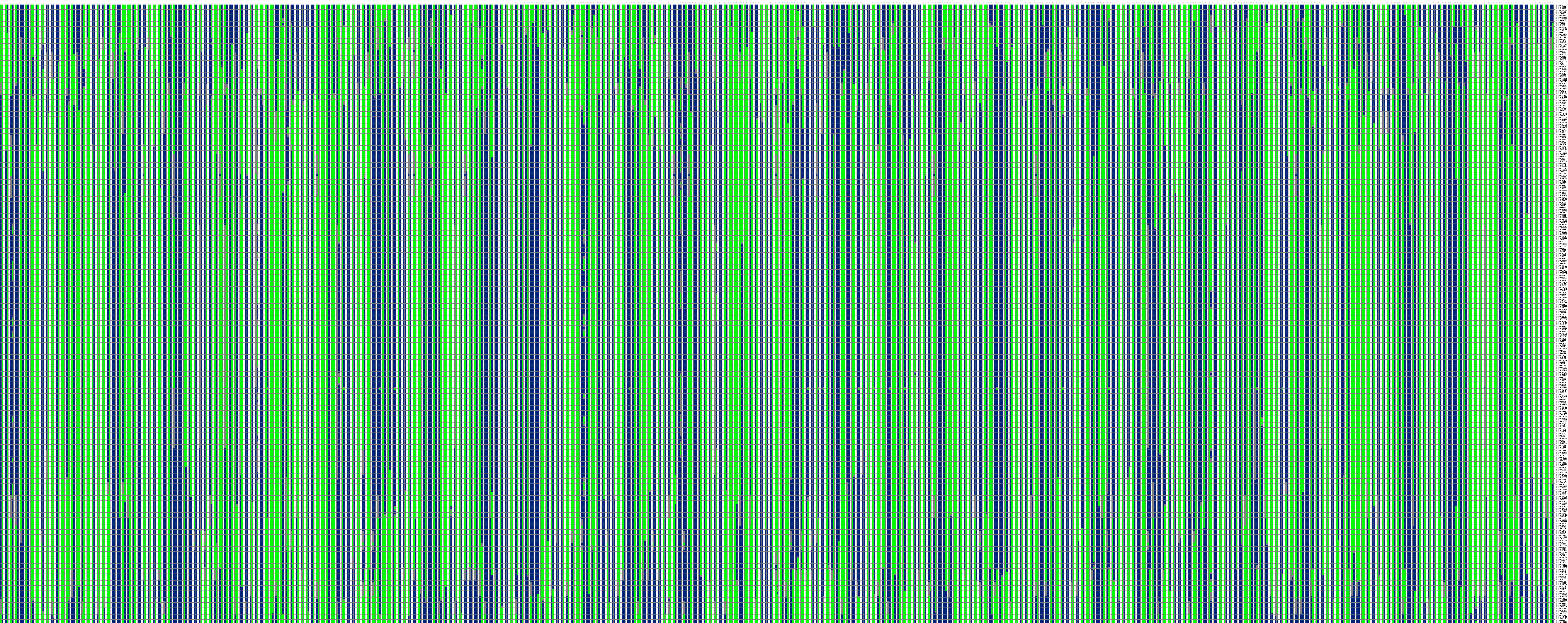
3c. LG3**

**
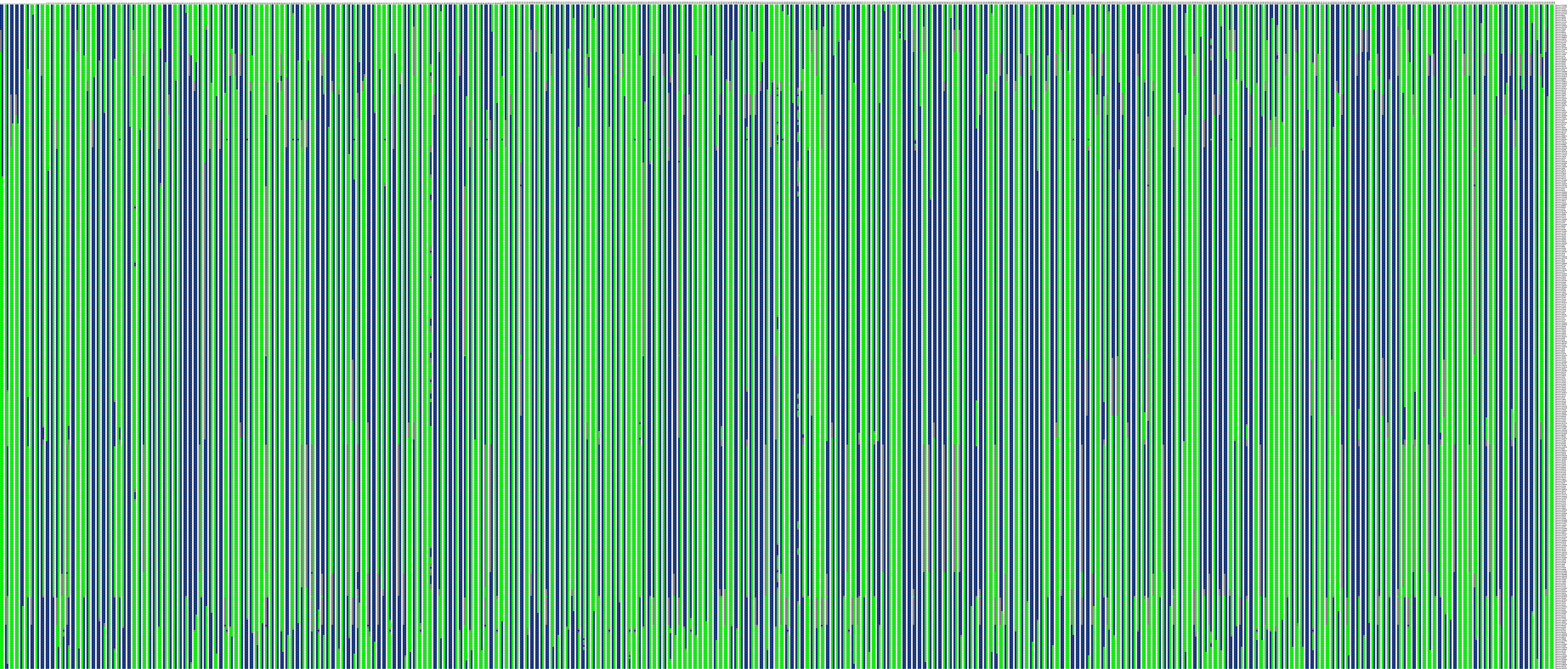
3d. LG4**

**3e. LG5**

**
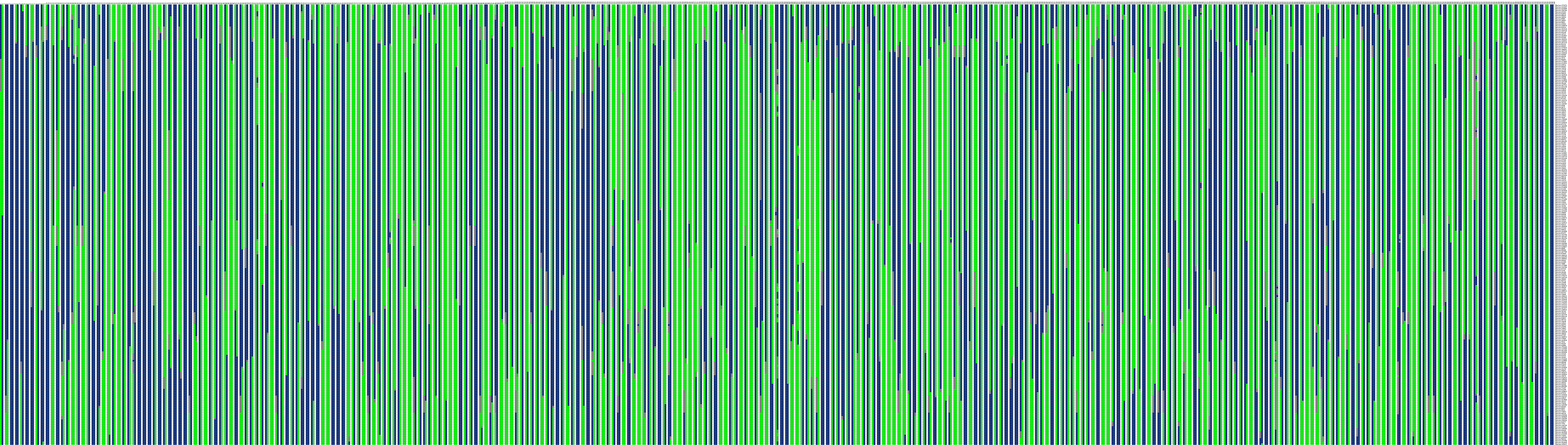
**

**
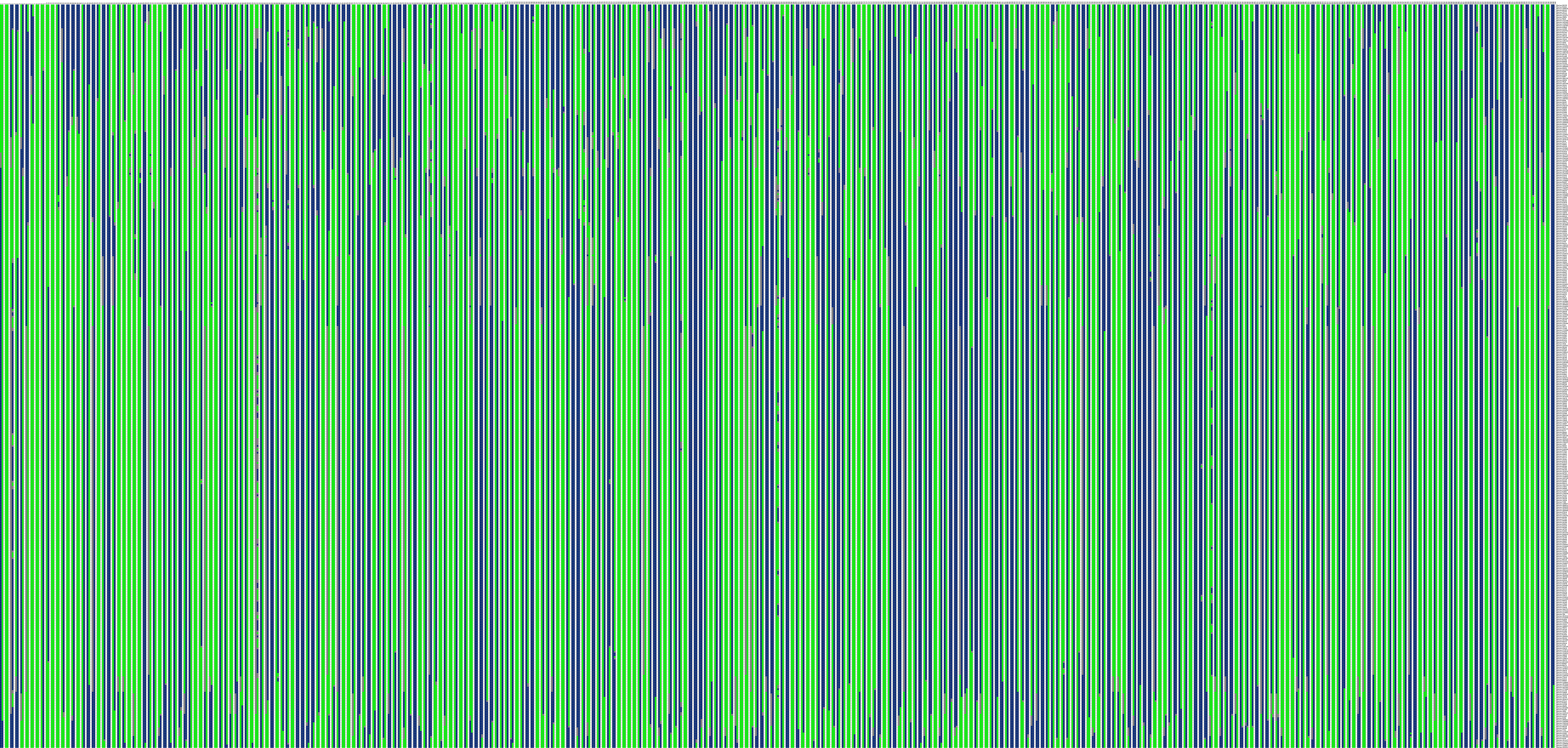
3f. LG6**

**
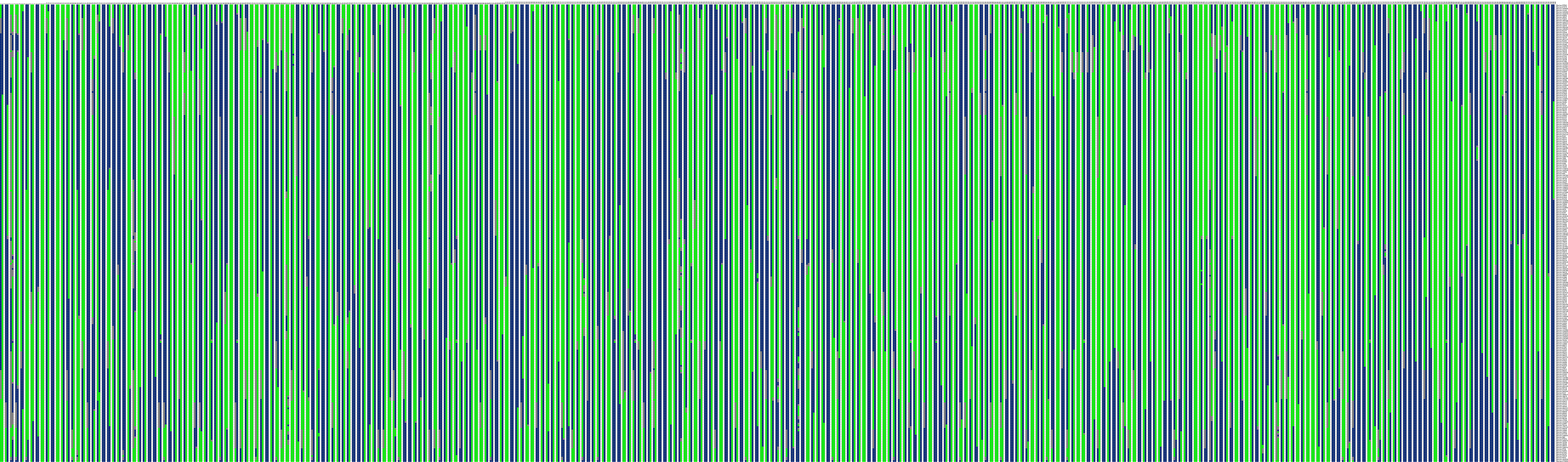
3g. LG7**

**
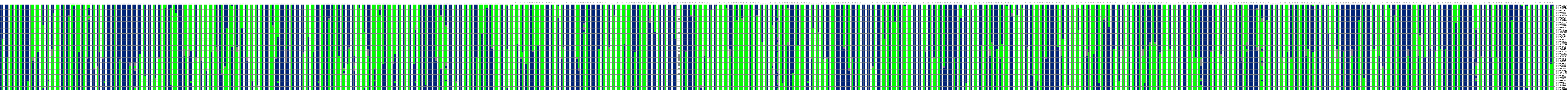
3h. LG8**


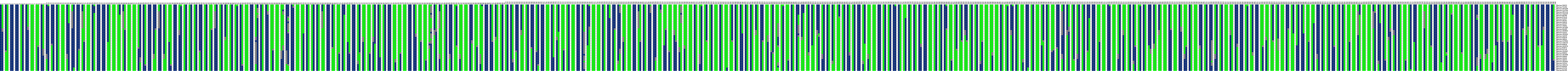
**3i. LG9**



**3j. LG10**

**3k. LG11**


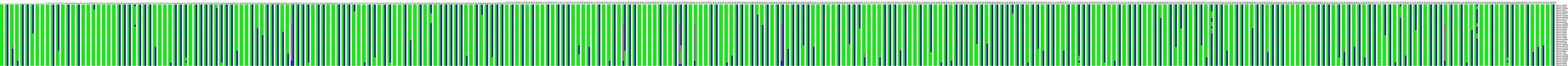


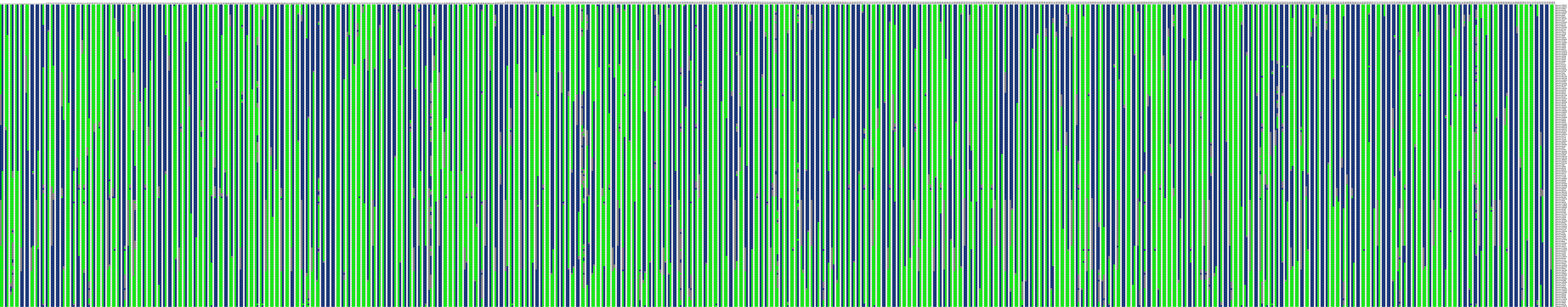
**3l. LG12**

**Figure S3(a-l)**

**
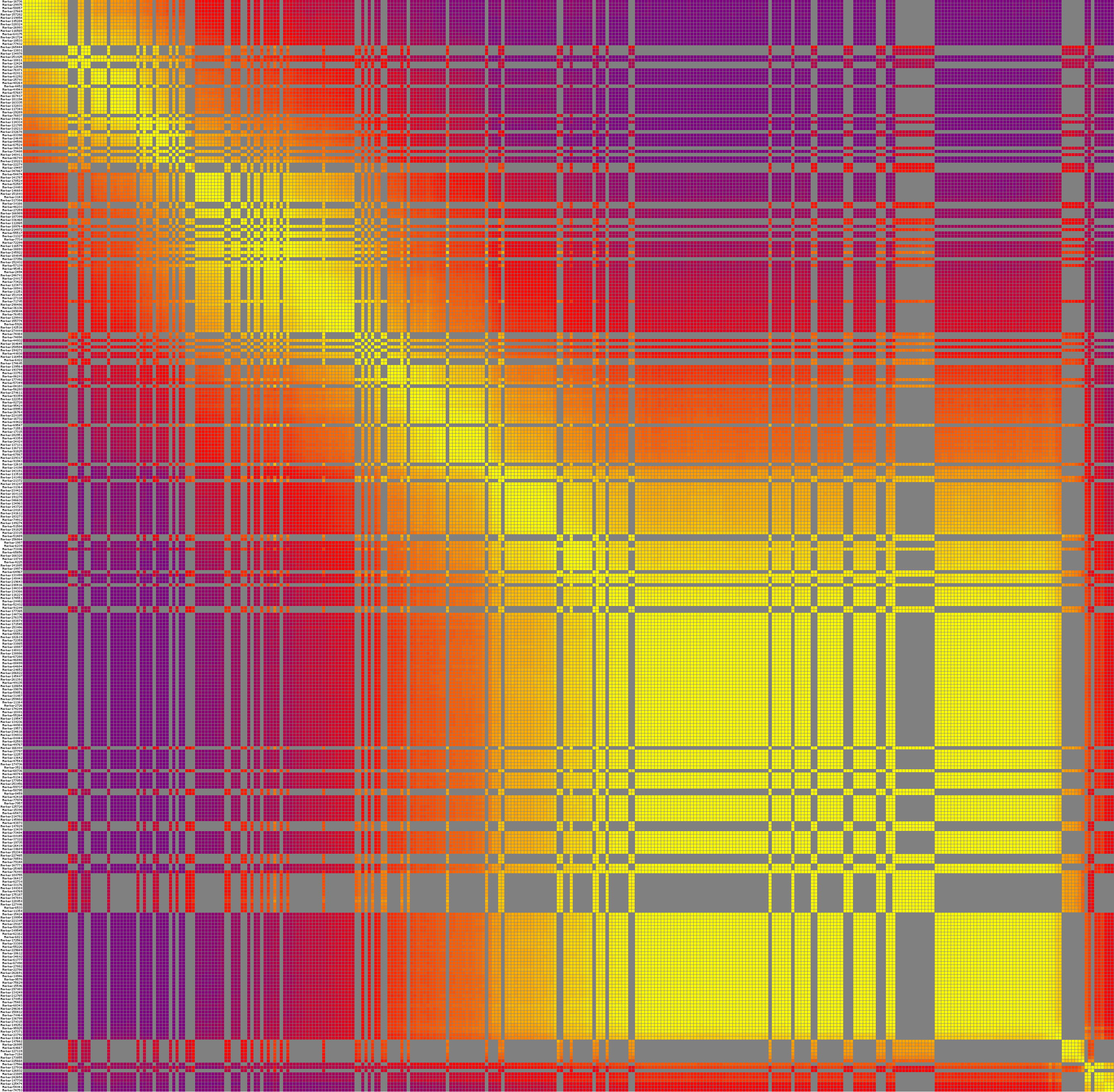
4a. LG1**


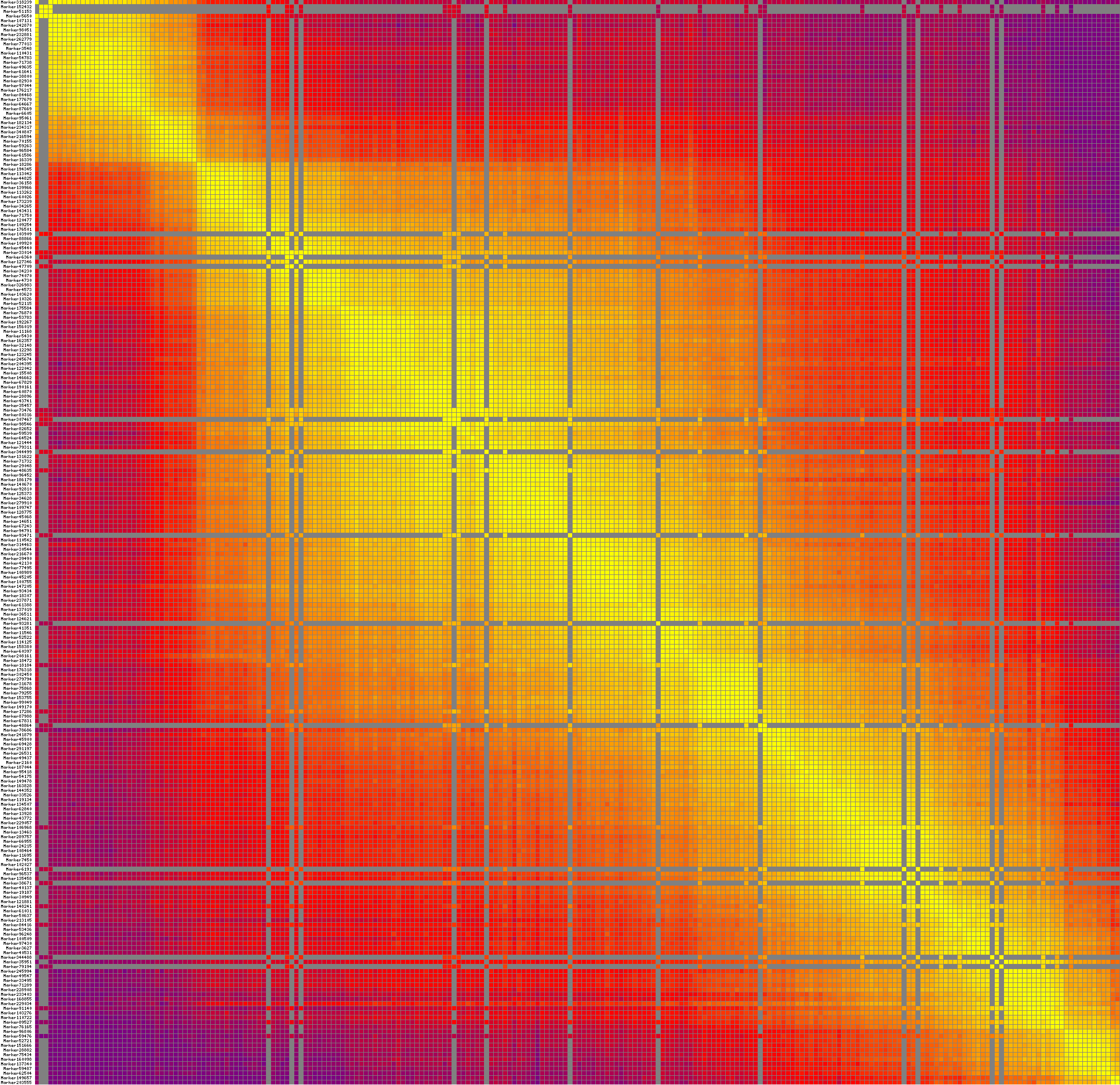
**4b. LG2**


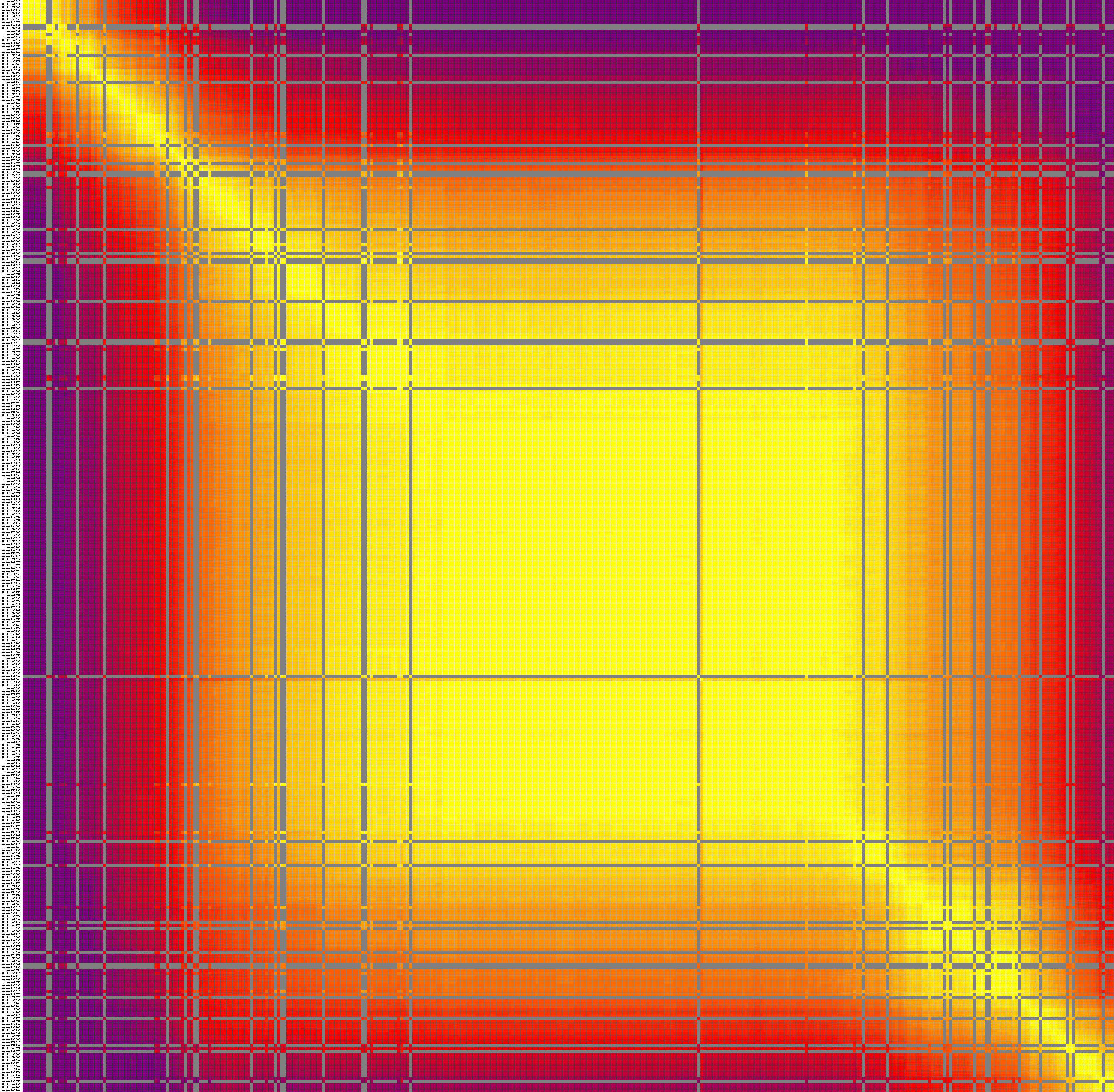
**4c. LG3**


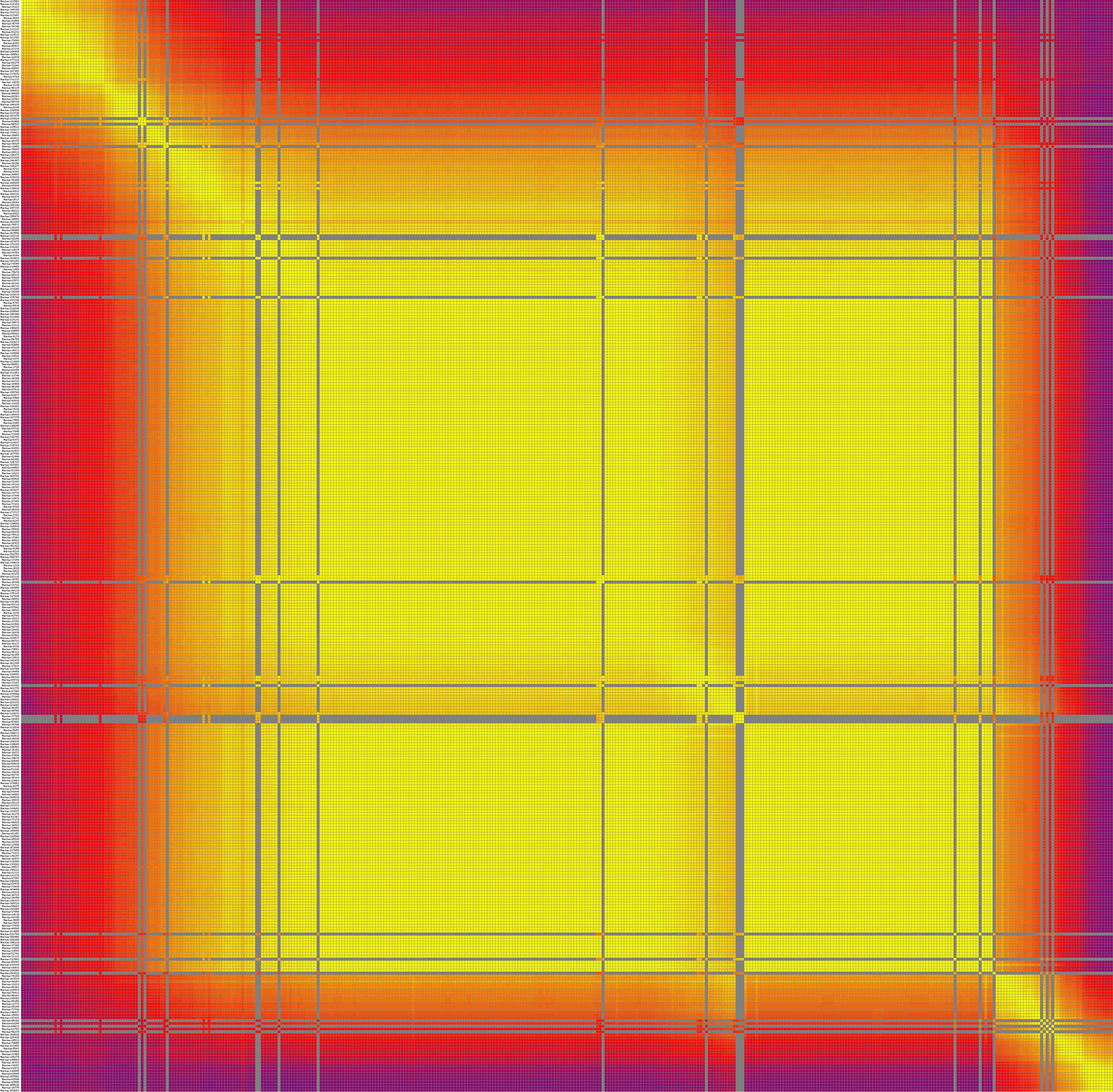
**4d. LG4**


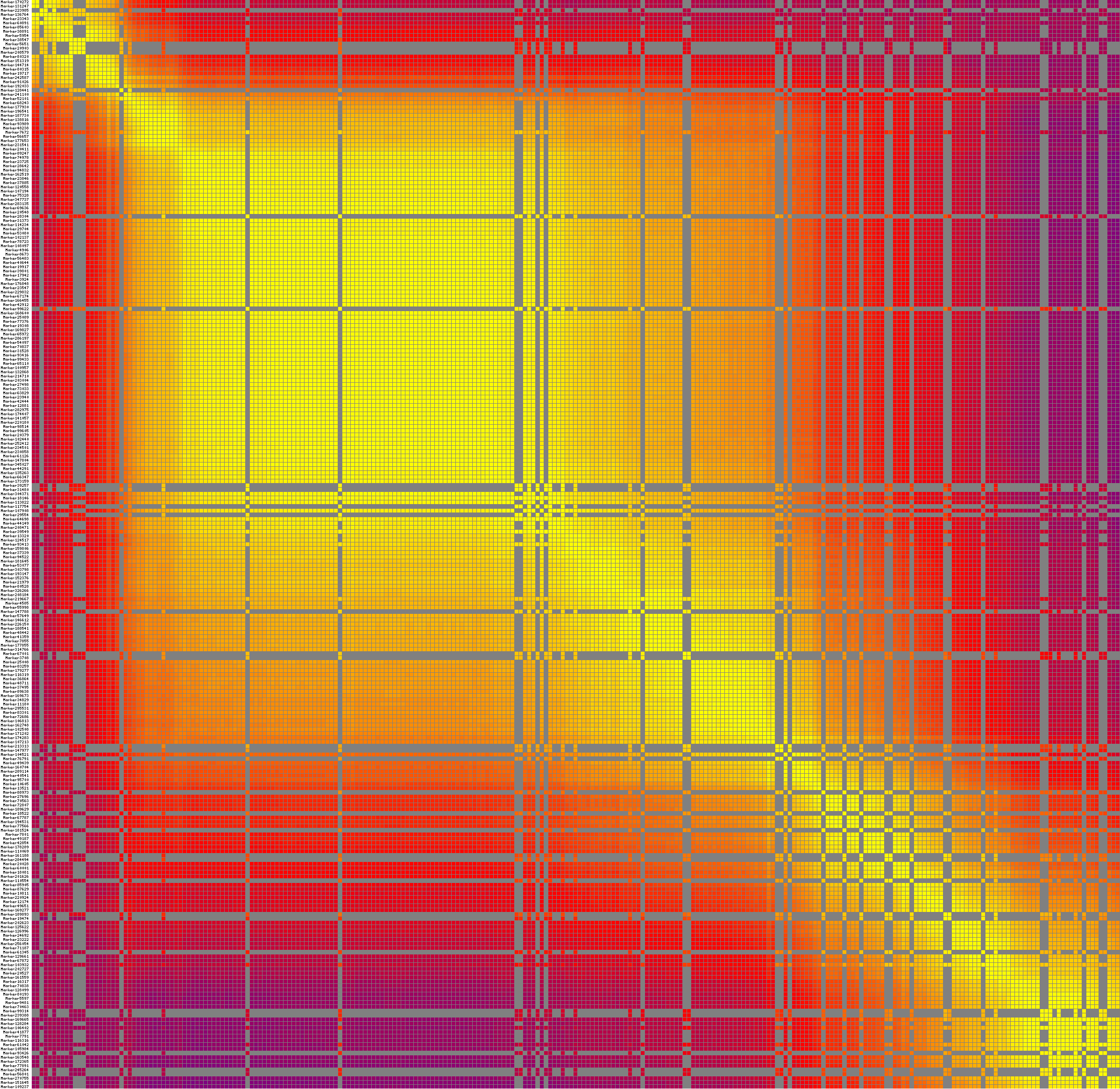
**4e. LG5**


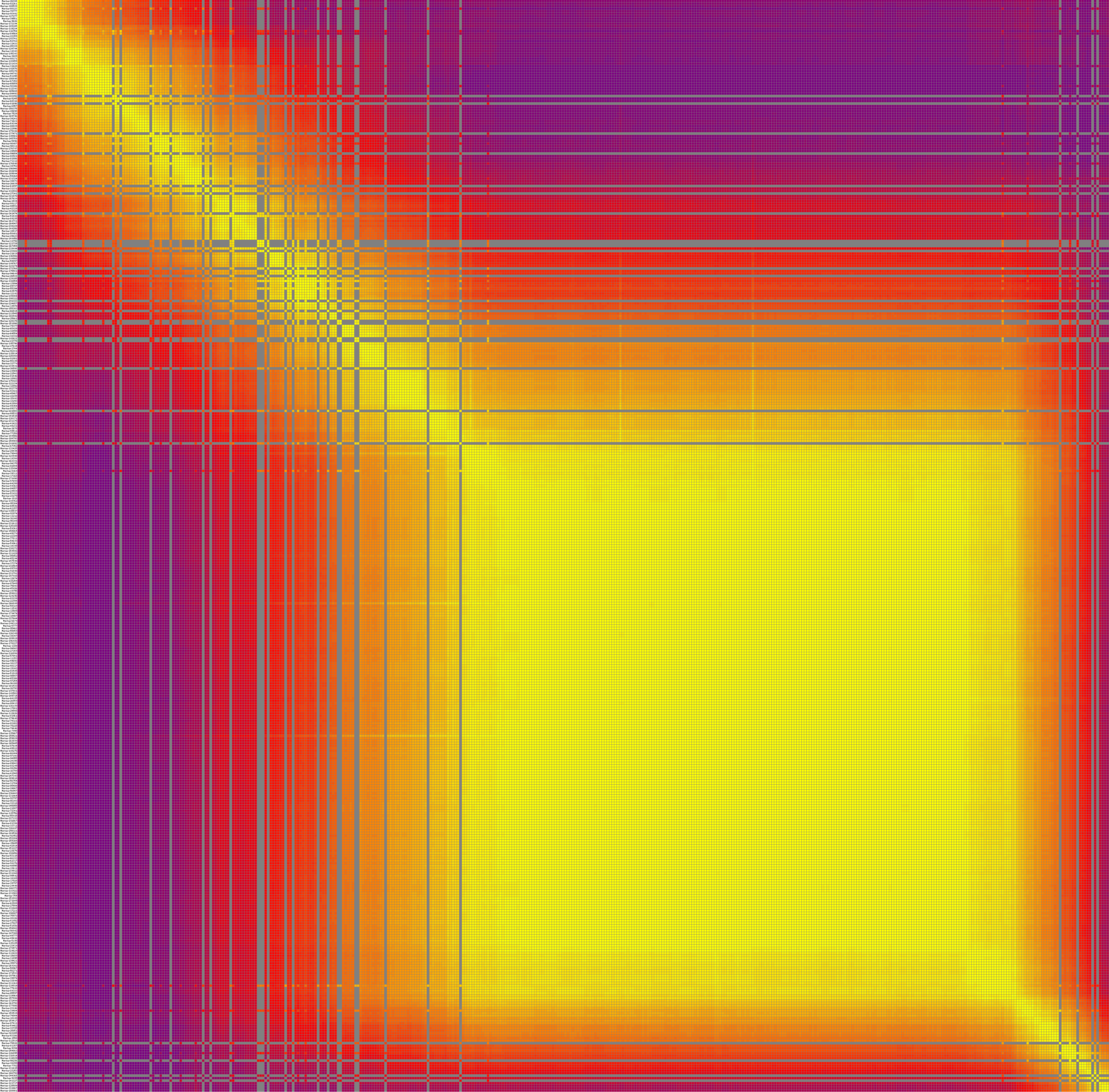
**4f. LG6**


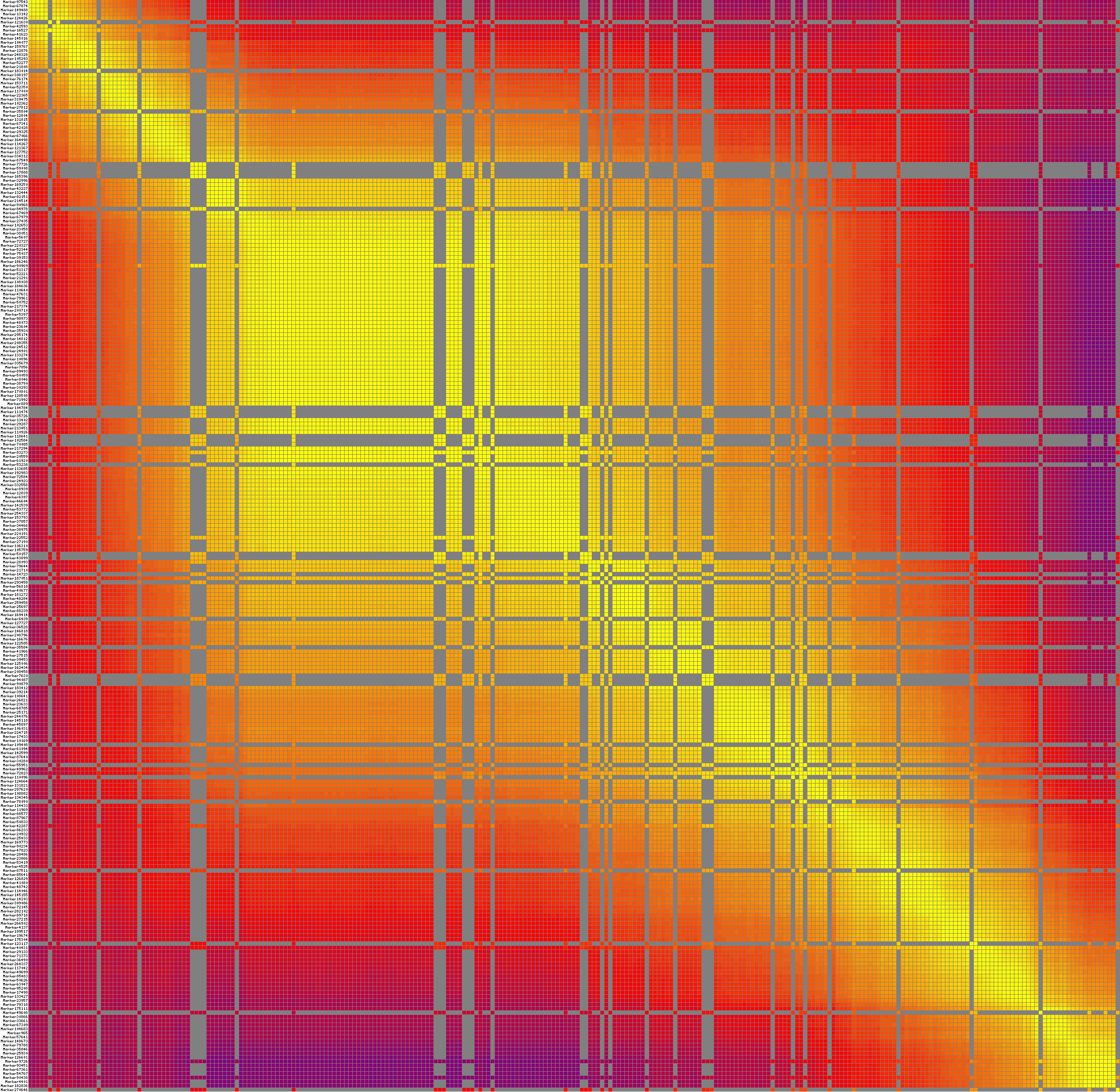
**4g. LG7**

**4h. LG8**


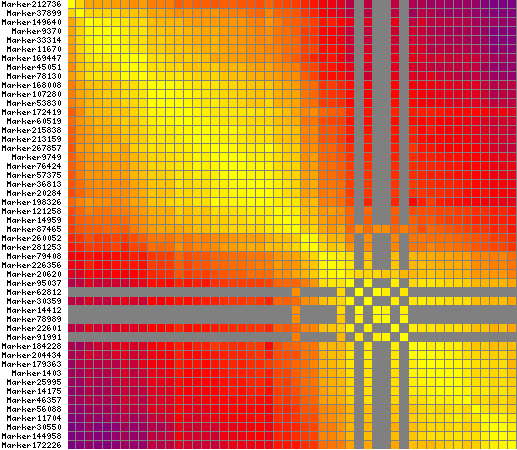


**4i. LG9**


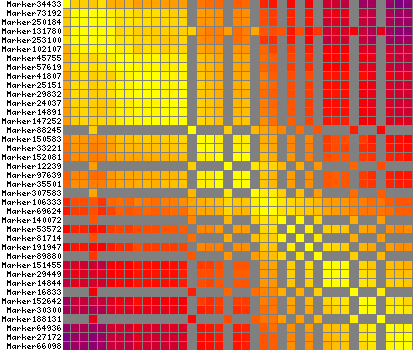



**4j. LG10**

**4k. LG11**


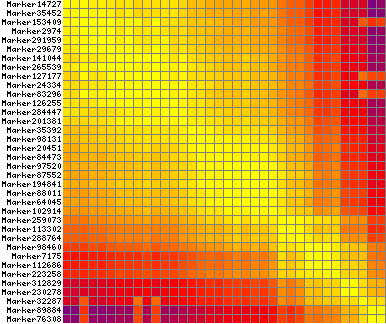


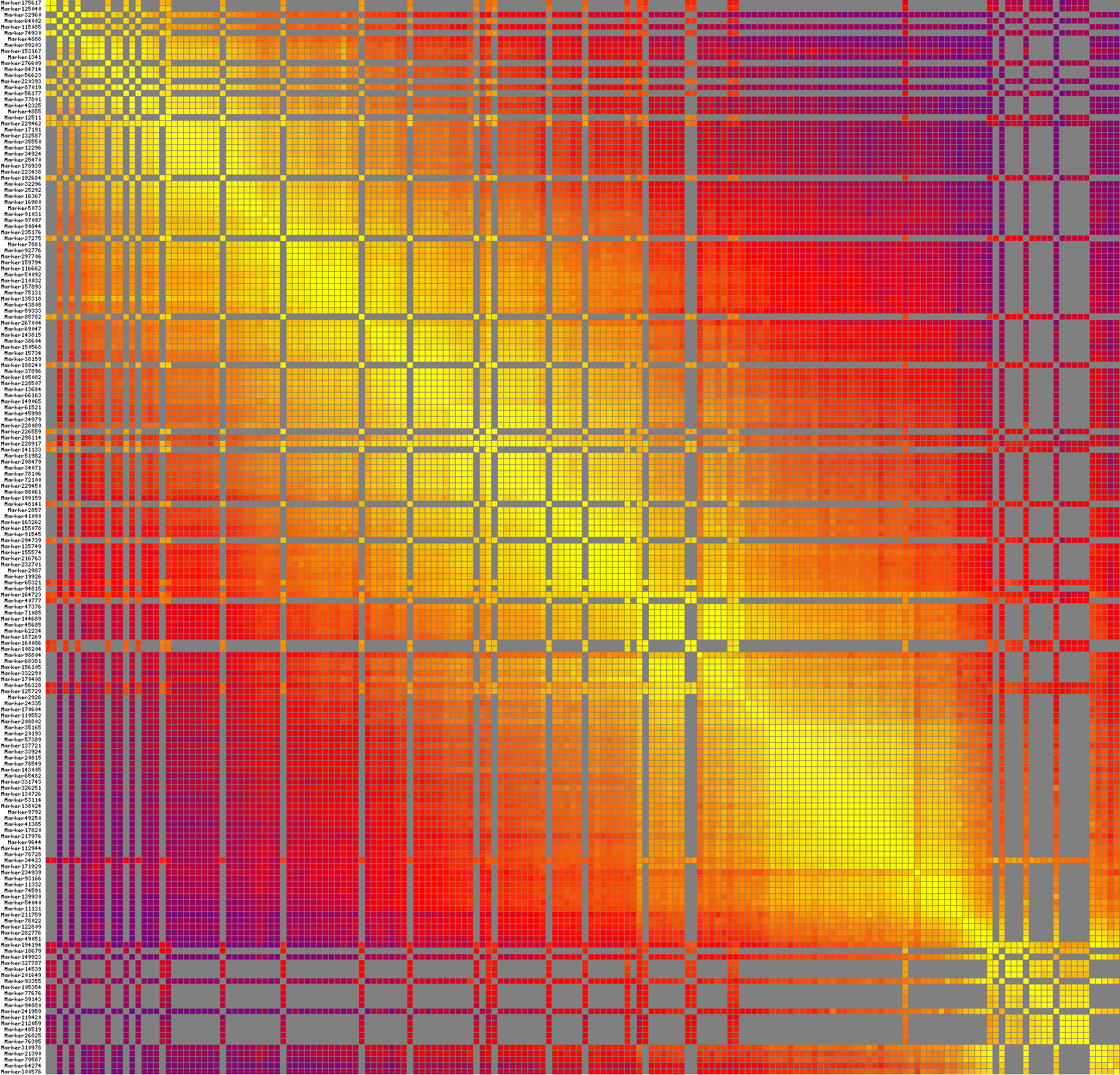
**4l. LG12**

**Figure S4(a-l)**
