# Supplementary figures and images for: Identification of fruit size associated quantitative trait loci featuring SLAF based high-density linkage map of goji berry (Lycium spp.)
Source: BMC Plant Biol. 2020 Oct 15;20:474. doi: 10.1186/s12870-020-02567-1 (PMC7565837; doi:10.1186/s12870-020-02567-1)

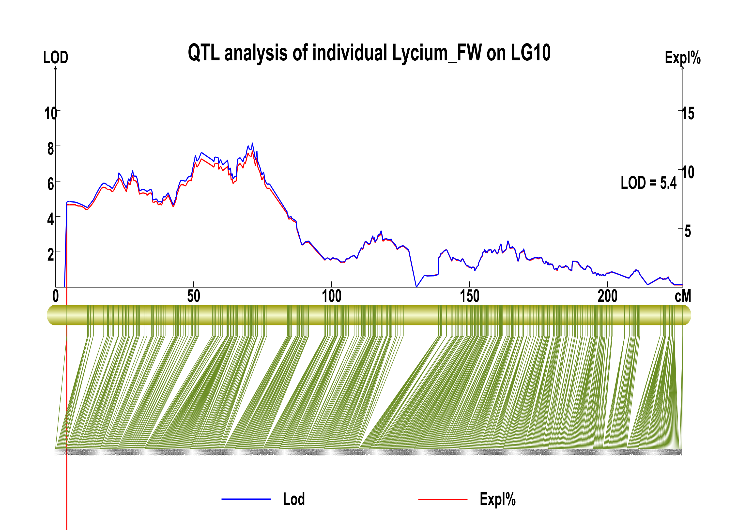

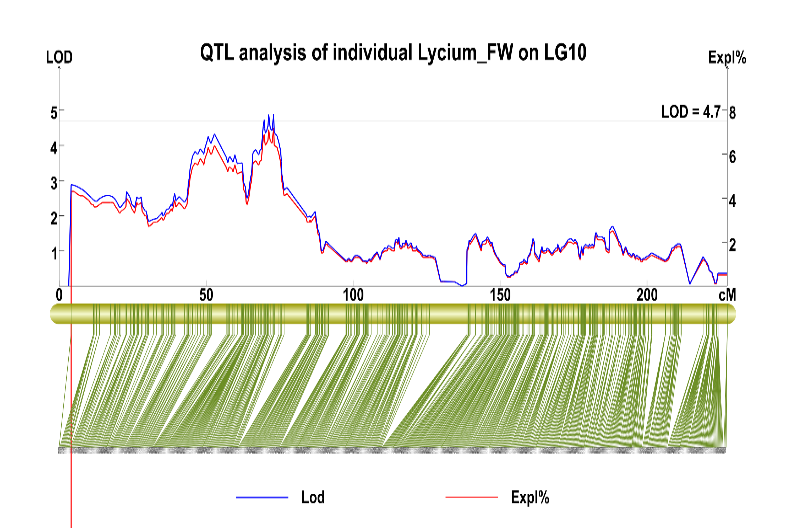

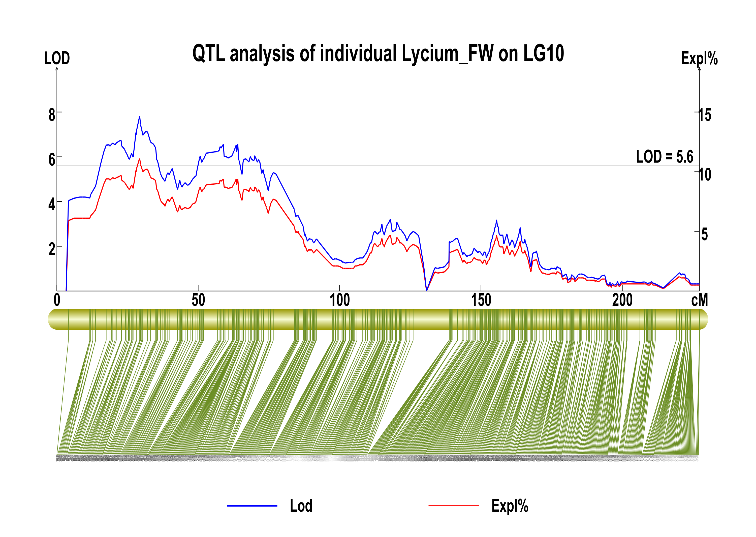
 **8a** **8b** **8c**

**
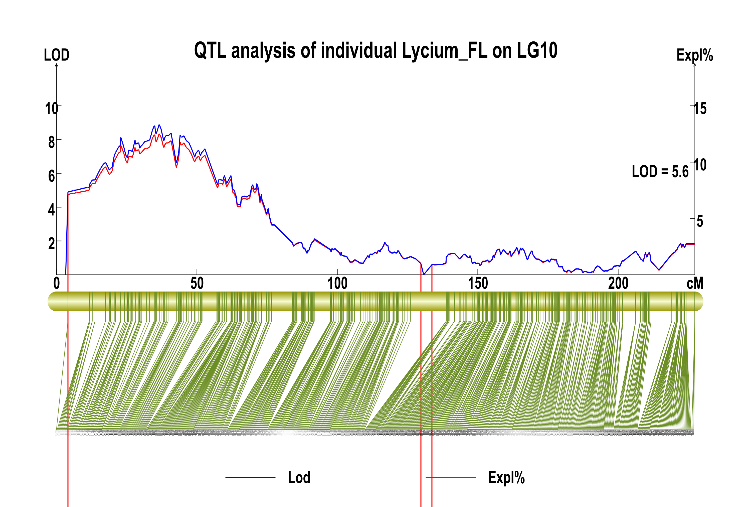

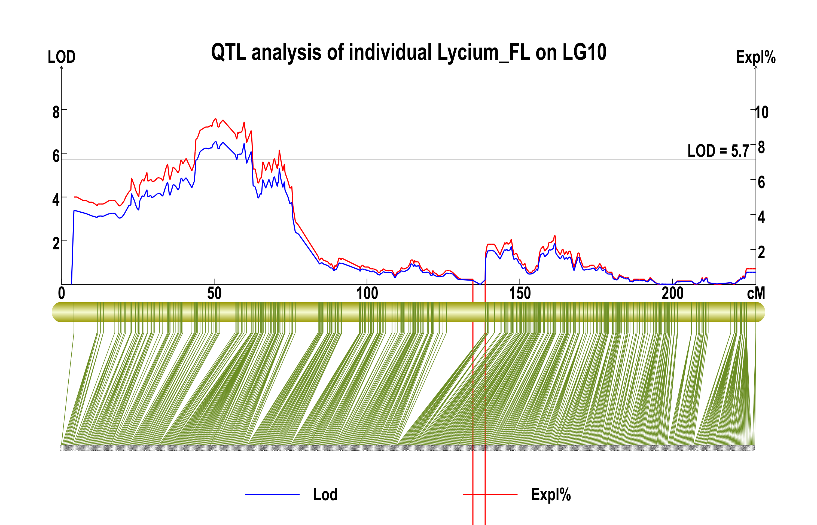

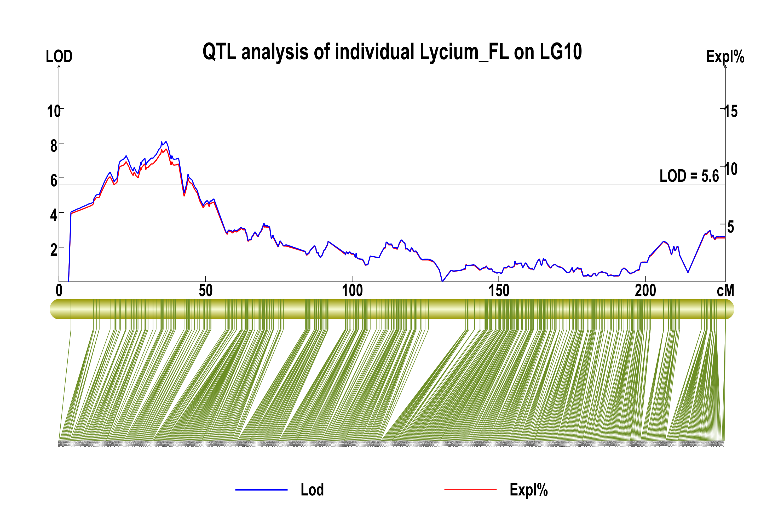
8d** **8e** **8f**

**8g** **8h** **8i**


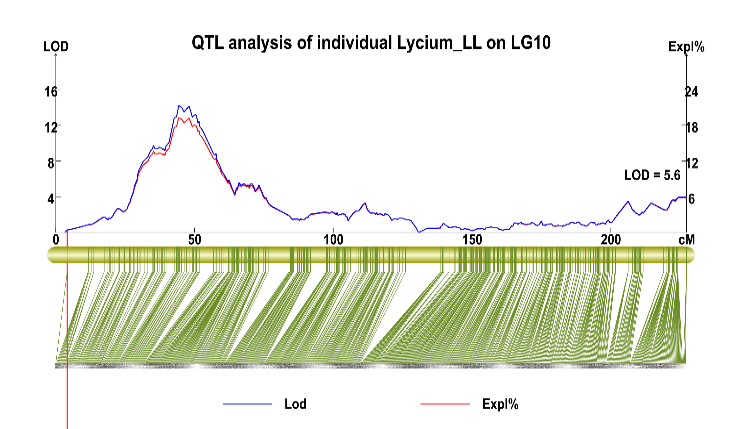

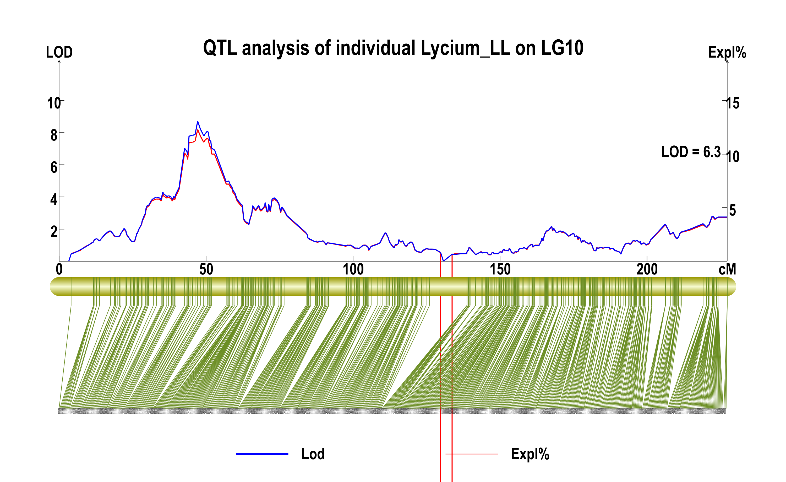

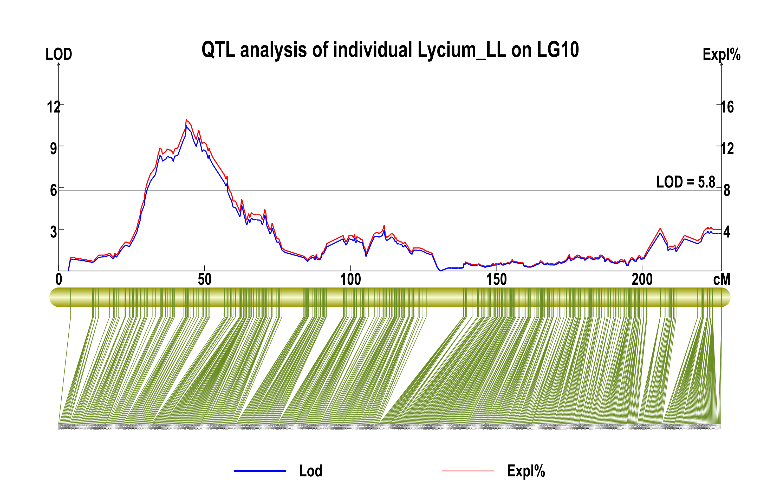


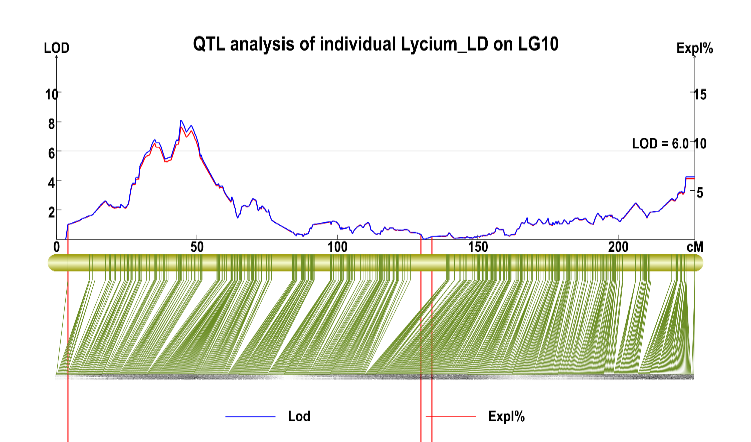

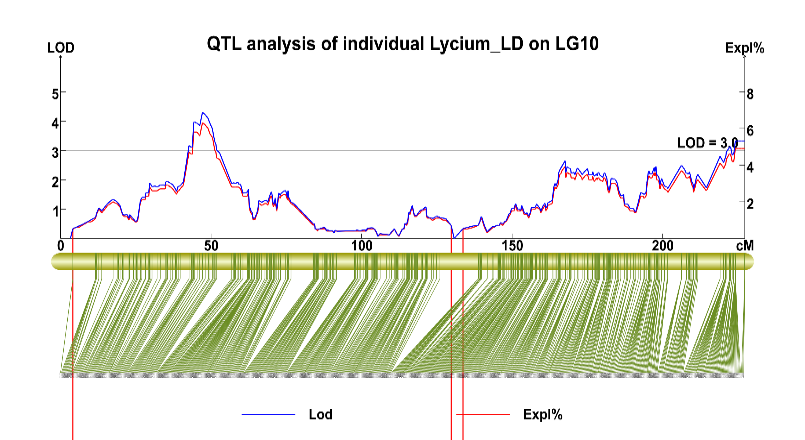

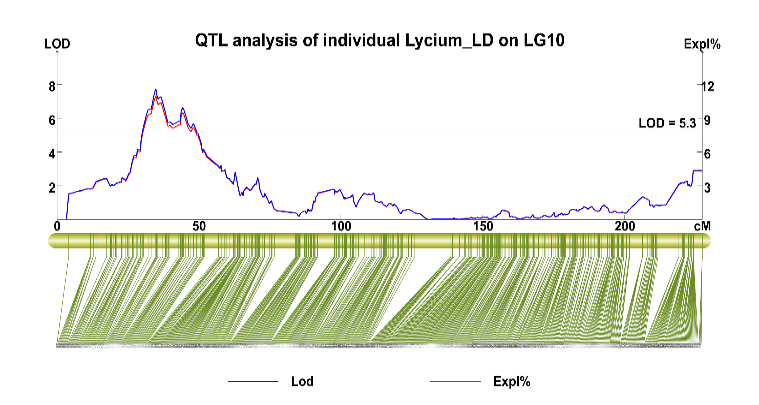
**8j** **8k** **8l**

**
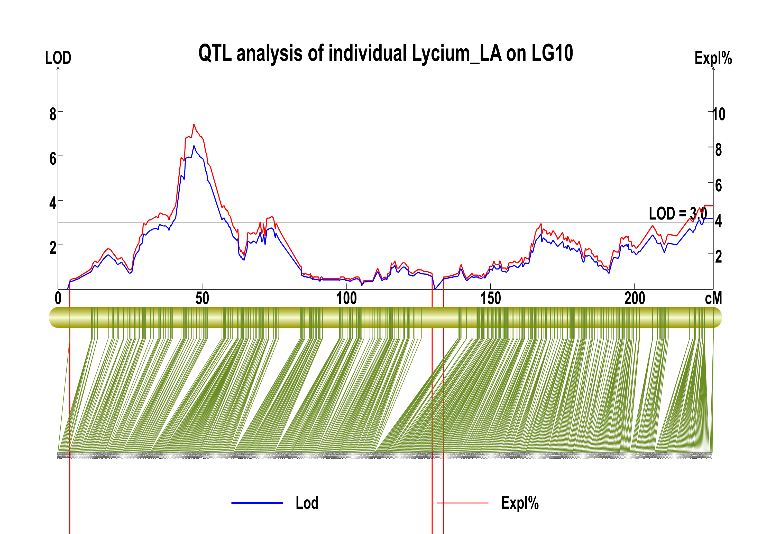

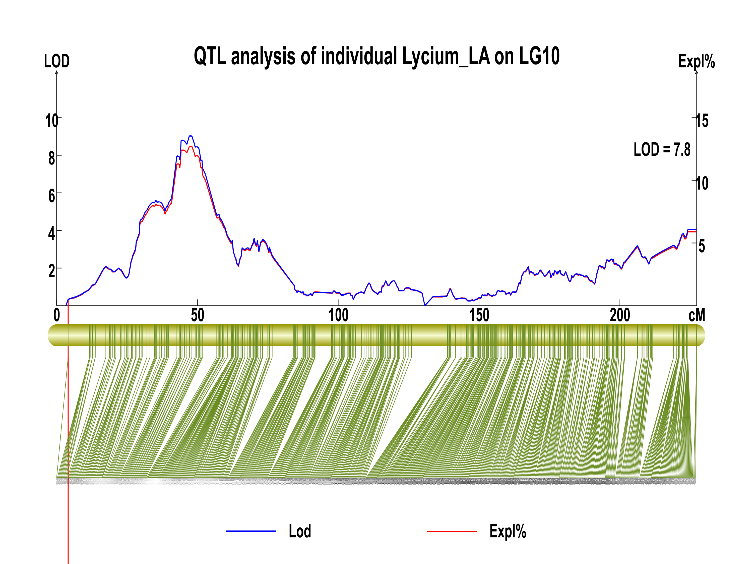

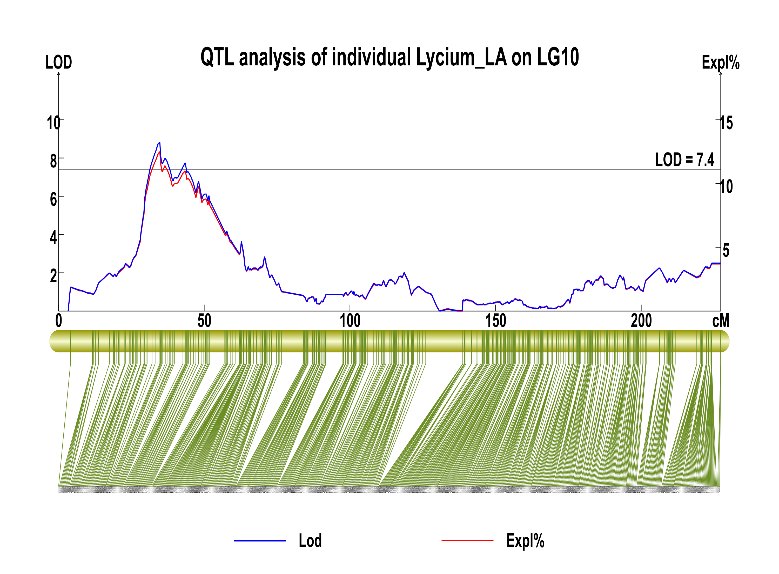
8m** **8n** **8o**

**Figure S8(a-o)**

Supplement: Supplementary file 3 — Additional file 3: Figure S8 (a-o). Stable QTLs map position on linkage group 10 (LG10) in 2 individual years (2018–2019) and extra year (1819). *left > 2018, center > 2019, right > 1819, FW_fruit weight, FL_fruit length, LL_leaf length, LD_leaf diameter, LA_leaf area. The red line shows phenotypic explained variation (Expl%), blue line LOD value, grey line LOD threshold value. [file 12870_2020_2567_MOESM3_ESM.docx]

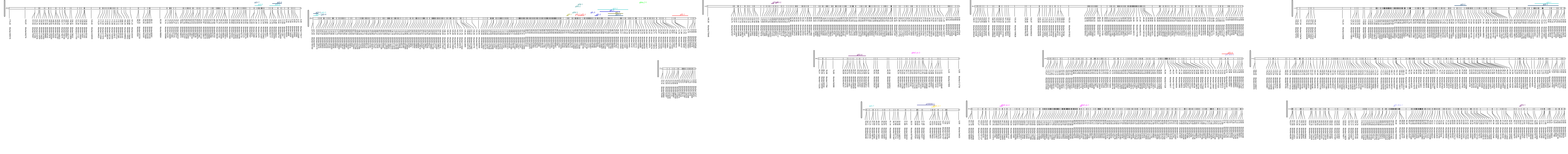

Supplement: Supplementary file 8 — Additional file 8: Figure S6. QTL mapping on integrated linkage map in 2019. *The different color pattern represents identified QTLs for agronomic traits of goji berry such as FW_fruit weight, FL_fruit length, FSI_fruit shape index, Nof/ec_ number of fruits per end cluster, Nof/n_ number of fruits per node, Nos/f_ number of seeds per fruit, 100SW_100 seed weight, LL_leaf length, LD_leaf diameter, LA_leaf area, FS_ fruit sweetness (obrix) and FF_ fruit firmness [file 12870_2020_2567_MOESM8_ESM.png]

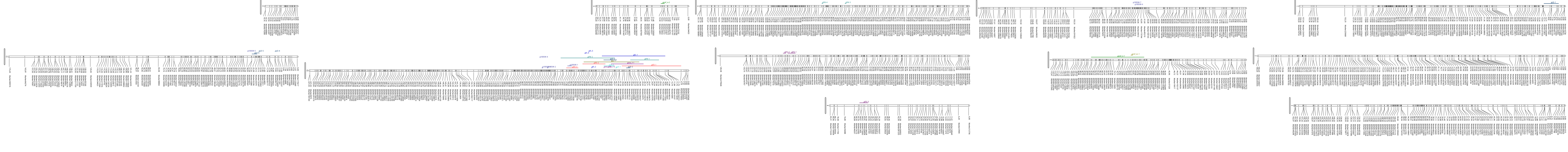

Supplement: Supplementary file 9 — Additional file 9: Figure S7. QTL mapping on integrated linkage map in 1819. *The different color pattern represents identified QTLs for agronomic traits of goji berry such as FW_fruit weight, FL_fruit length, FSI_fruit shape index, Nof/ec_ number of fruits per end cluster, Nof/n_ number of fruits per node, Nos/f_ number of seeds per fruit, 100SW_100 seed weight, LL_leaf length, LD_leaf diameter, LA_leaf area. [file 12870_2020_2567_MOESM9_ESM.png]
